# Supplementary material for: Secreted exosomes induce filopodia formation
Source: eLife. 2026 Jan 14;13:RP101673. doi: 10.7554/eLife.101673 (PMC12803517; doi:10.7554/eLife.101673)
Supplement: Figure 2—figure supplement 1—source data 1. [file elife-101673-fig2-figsupp1-data1.zip › Figure 2_Figure Supplement 1_Source Data 1.pdf]

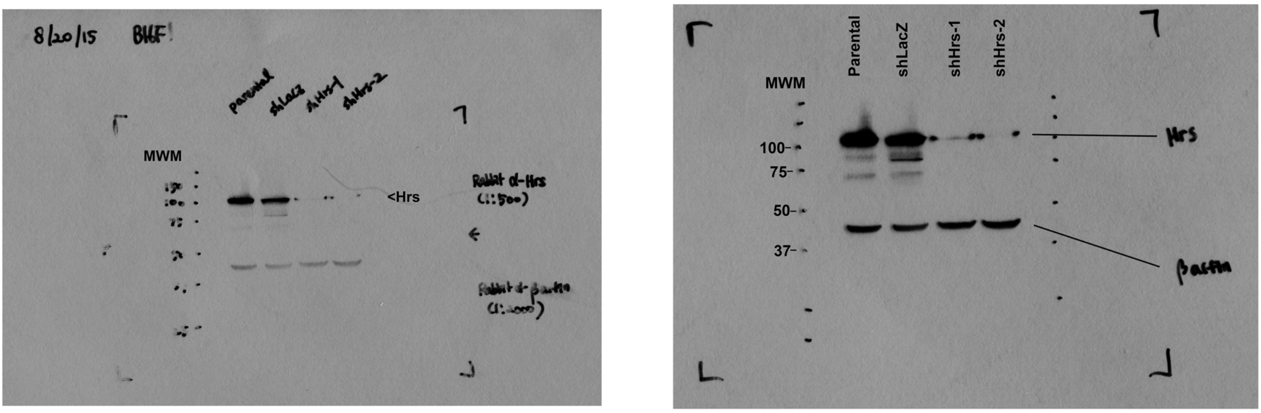


**Figure 2, Figure Supplement 1, Source data 1.** Original exposures on film of membranes corresponding to Figure 2, Figure supplement 1, panel A. Rainbow molecular weight markers were employed and noted on the film with marker.
