# Supplementary figures and images for: Secreted exosomes induce filopodia formation
Source: eLife. 2026 Jan 14;13:RP101673. doi: 10.7554/eLife.101673 (PMC12803517; doi:10.7554/eLife.101673)

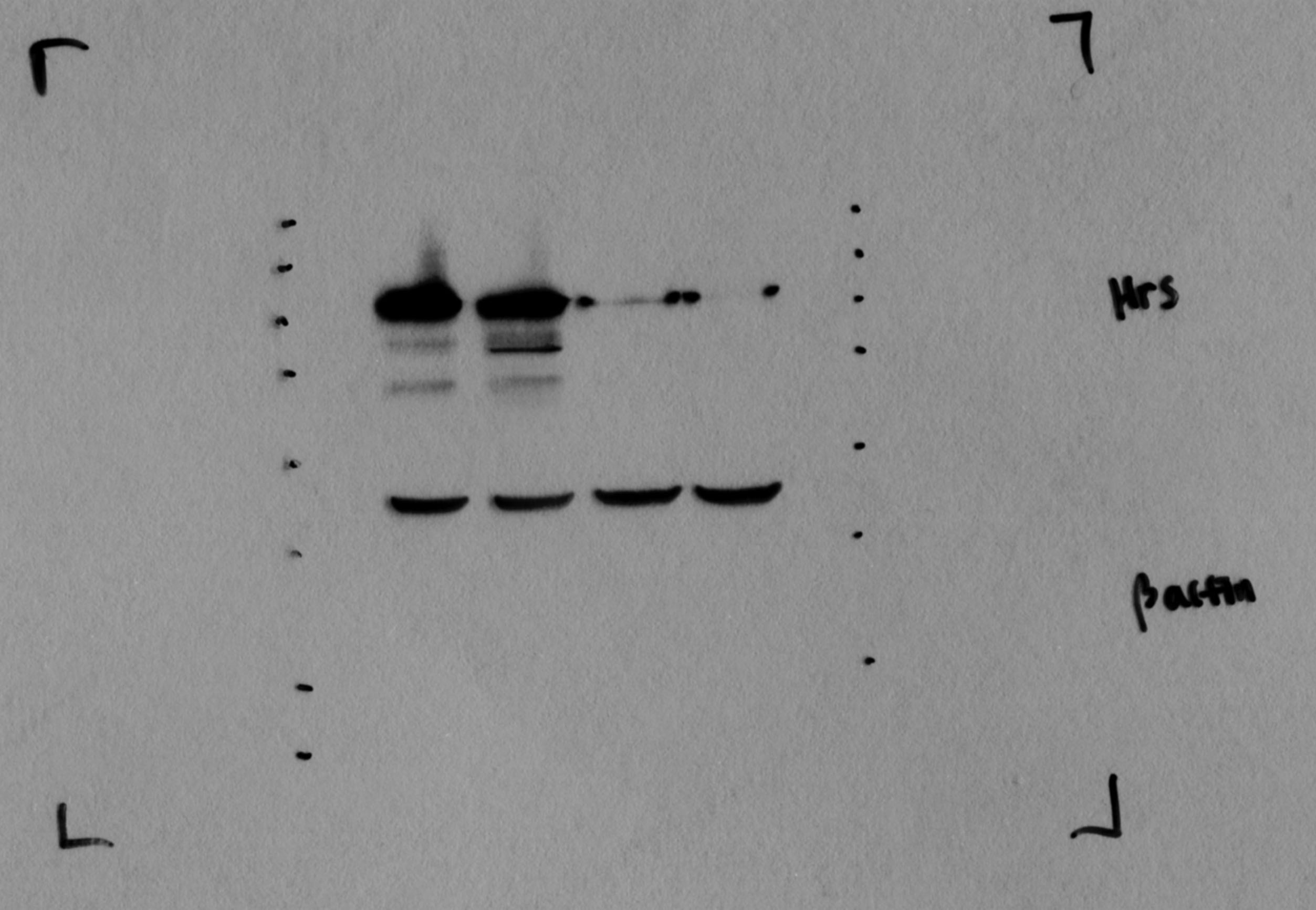

Supplement: Figure 2—figure supplement 1—source data 2. [file elife-101673-fig2-figsupp1-data2.zip › actin raw unedited.tiff]

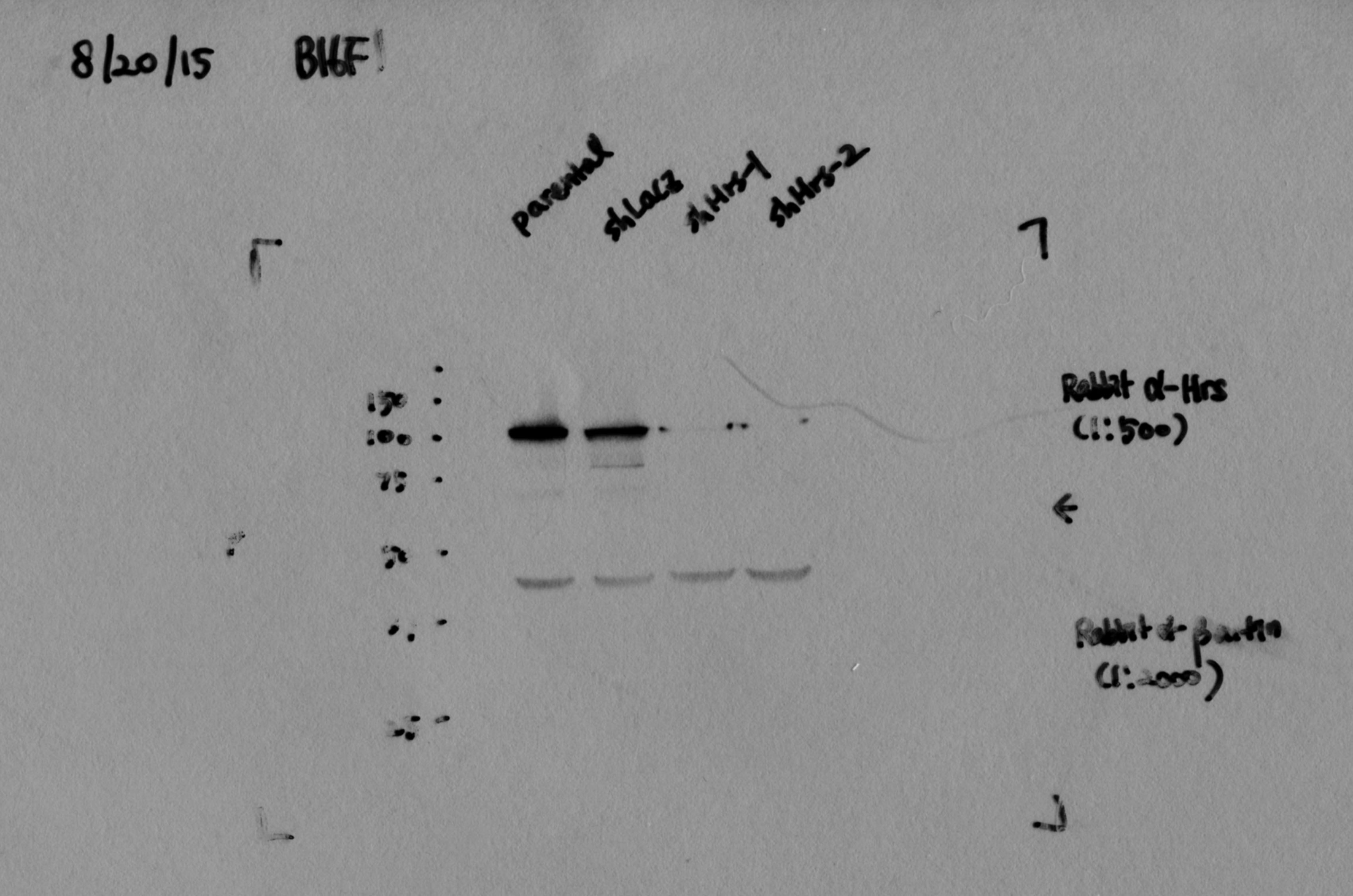

Supplement: Figure 2—figure supplement 1—source data 2. [file elife-101673-fig2-figsupp1-data2.zip › hrs raw unedited.tiff]

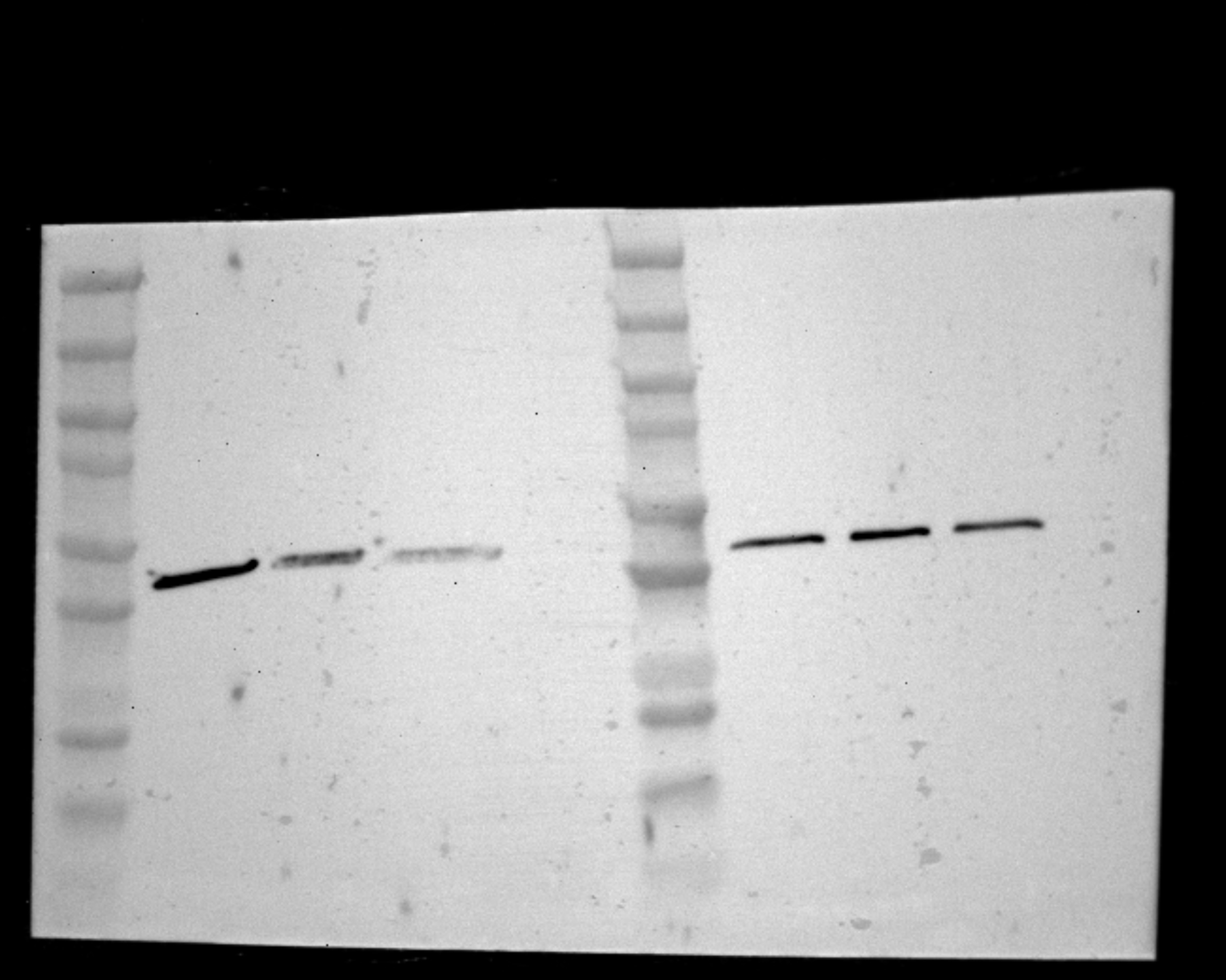

Supplement: Figure 2—figure supplement 1—source data 4. [file elife-101673-fig2-figsupp1-data4.zip › actin raw unedited.tiff]

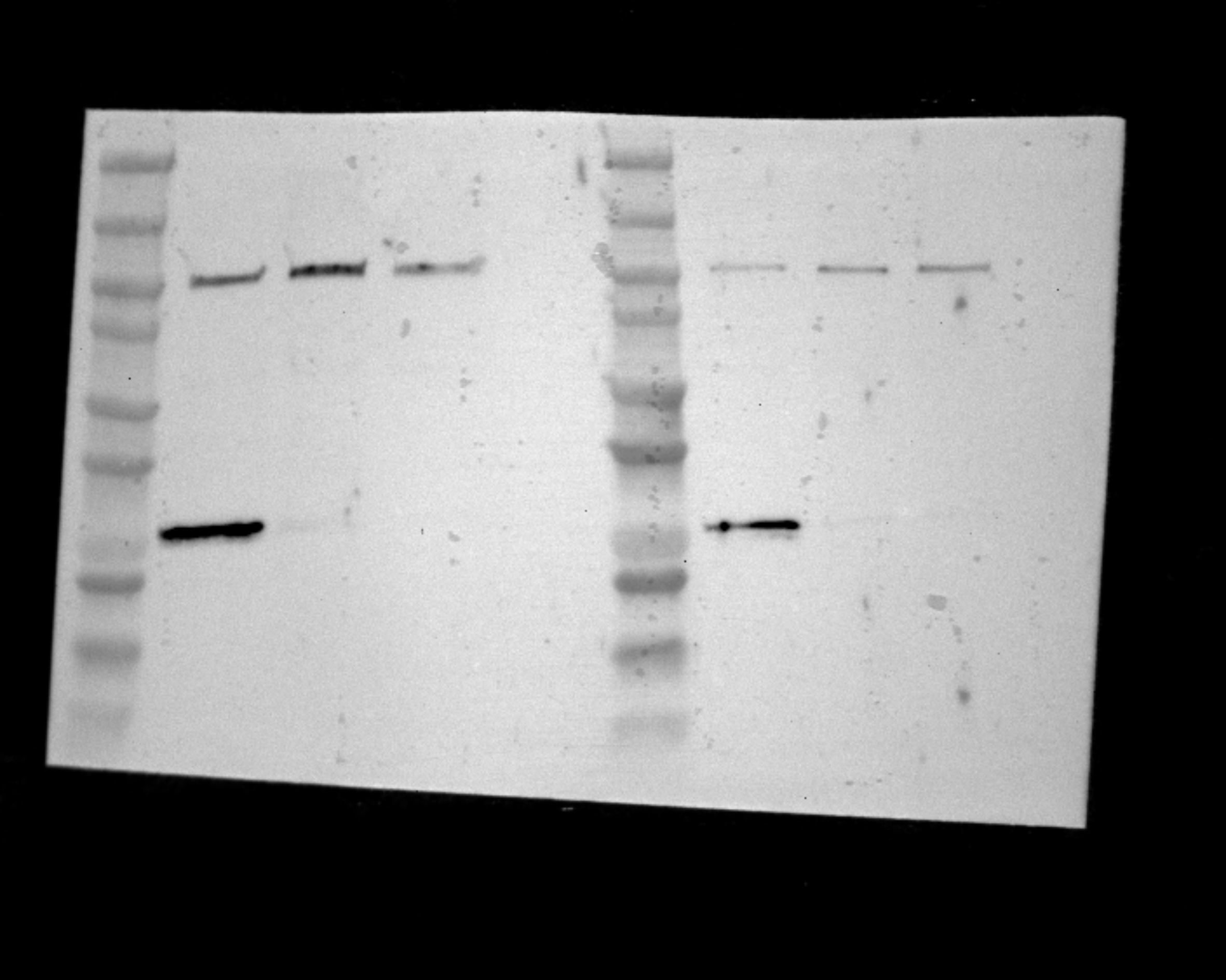

Supplement: Figure 2—figure supplement 1—source data 4. [file elife-101673-fig2-figsupp1-data4.zip › rab27a raw unedited.tiff]

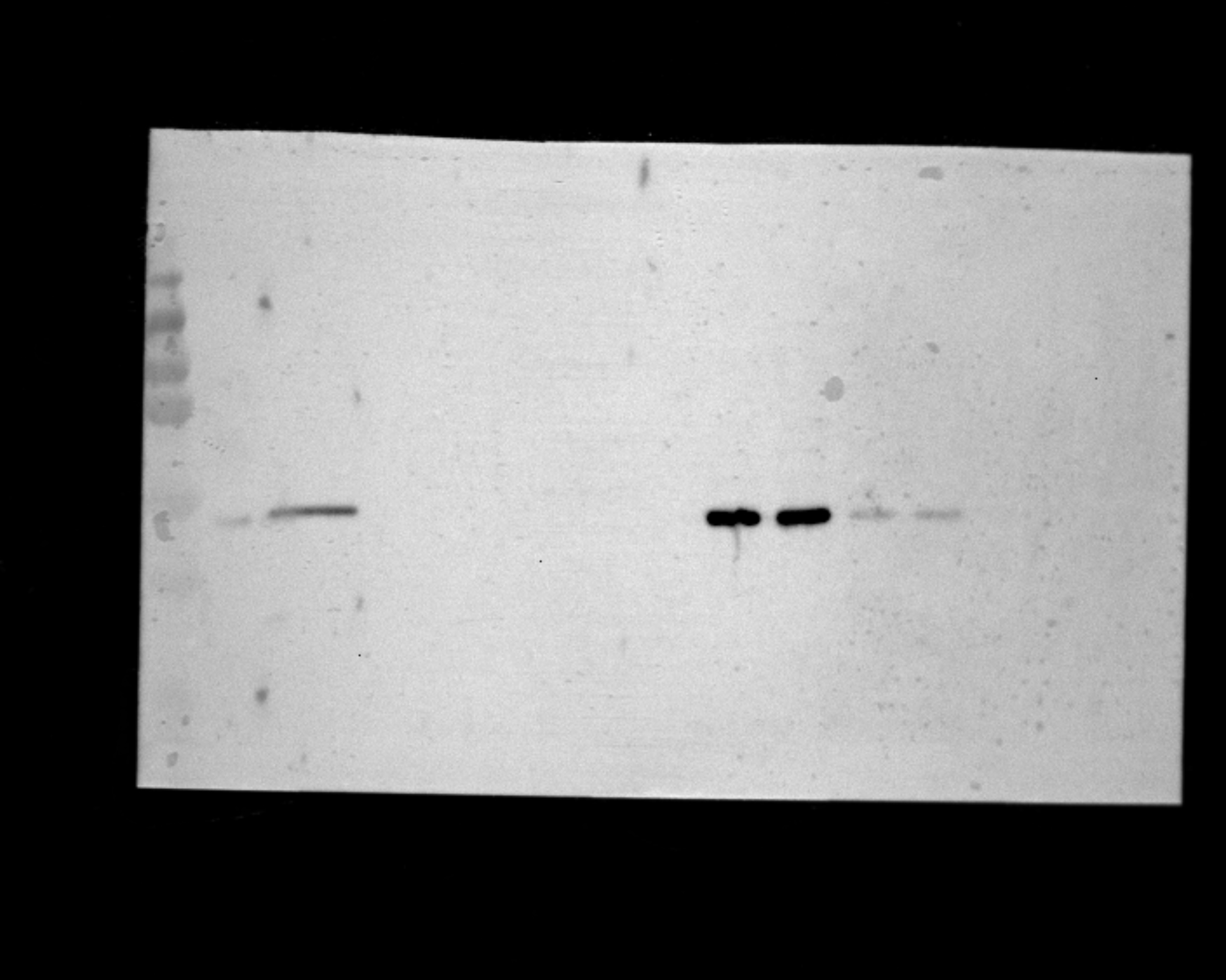

Supplement: Figure 2—figure supplement 1—source data 6. [file elife-101673-fig2-figsupp1-data6.zip › flot1 raw unedited.tiff]

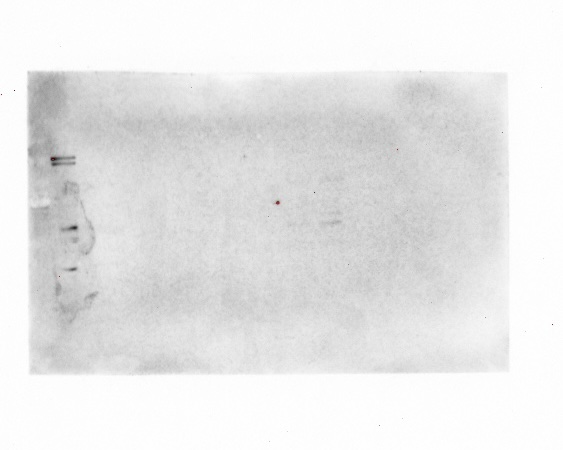

Supplement: Figure 2—figure supplement 1—source data 6. [file elife-101673-fig2-figsupp1-data6.zip › gm130 raw unedited.tiff]

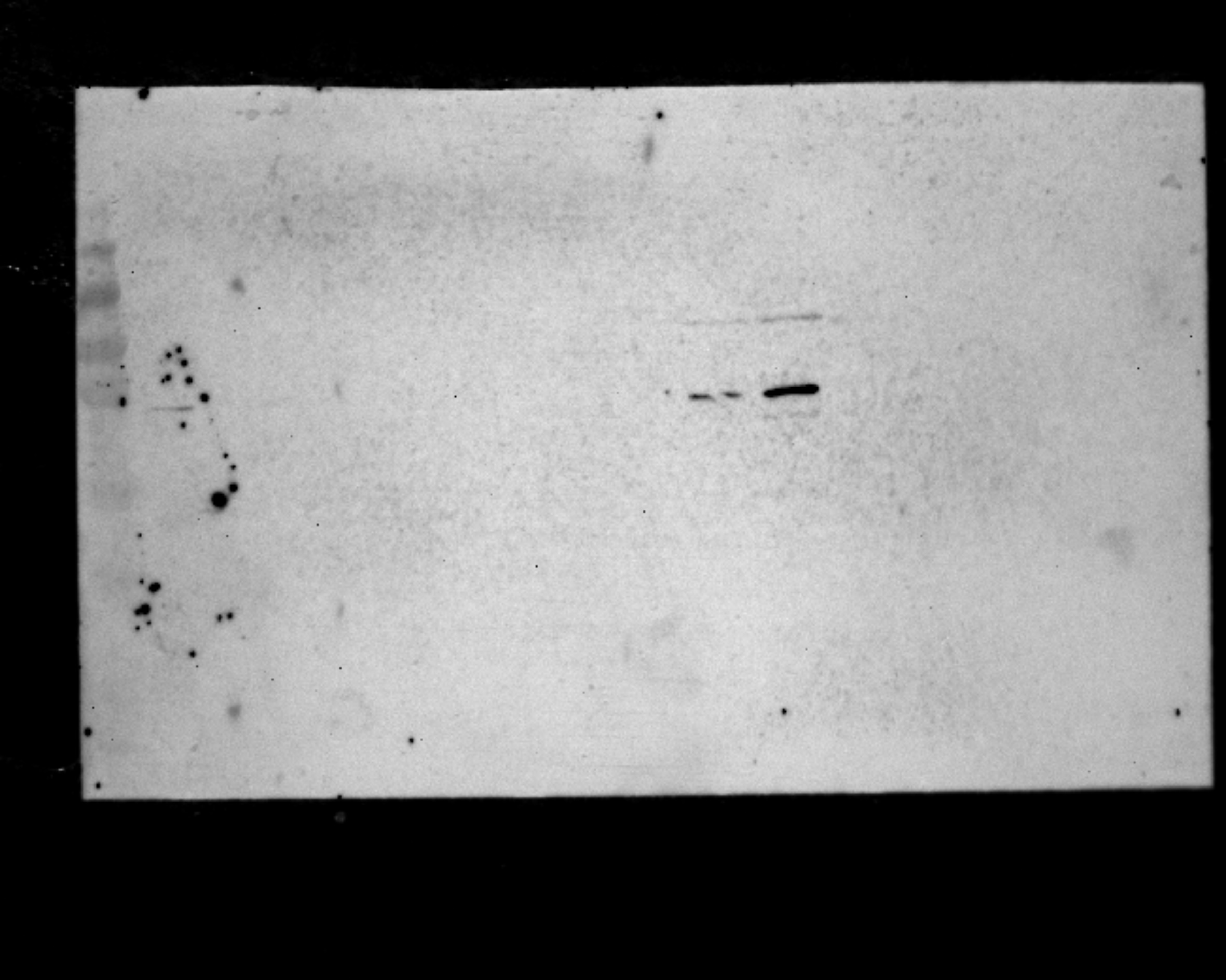

Supplement: Figure 2—figure supplement 1—source data 6. [file elife-101673-fig2-figsupp1-data6.zip › hsp70 raw unedited.tiff]

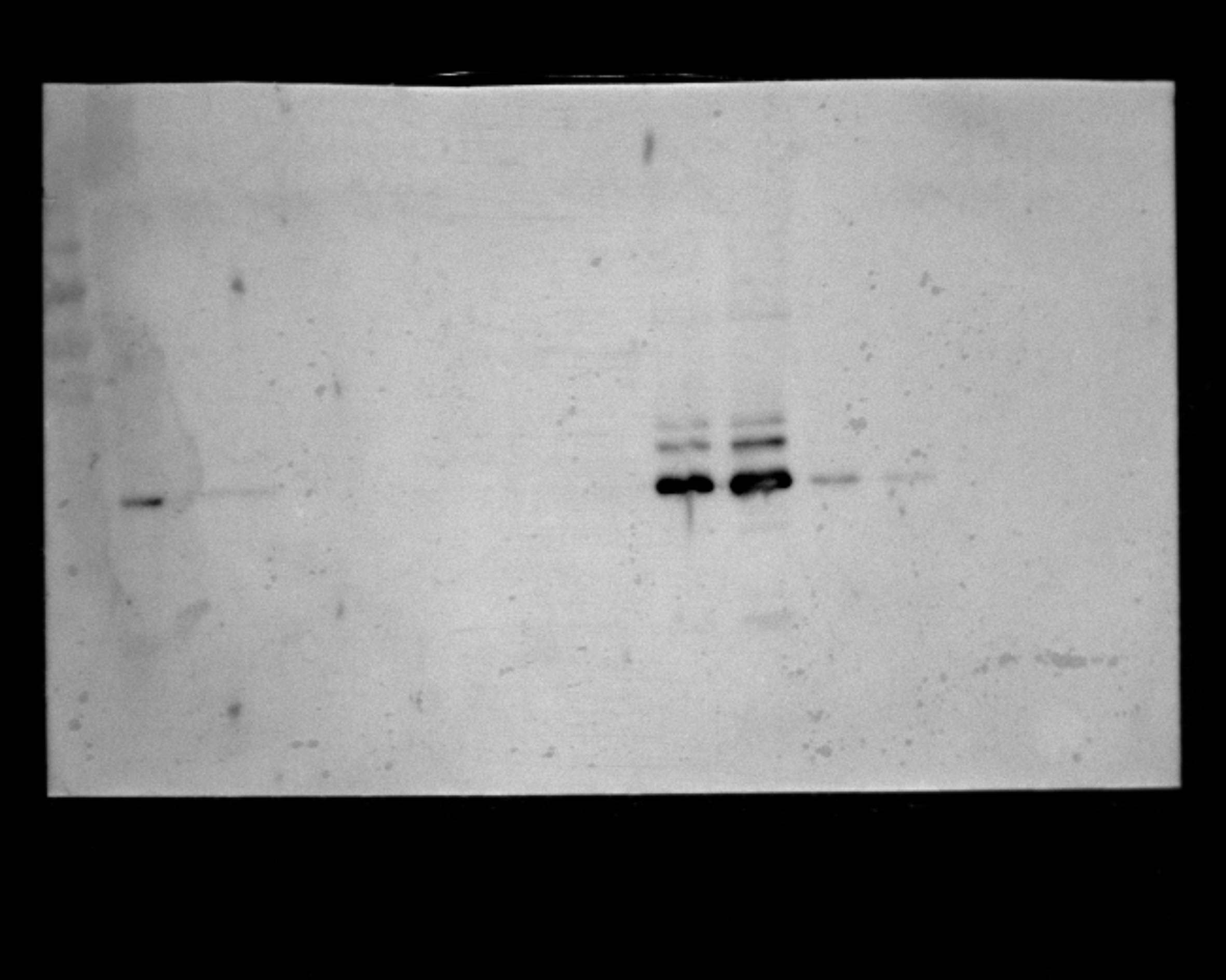

Supplement: Figure 2—figure supplement 1—source data 6. [file elife-101673-fig2-figsupp1-data6.zip › tsg101 raw unedited.tiff]

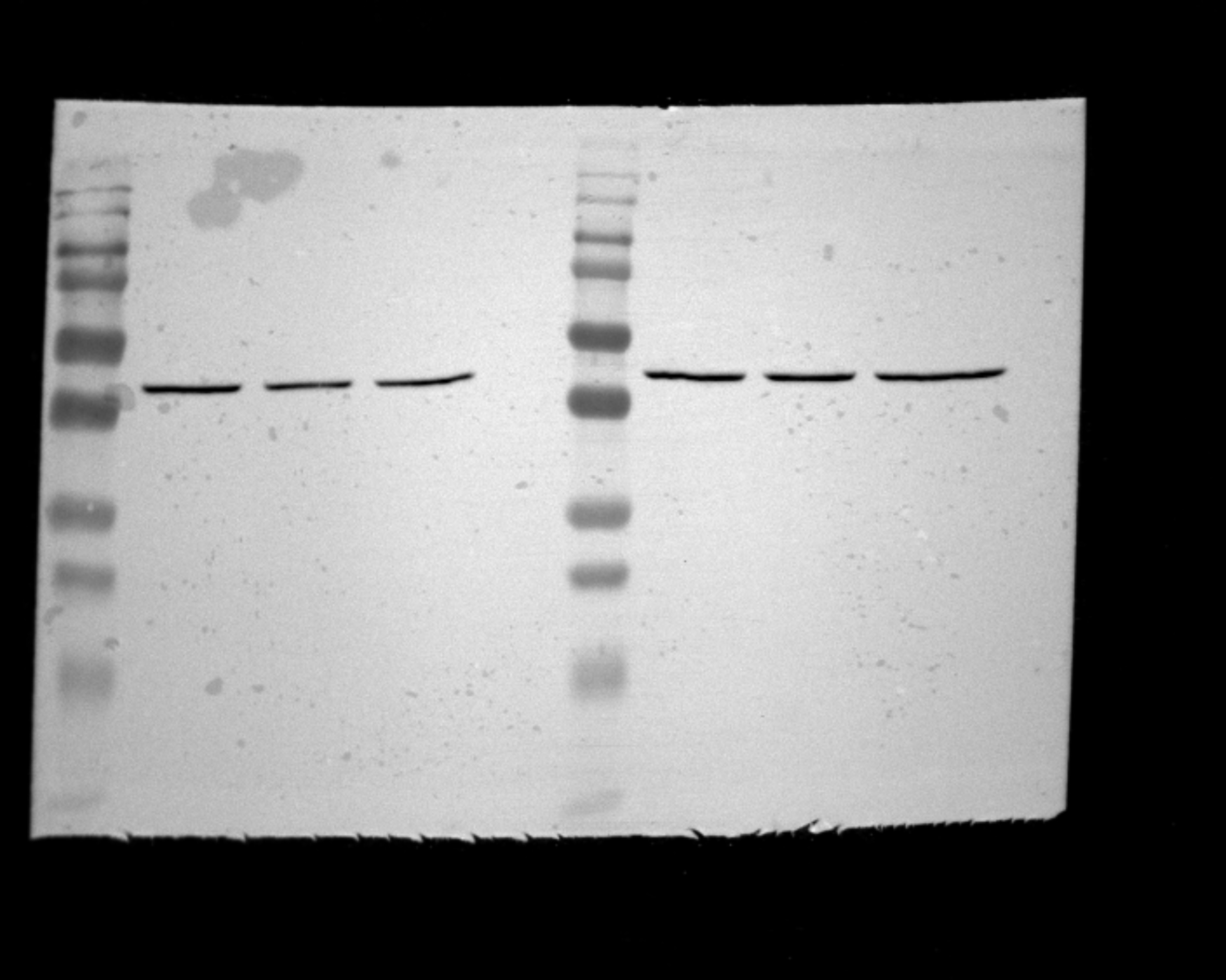

Supplement: Figure 2—figure supplement 2—source data 2. [file elife-101673-fig2-figsupp2-data2.zip › actin raw unedited.tiff]

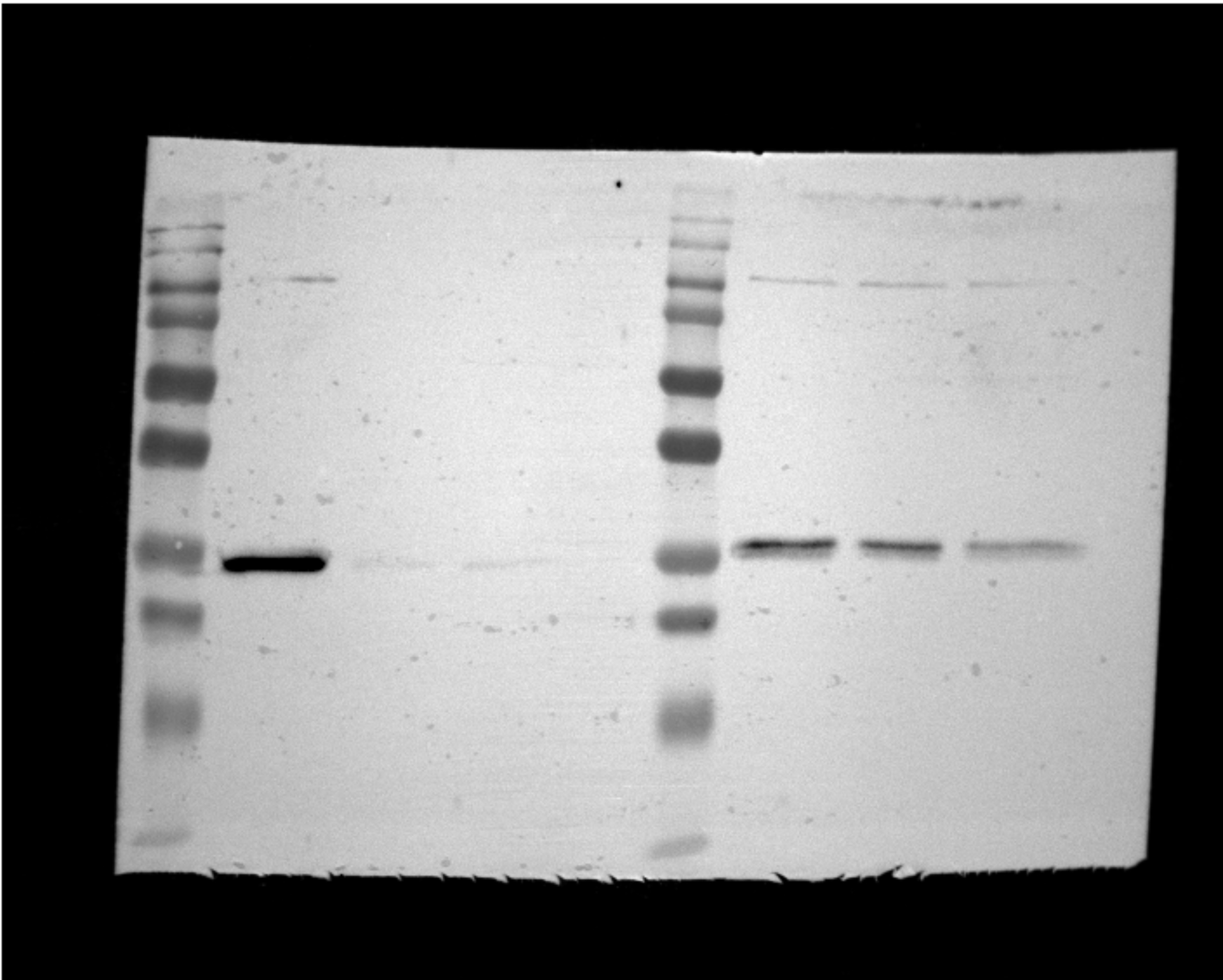

Supplement: Figure 2—figure supplement 2—source data 2. [file elife-101673-fig2-figsupp2-data2.zip › rab27a raw unedited.tiff]

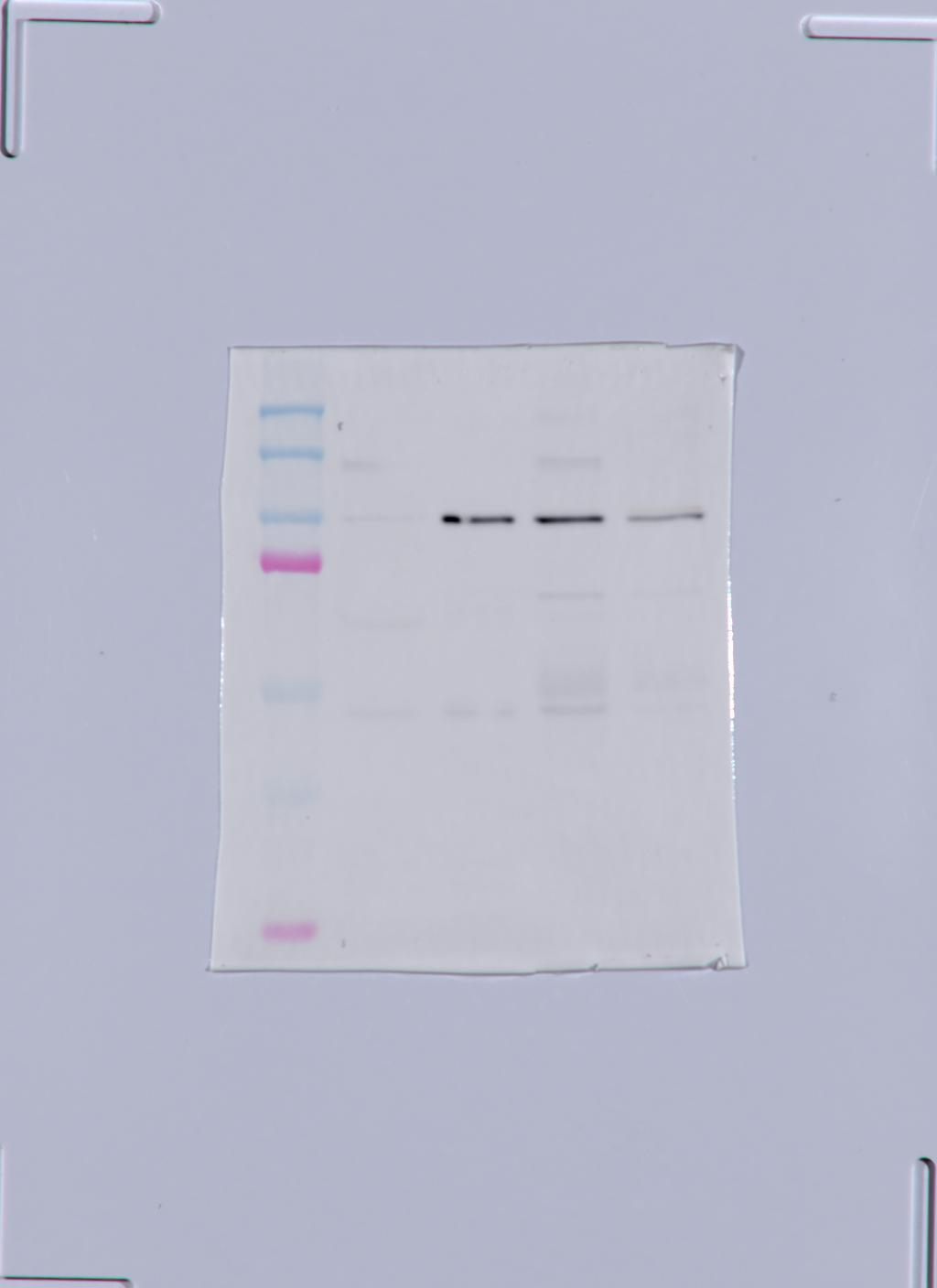

Supplement: Figure 3—figure supplement 2—source data 2. [file elife-101673-fig3-figsupp2-data2.zip › alix raw unedited.tiff]

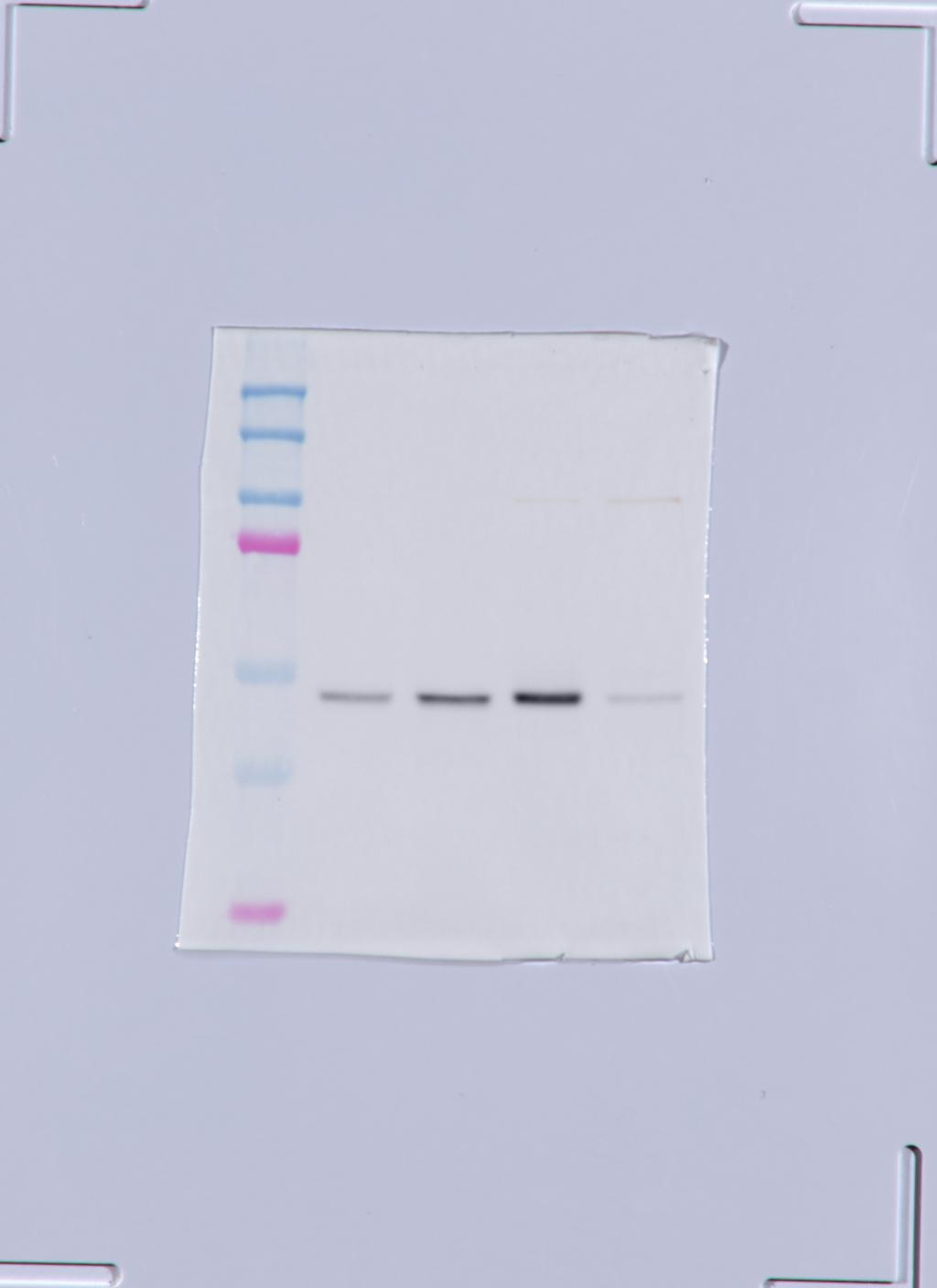

Supplement: Figure 3—figure supplement 2—source data 2. [file elife-101673-fig3-figsupp2-data2.zip › flot1 raw unedited.tiff]

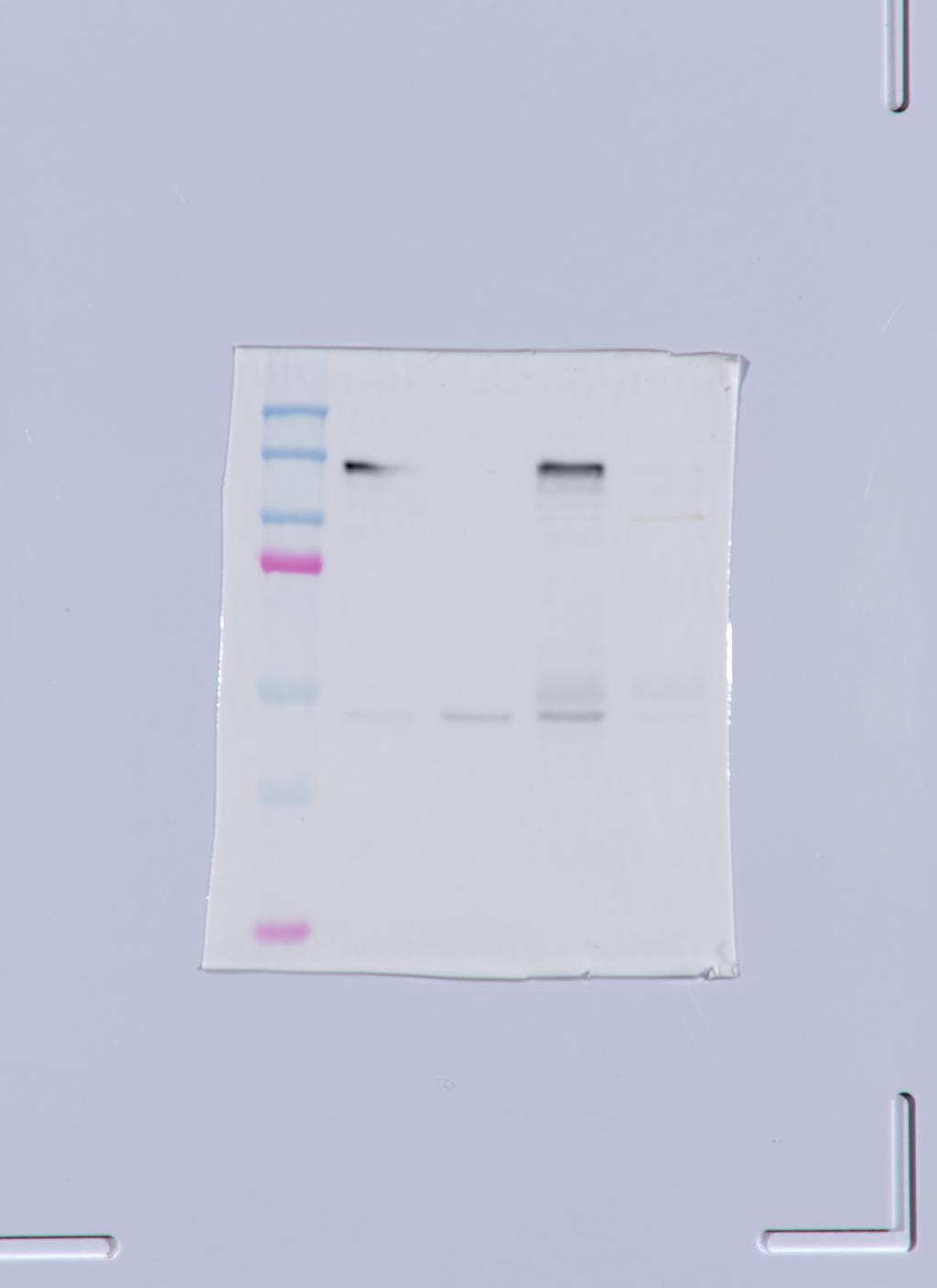

Supplement: Figure 3—figure supplement 2—source data 2. [file elife-101673-fig3-figsupp2-data2.zip › gm130 raw unedited.tiff]

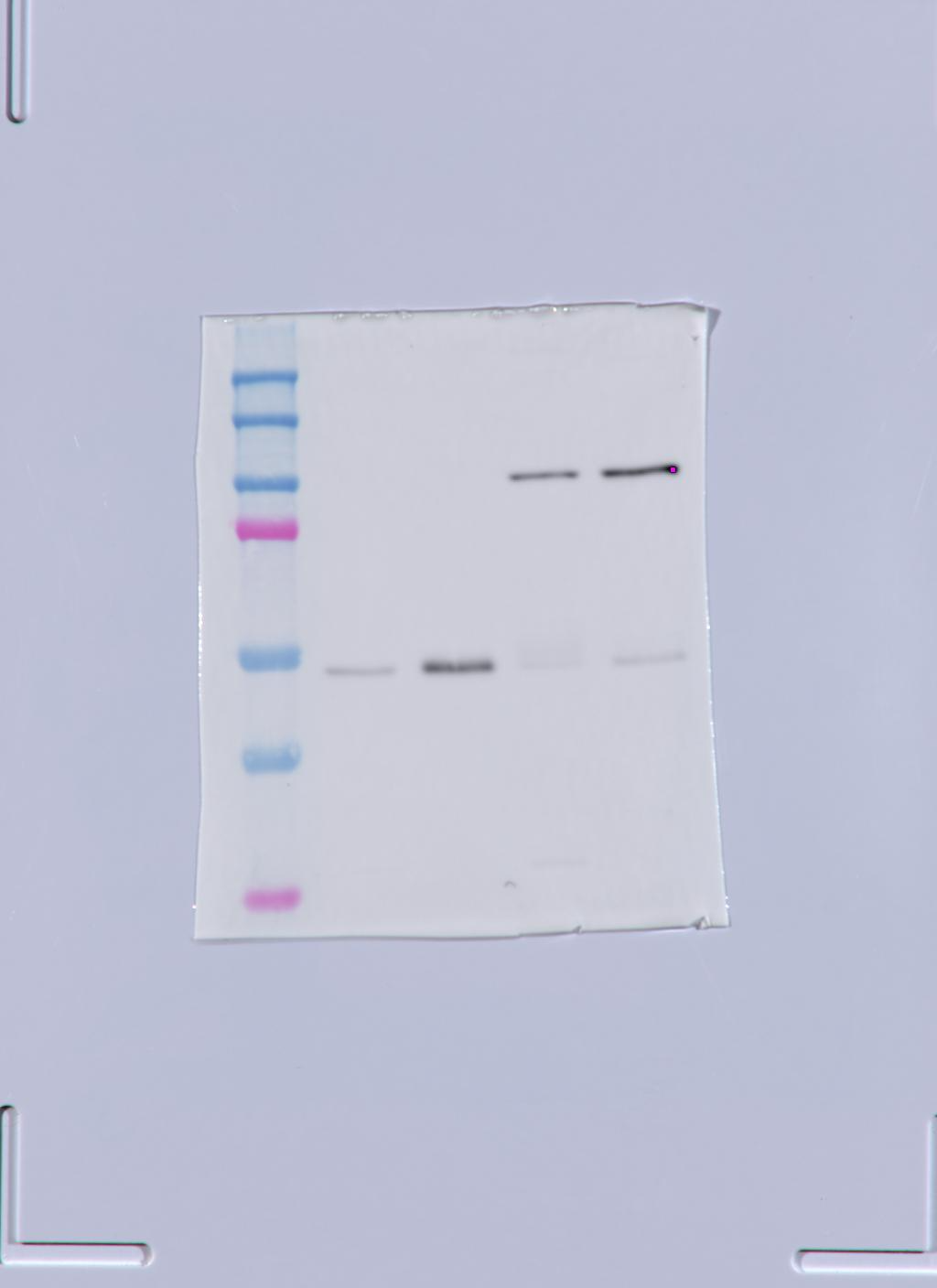

Supplement: Figure 3—figure supplement 2—source data 2. [file elife-101673-fig3-figsupp2-data2.zip › tsg101 raw unedited.tiff]

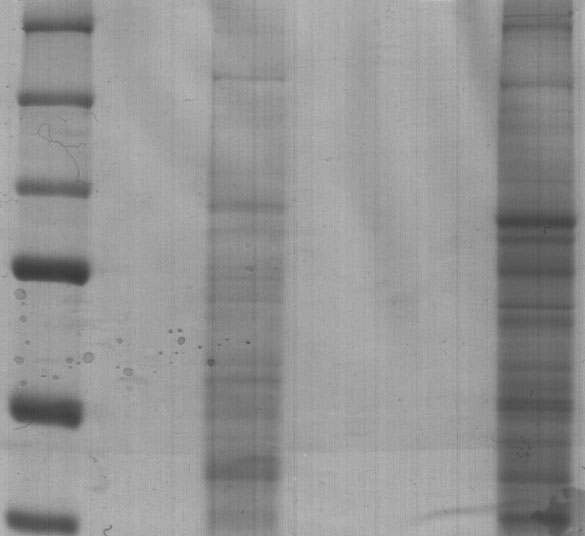

Supplement: Figure 4—source data 2. [file elife-101673-fig4-data2.zip › Figure 4_Source Data 2.tif]

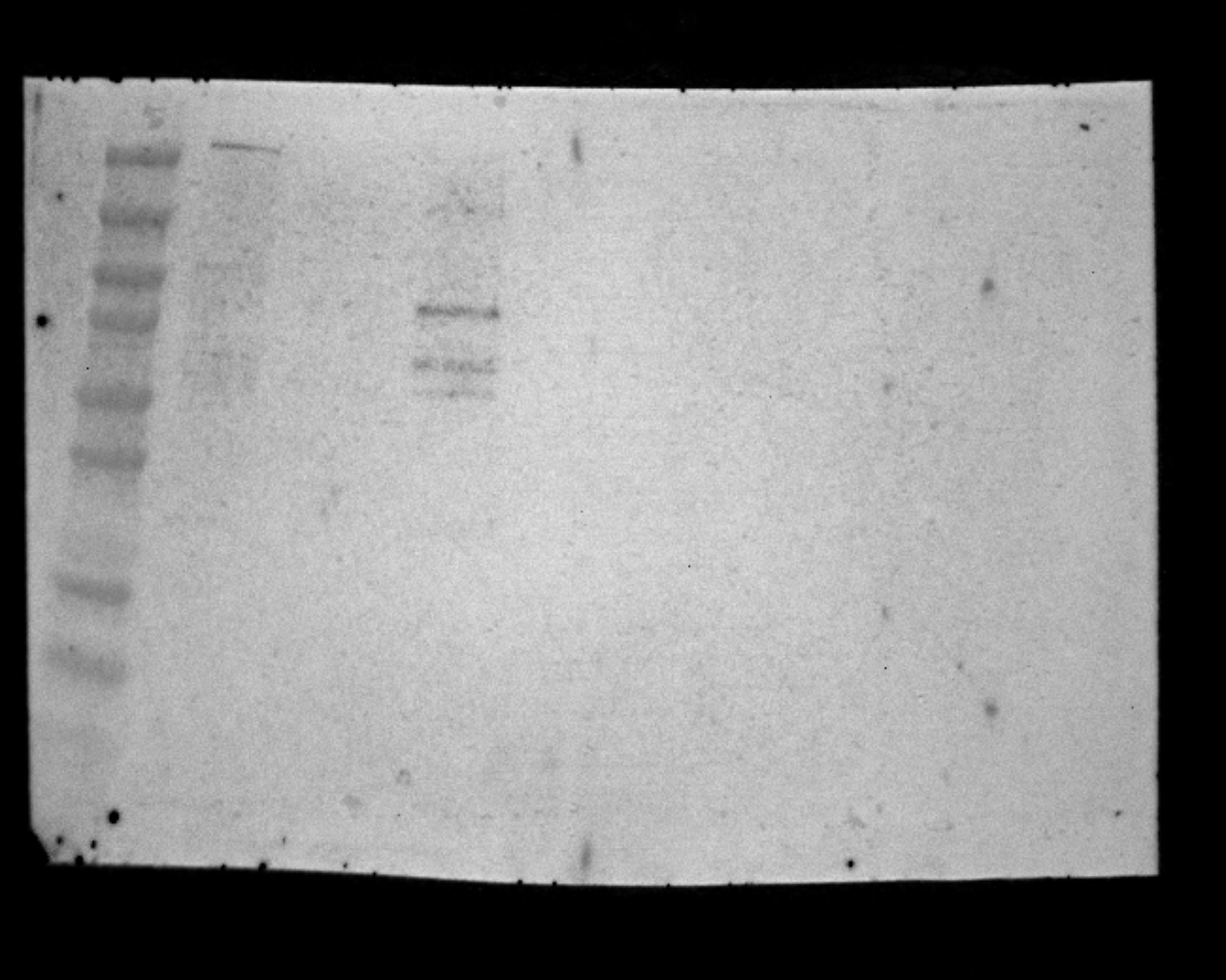

Supplement: Figure 4—source data 4. [file elife-101673-fig4-data4.zip › 4B endoglin raw unedited.tiff]

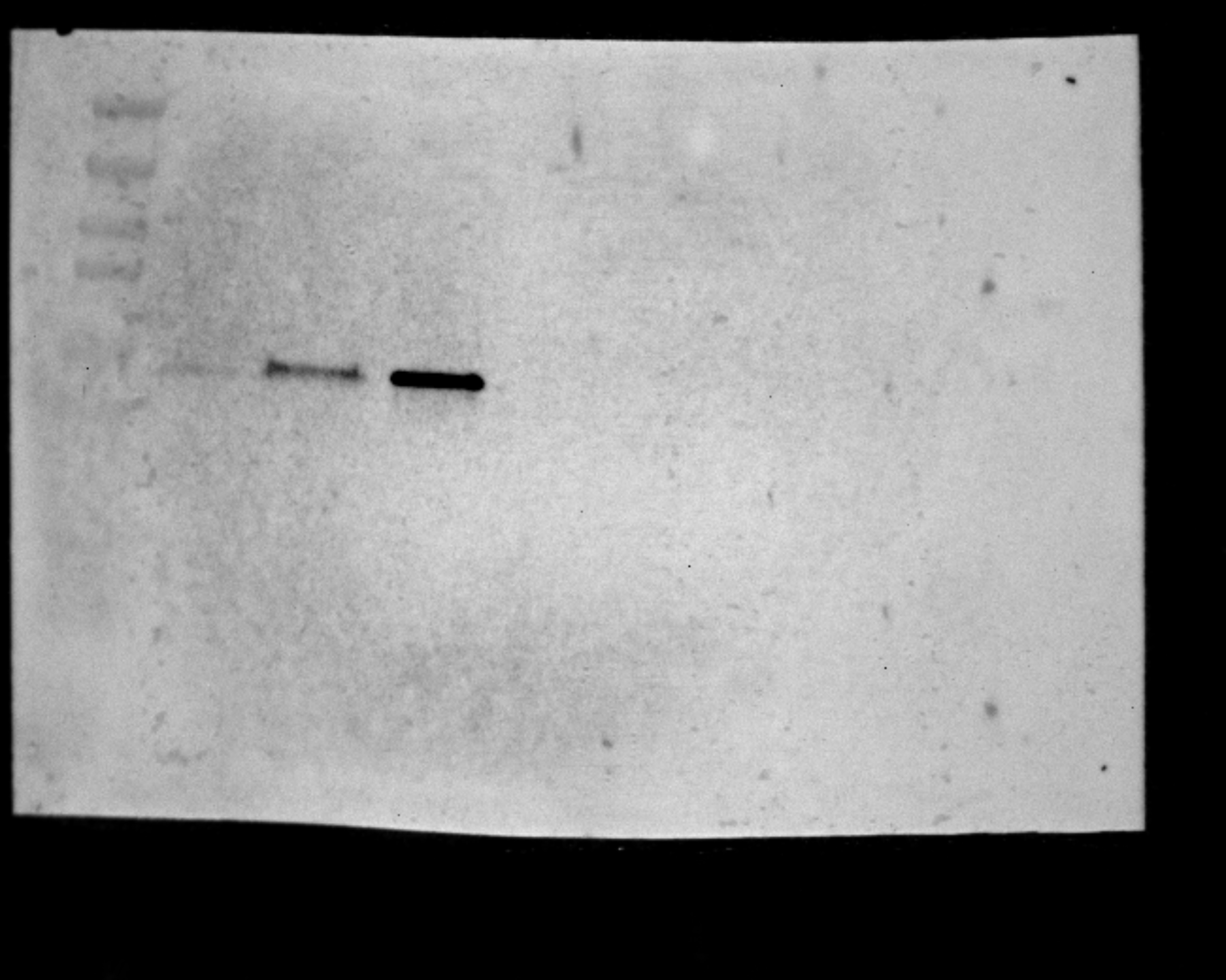

Supplement: Figure 4—source data 4. [file elife-101673-fig4-data4.zip › 4B flot-1 raw unedited.tiff]

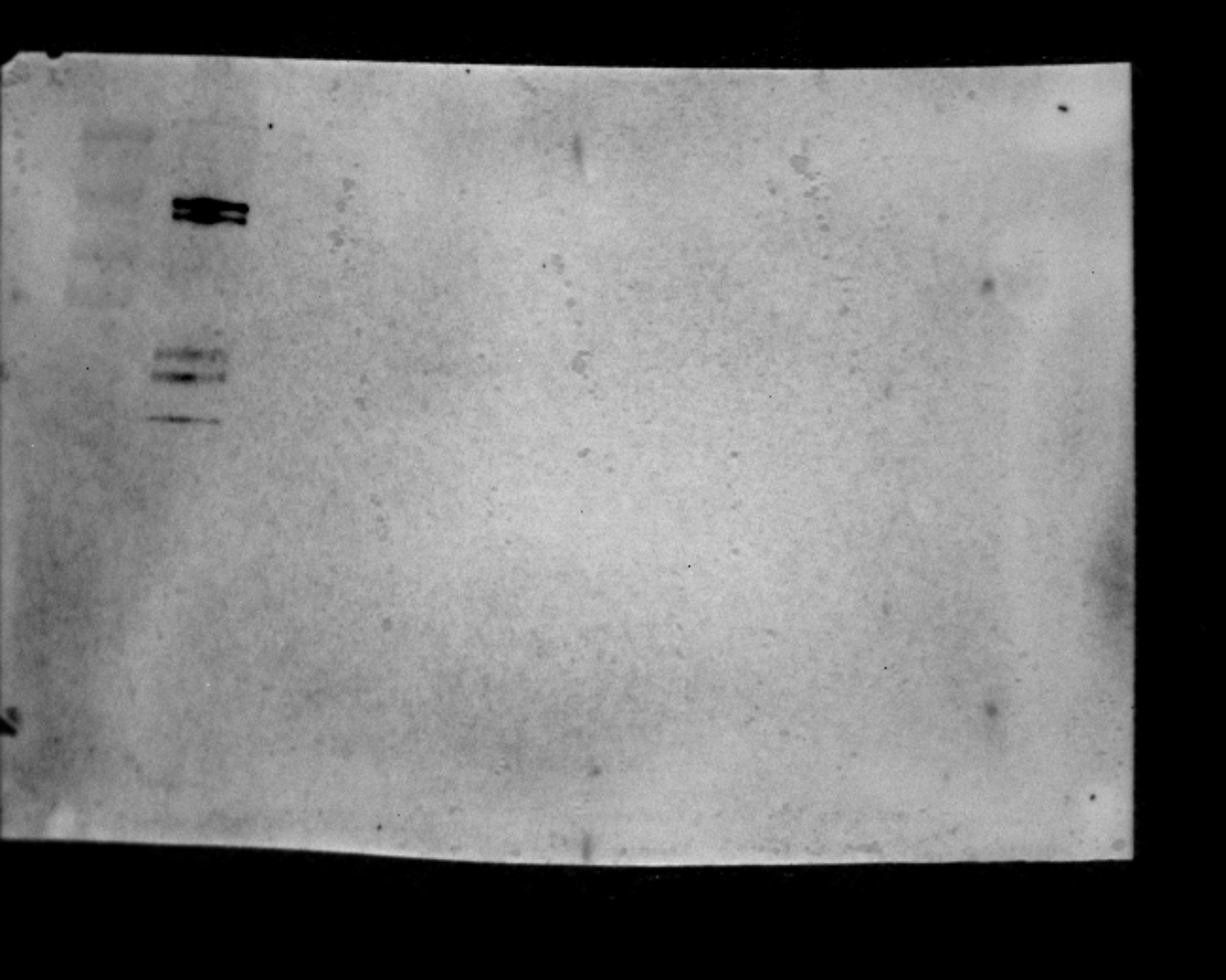

Supplement: Figure 4—source data 4. [file elife-101673-fig4-data4.zip › 4B gm130 raw unedited.tiff]

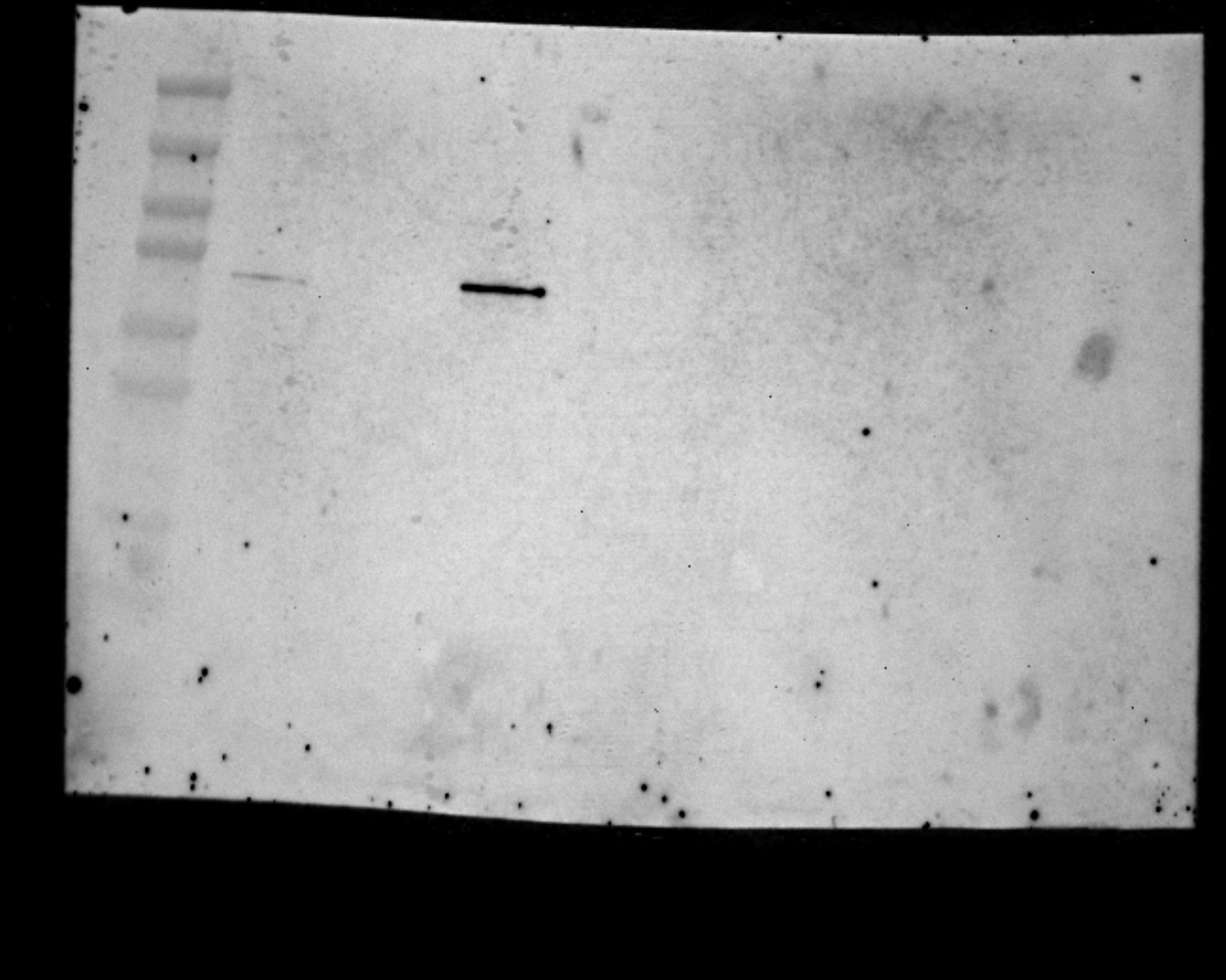

Supplement: Figure 4—source data 4. [file elife-101673-fig4-data4.zip › 4B HSP70 raw unedited.tiff]

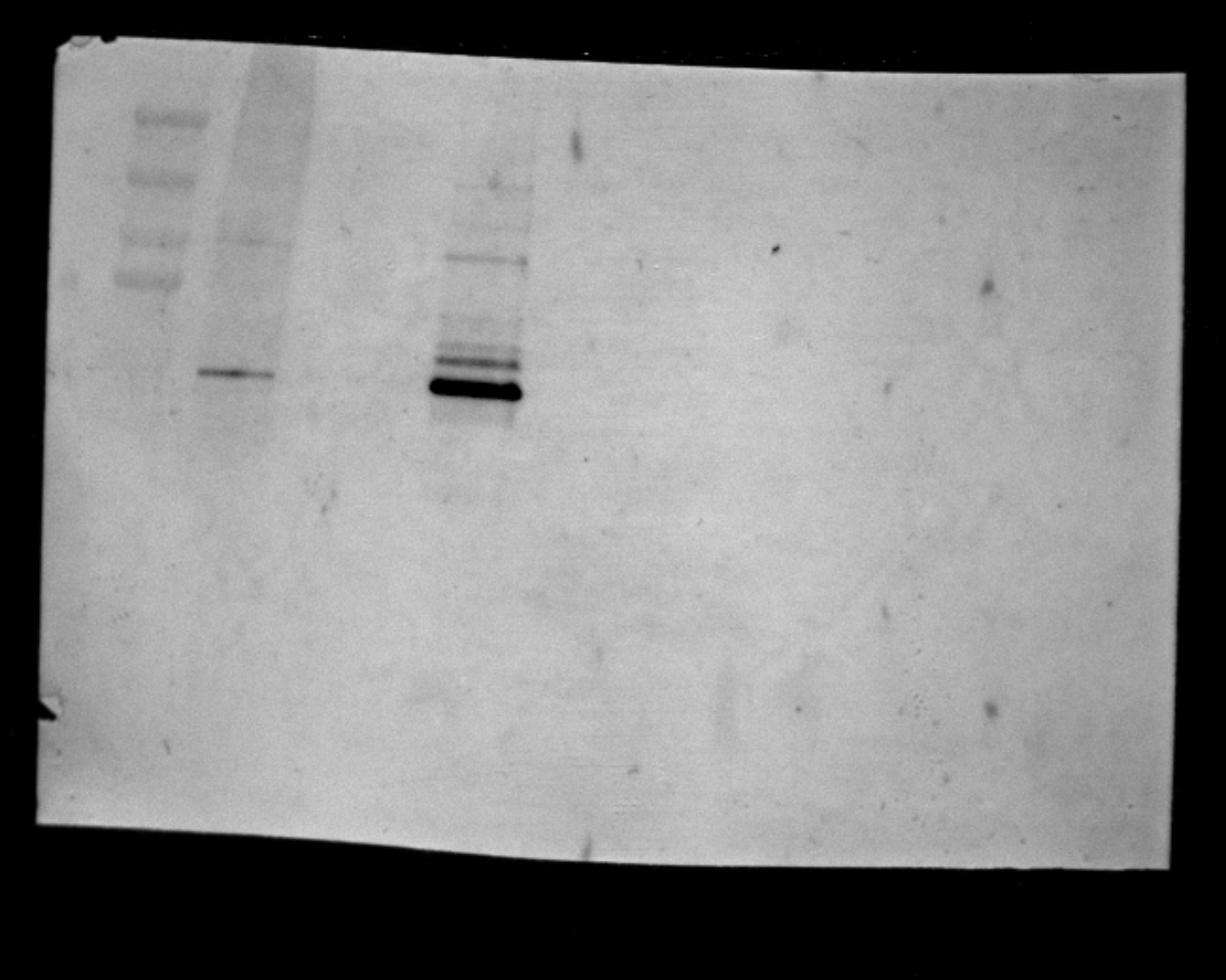

Supplement: Figure 4—source data 4. [file elife-101673-fig4-data4.zip › 4B TSG101 raw unedited.tiff]

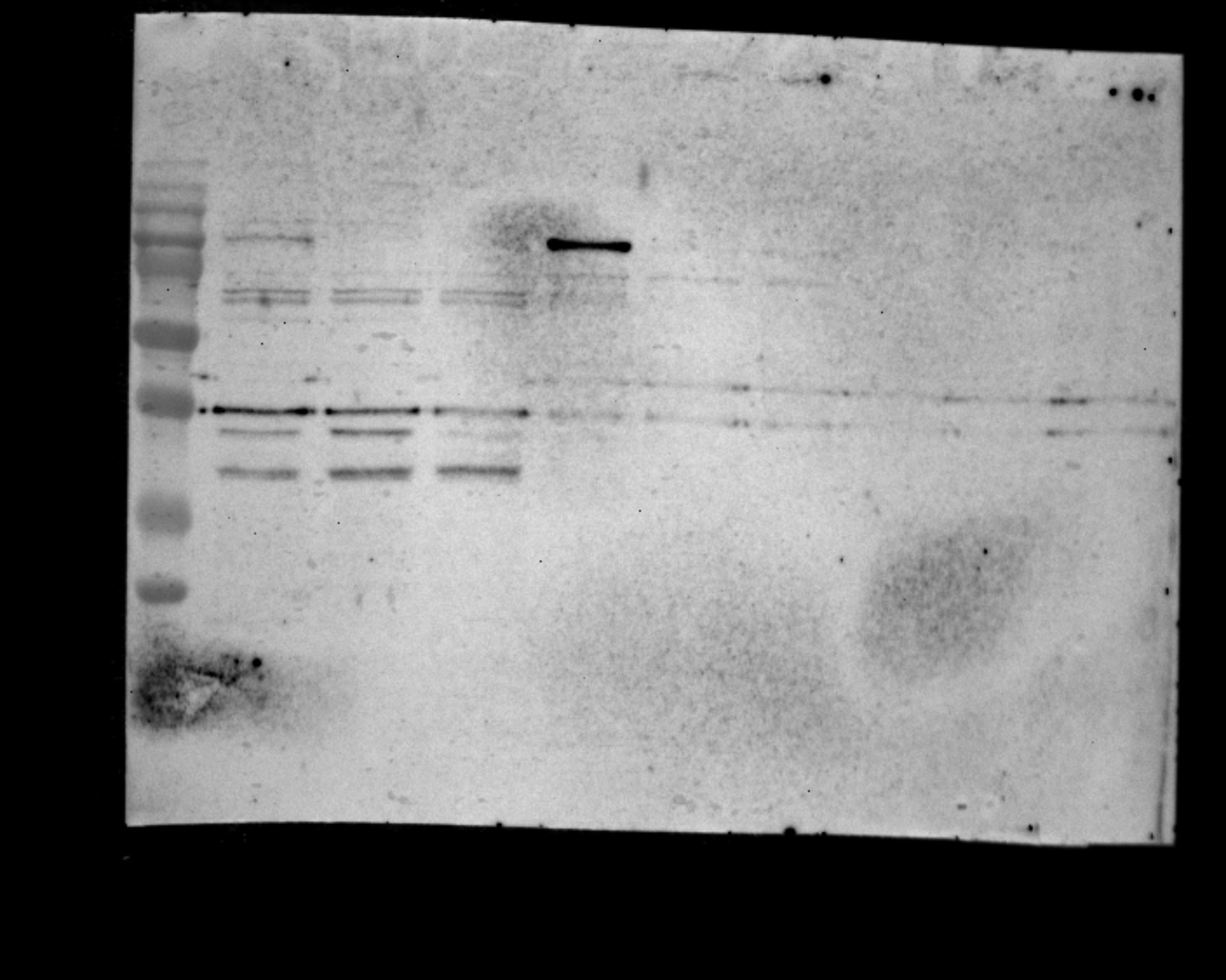

Supplement: Figure 4—source data 6. [file elife-101673-fig4-data6.zip › 4C endoglin raw unedited.tiff]

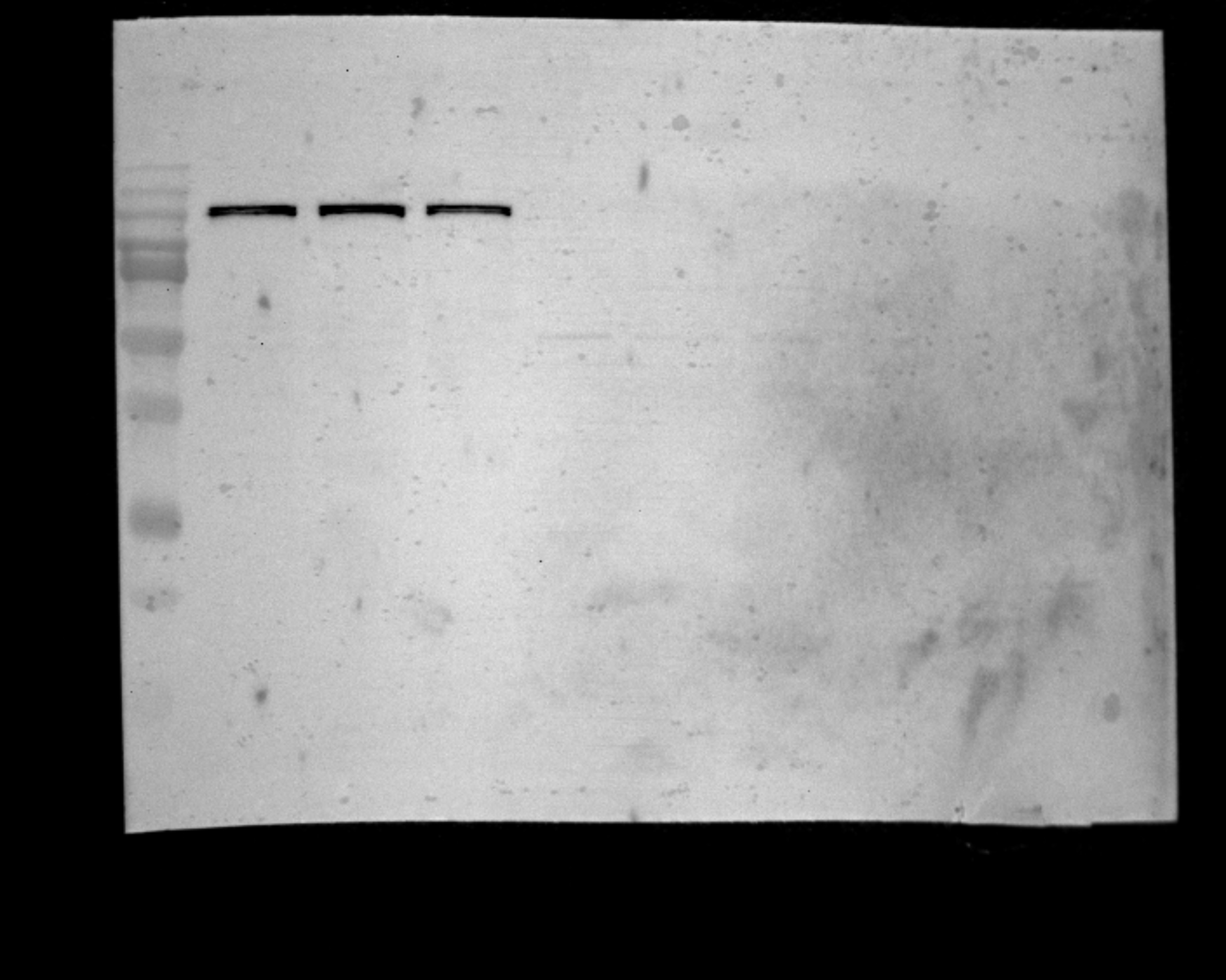

Supplement: Figure 4—source data 6. [file elife-101673-fig4-data6.zip › 4C gm130 raw unedited.tiff]

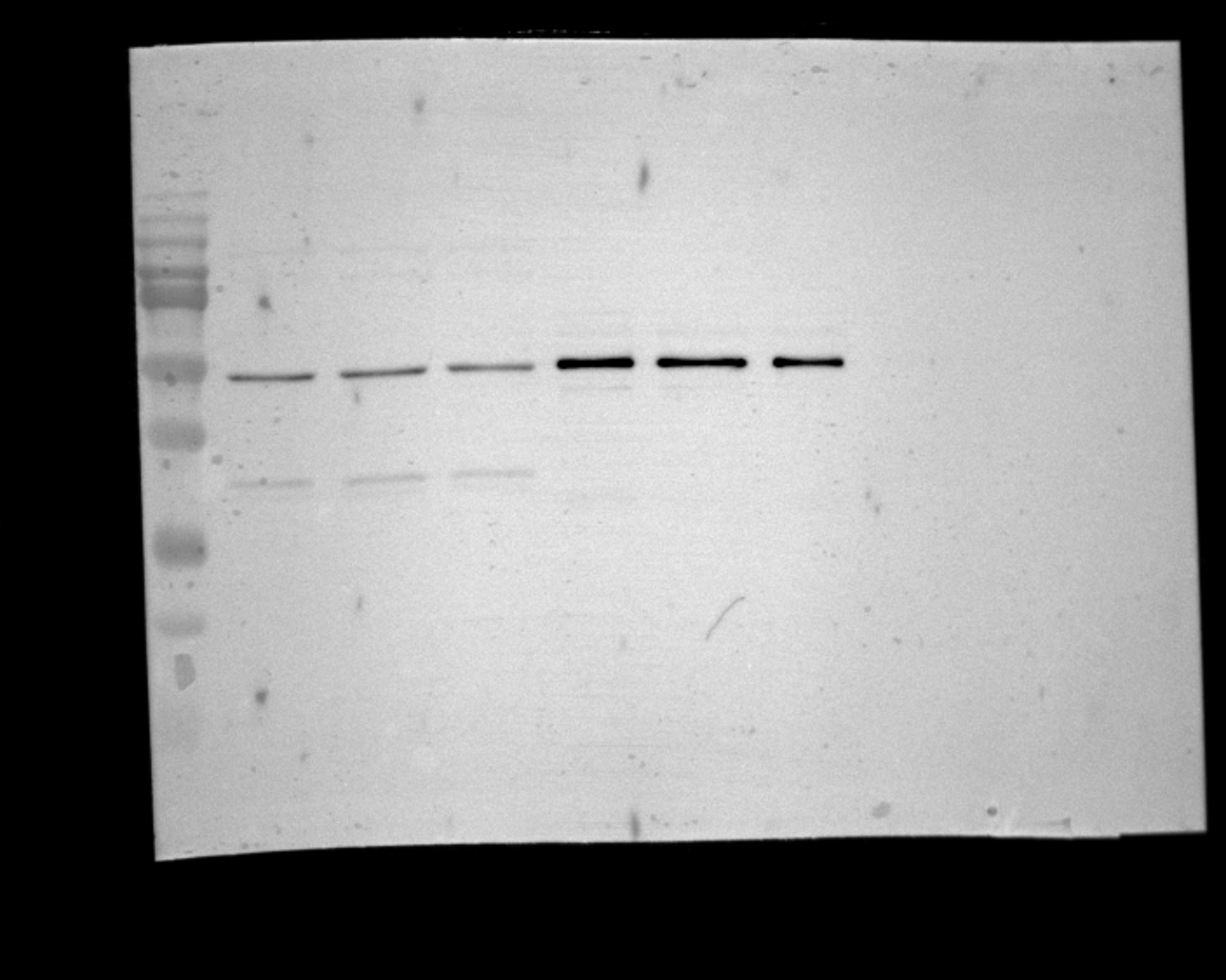

Supplement: Figure 4—source data 6. [file elife-101673-fig4-data6.zip › 4C tsg101 raw unedited.tiff]

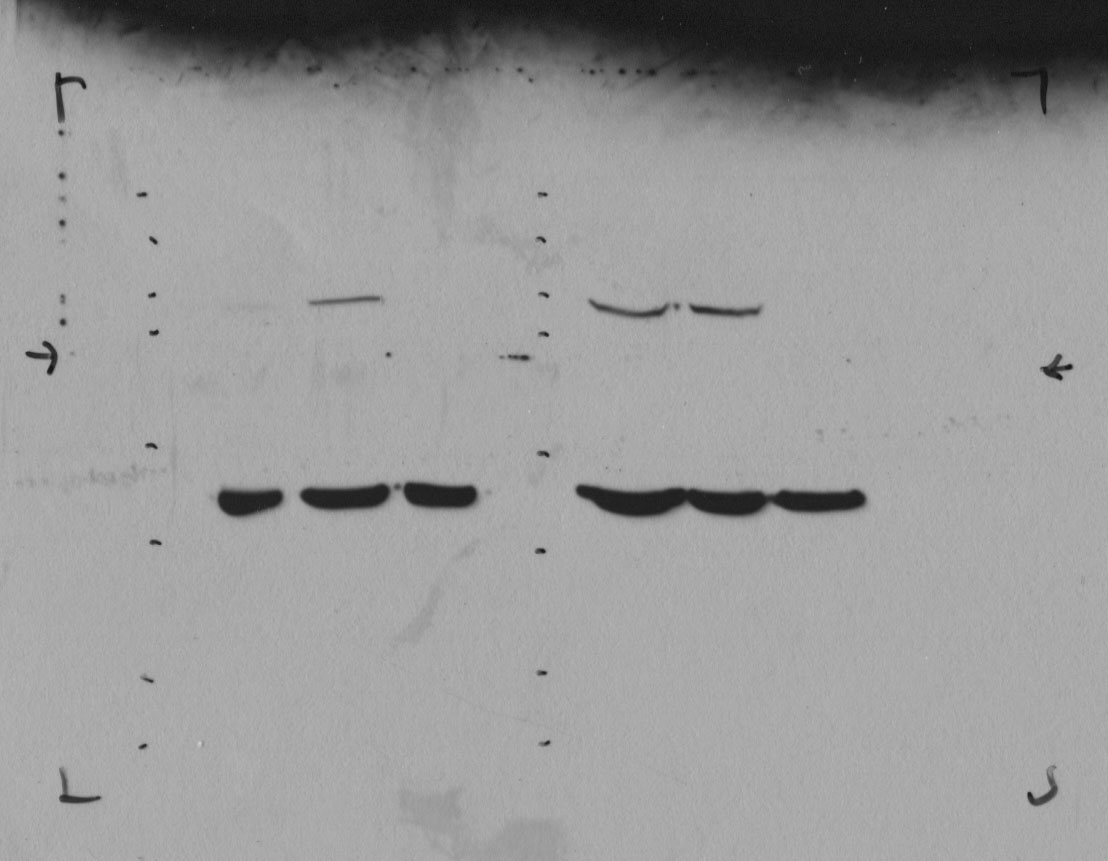

Supplement: Figure 4—figure supplement 1—source data 2. [file elife-101673-fig4-figsupp1-data2.zip › actin raw unedited.tiff]

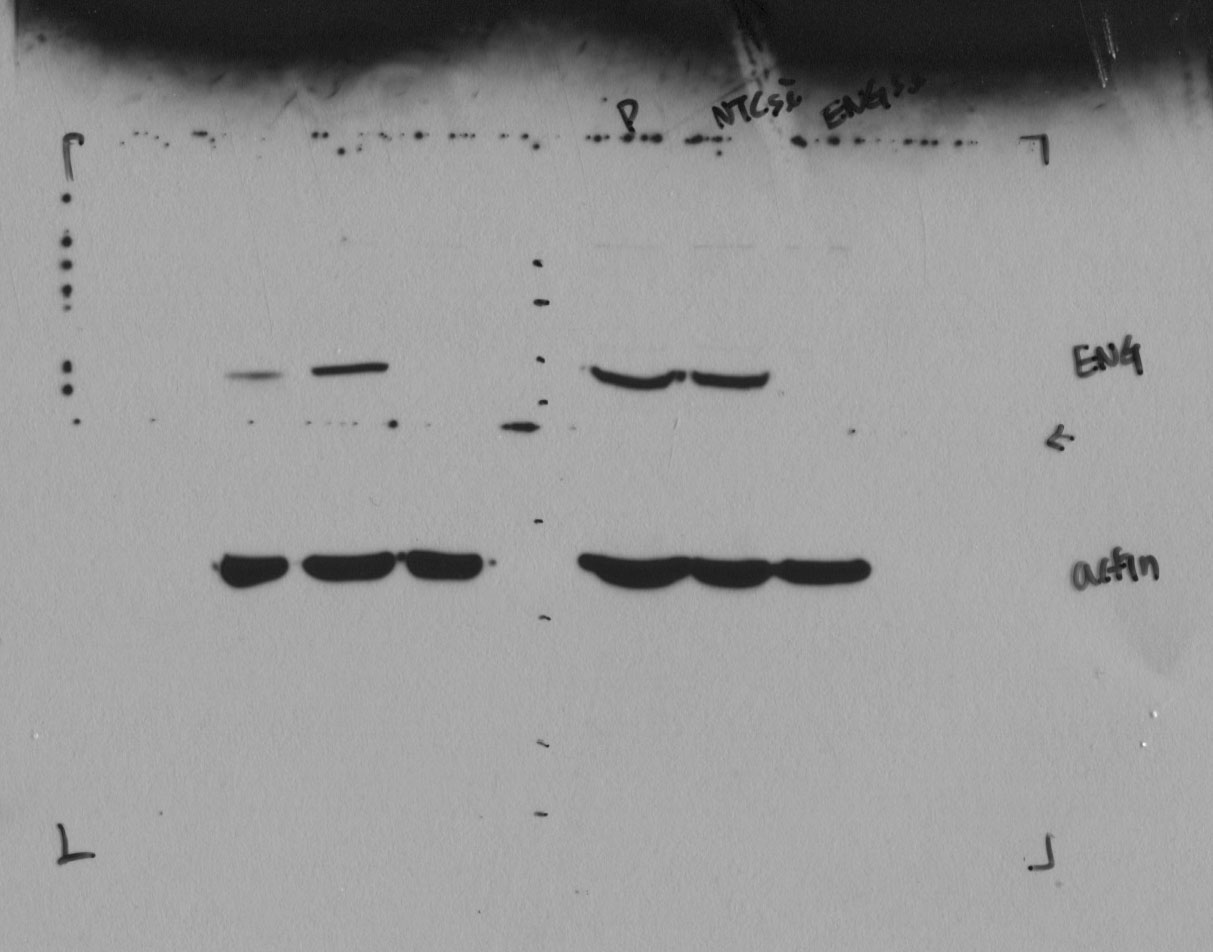

Supplement: Figure 4—figure supplement 1—source data 2. [file elife-101673-fig4-figsupp1-data2.zip › endoglin raw unedited.tiff]

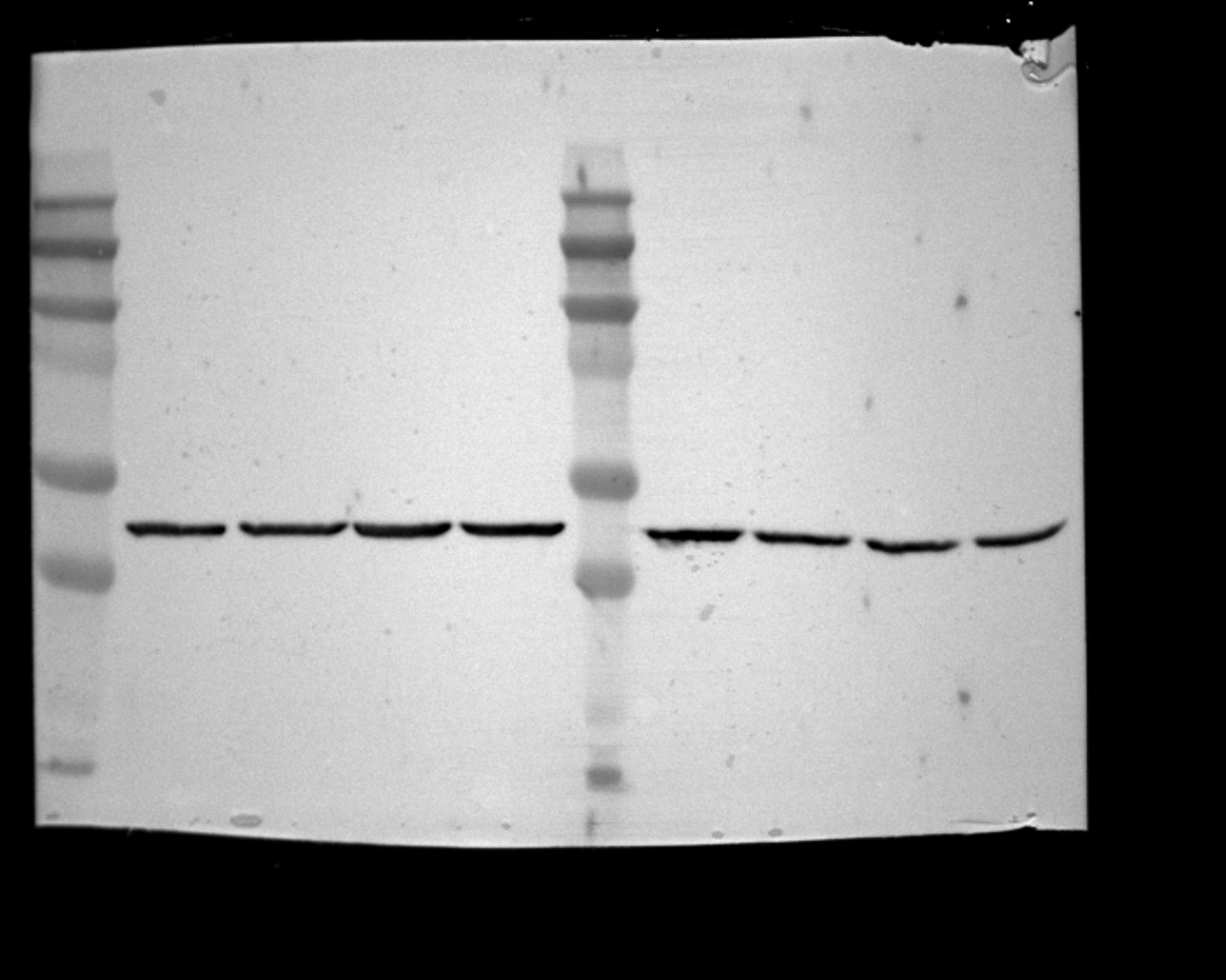

Supplement: Figure 4—figure supplement 2—source data 2. [file elife-101673-fig4-figsupp2-data2.zip › actin raw unedited.tiff]

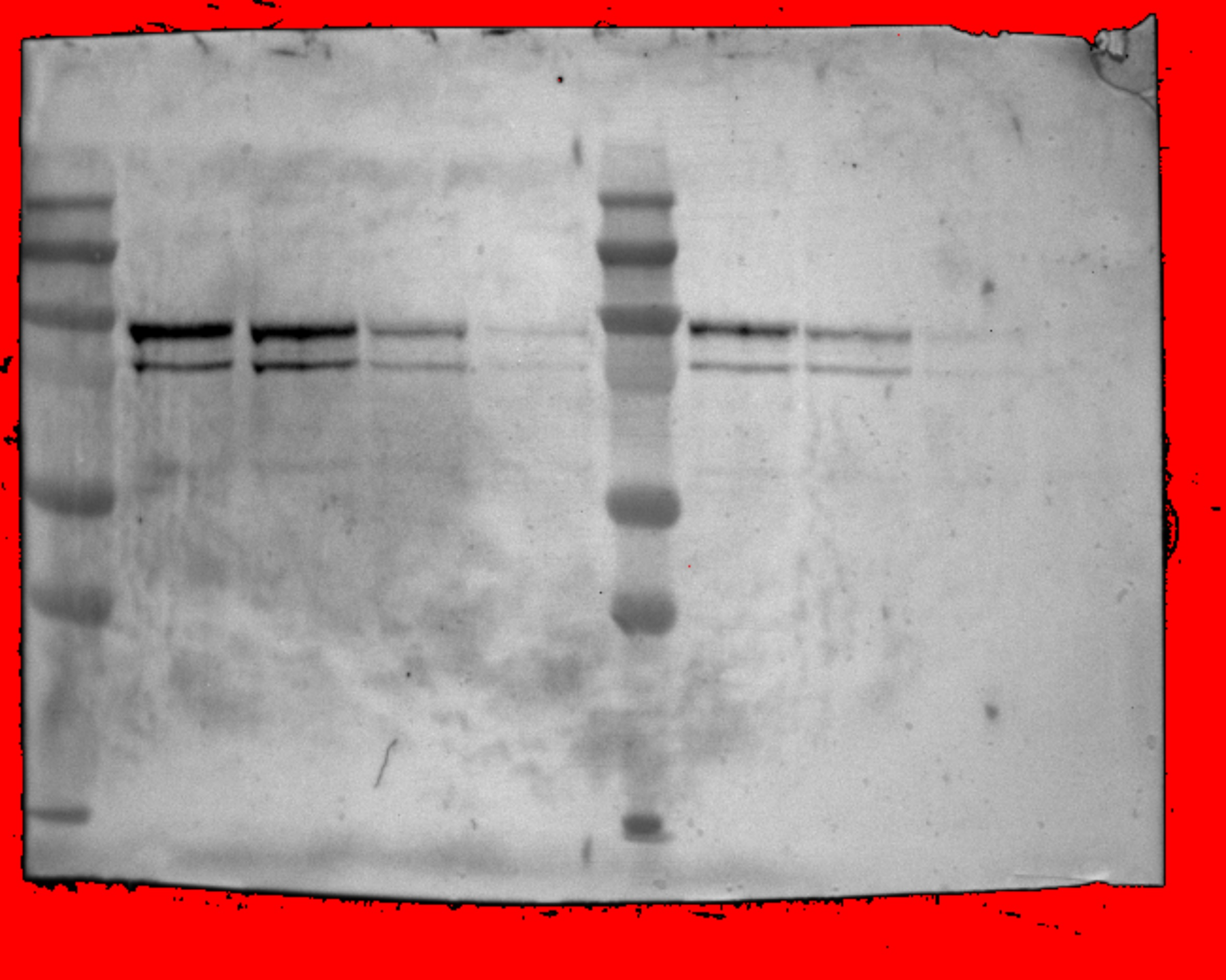

Supplement: Figure 4—figure supplement 2—source data 2. [file elife-101673-fig4-figsupp2-data2.zip › endoglin raw unedited.tiff]

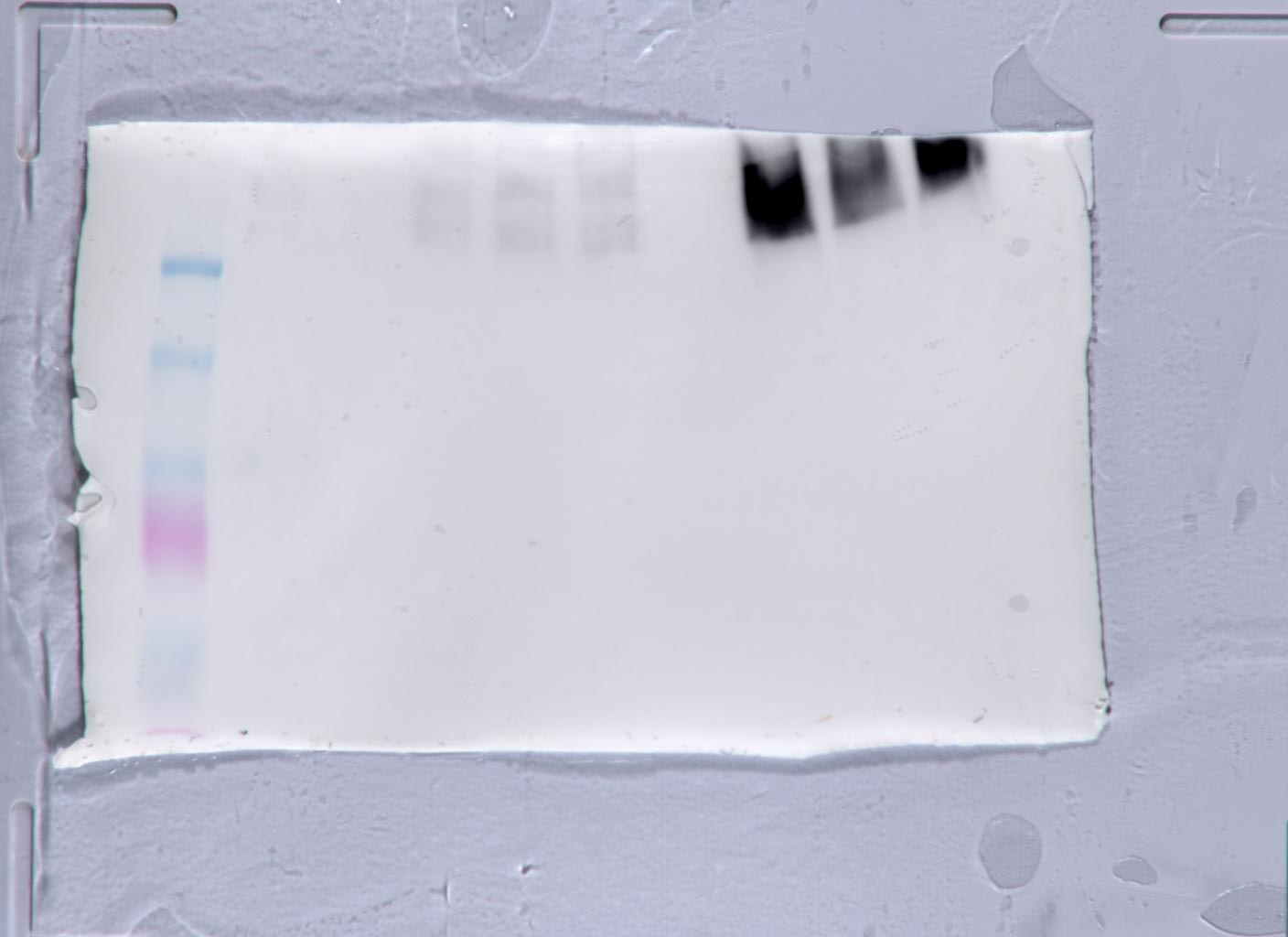

Supplement: Figure 6—source data 2. [file elife-101673-fig6-data2.zip › 6A cd63 raw unedited.tiff]

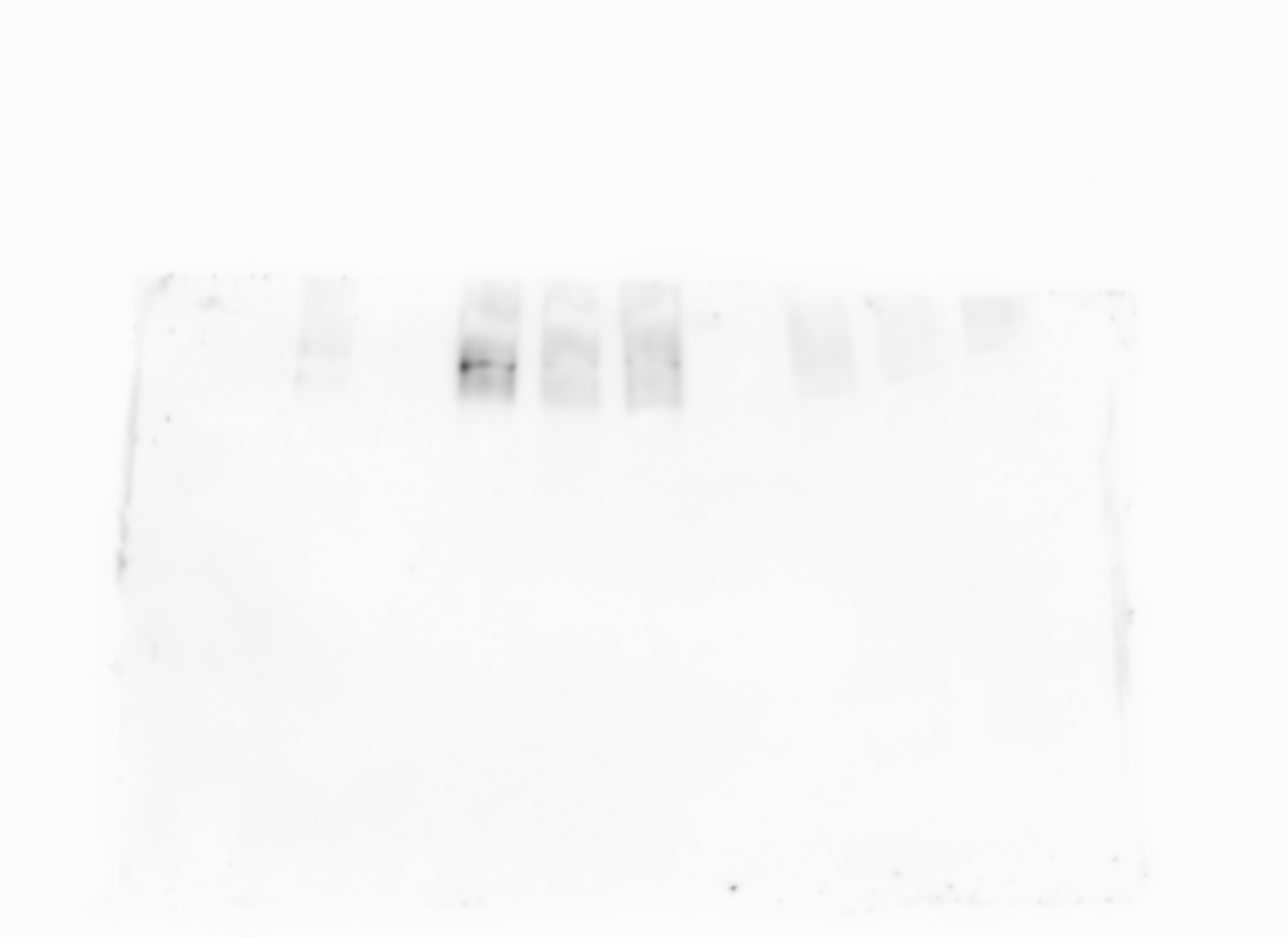

Supplement: Figure 6—source data 2. [file elife-101673-fig6-data2.zip › 6A endoglin raw unedited.tiff]

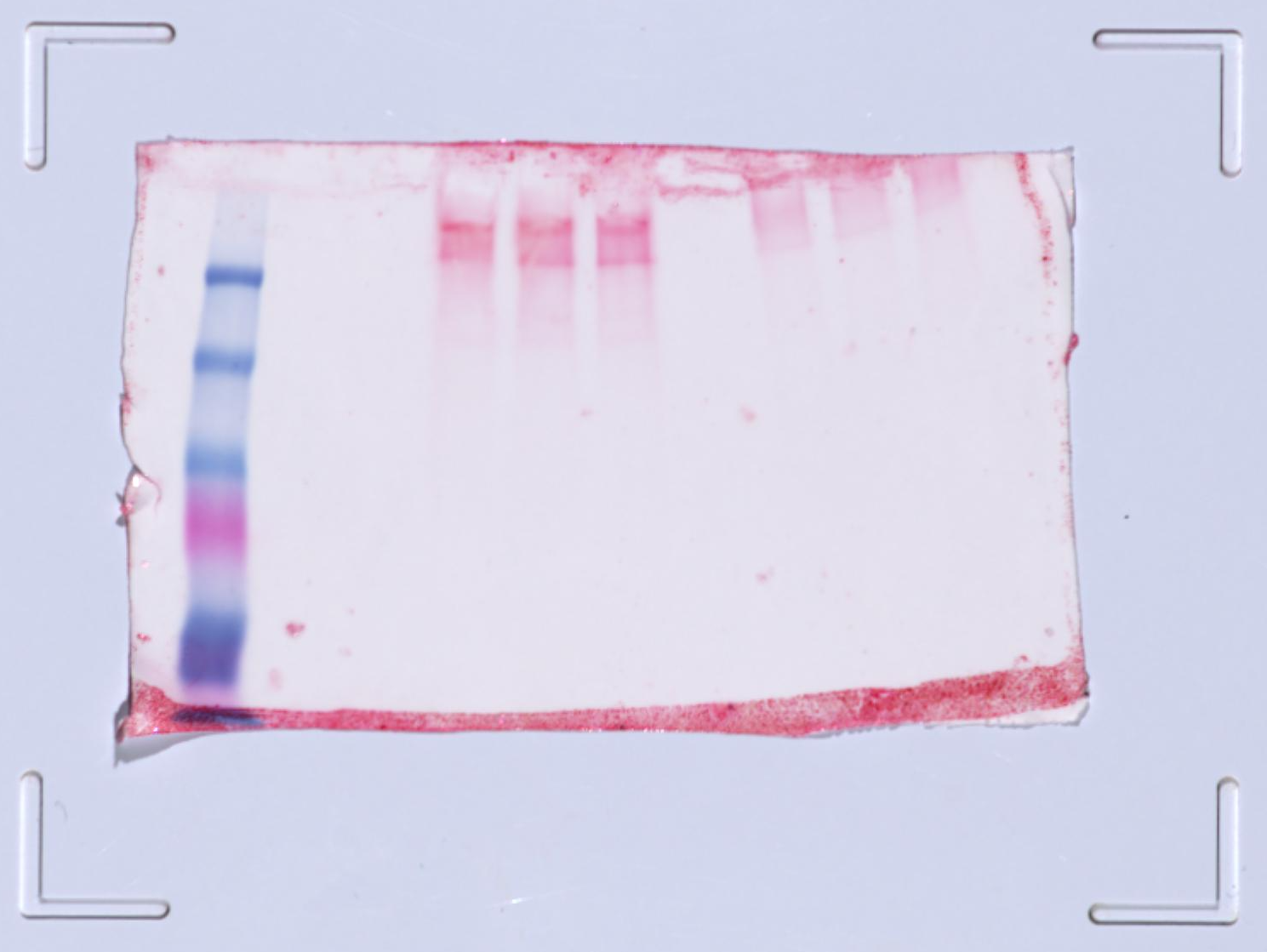

Supplement: Figure 6—source data 2. [file elife-101673-fig6-data2.zip › 6A ponceau raw unedited.tiff]

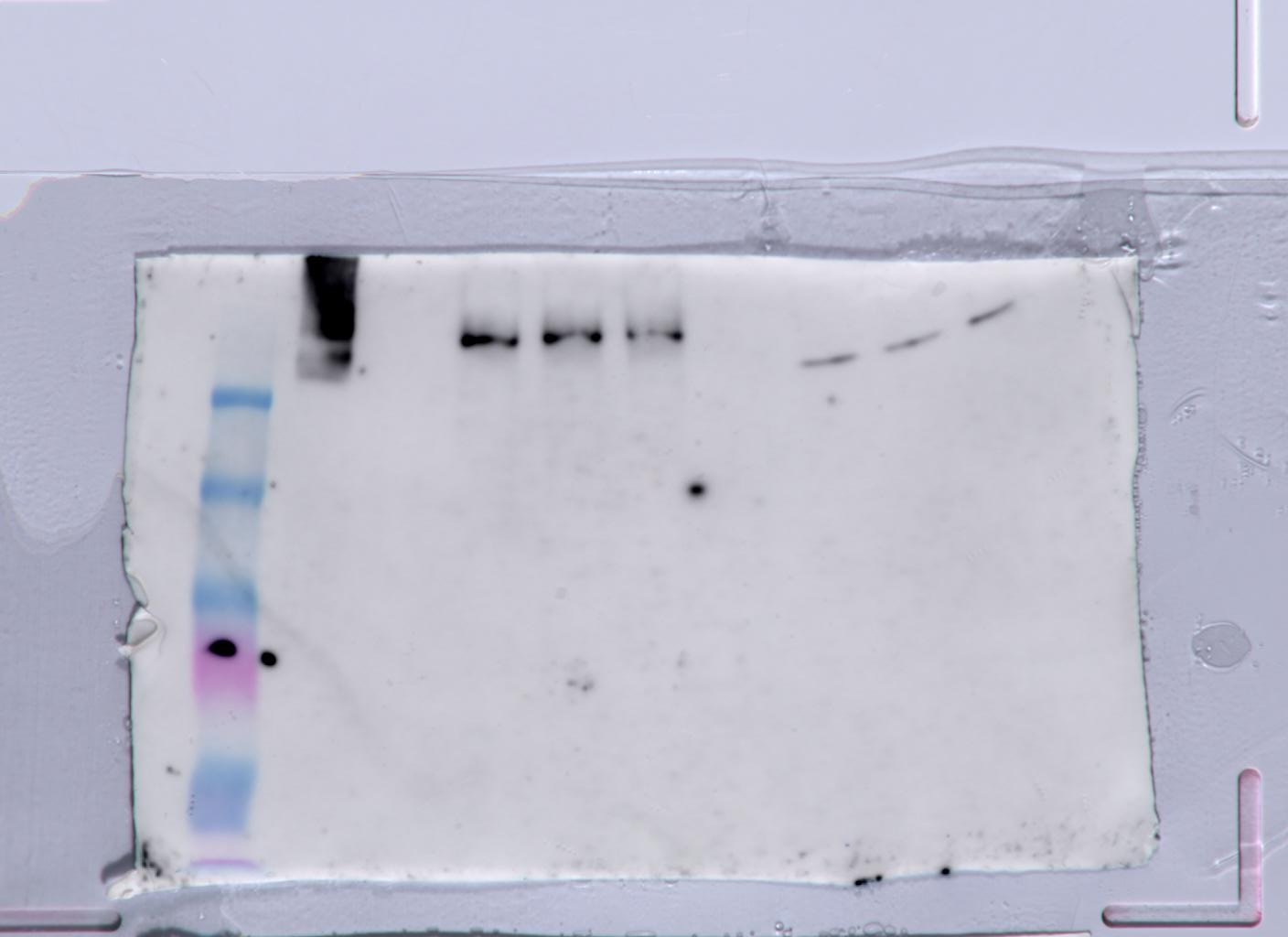

Supplement: Figure 6—source data 2. [file elife-101673-fig6-data2.zip › 6A thsd7a raw unedited.tiff]

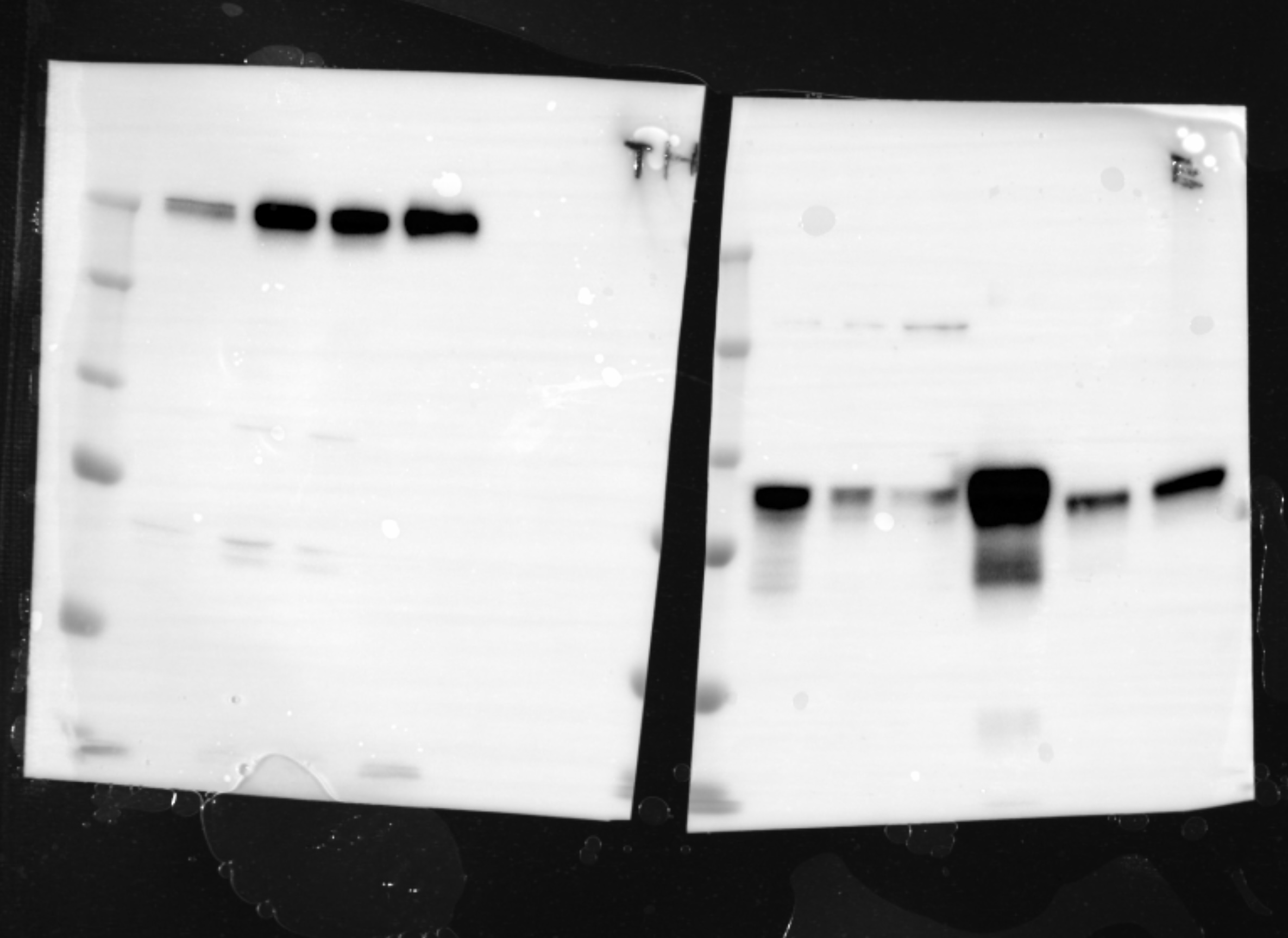

Supplement: Figure 6—source data 4. [file elife-101673-fig6-data4.zip › 6B thsd7a-left blot and endoglin-right blot raw unedited.tiff]

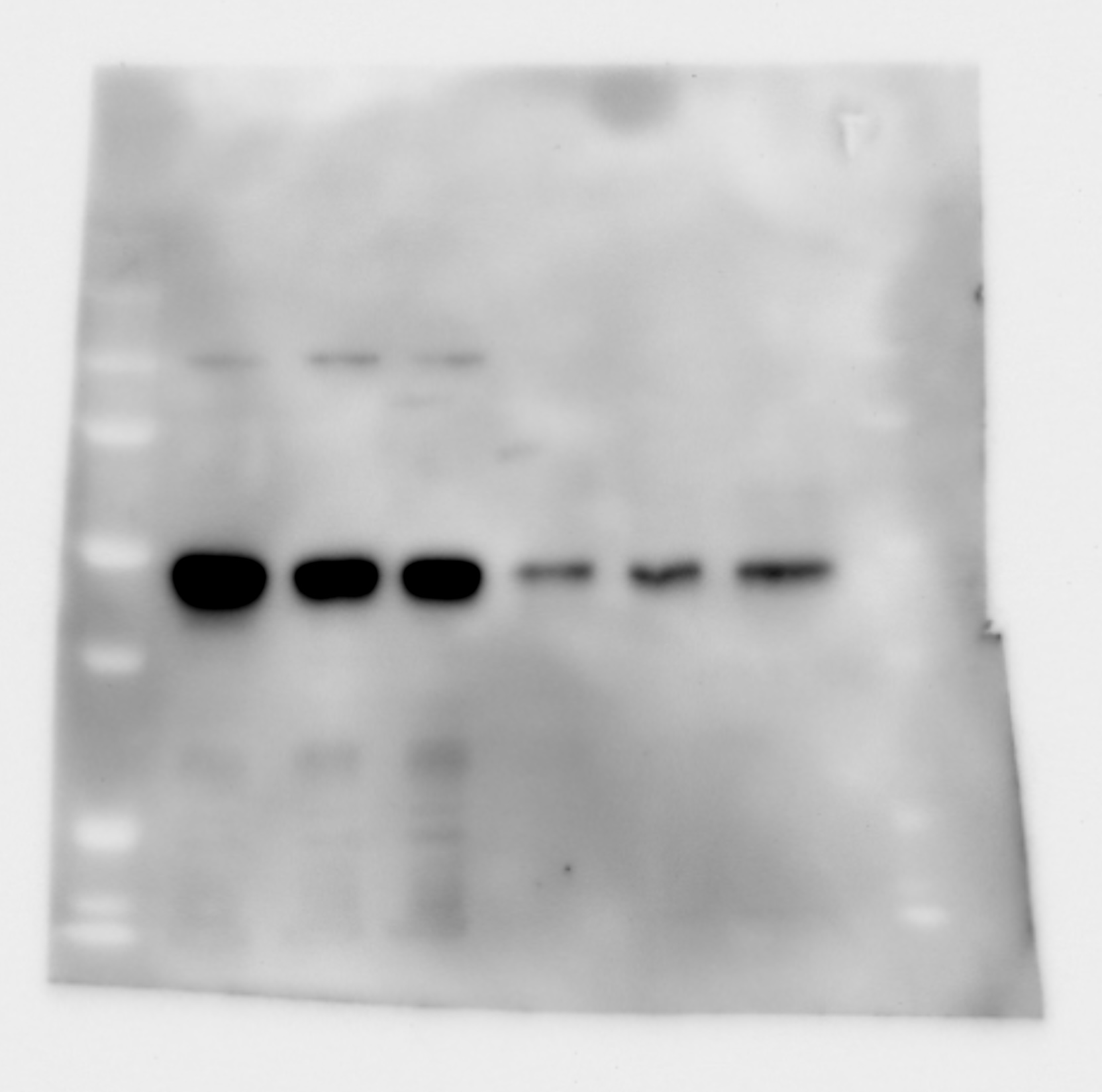

Supplement: Figure 6—source data 4. [file elife-101673-fig6-data4.zip › 6B tsg101 raw unedited.tiff]

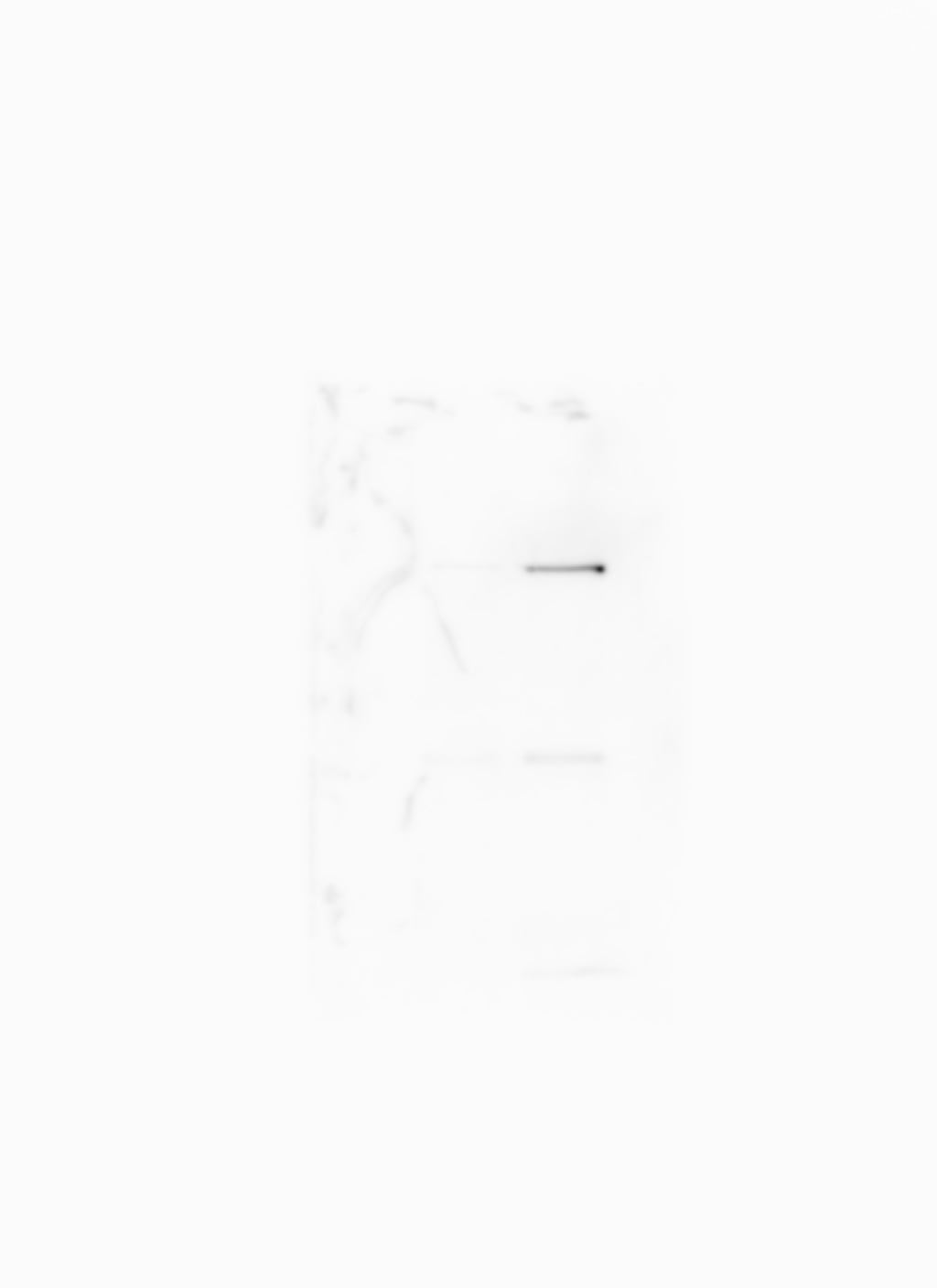

Supplement: Figure 6—source data 6. [file elife-101673-fig6-data6.zip › alix raw unedited.tiff]

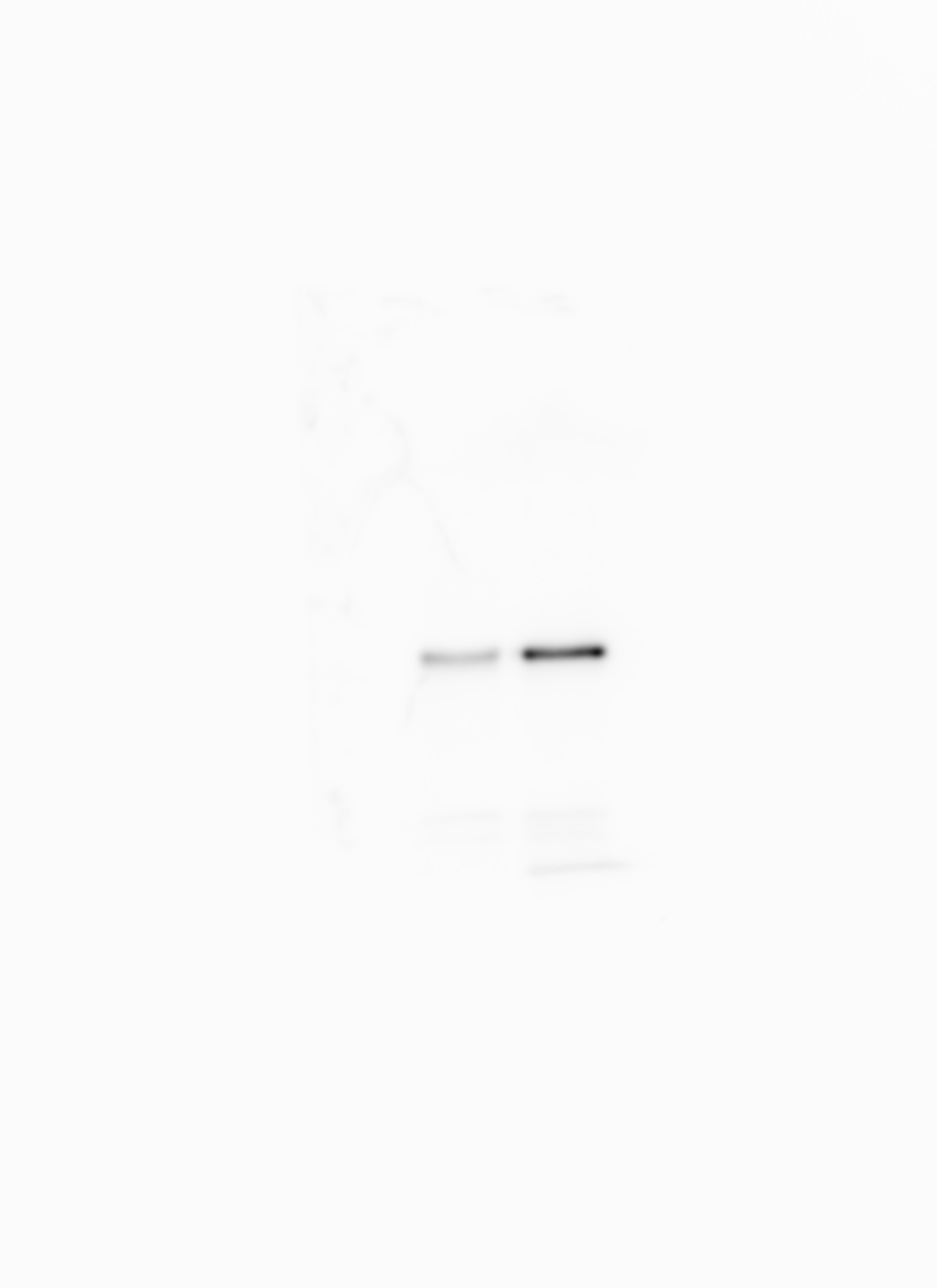

Supplement: Figure 6—source data 6. [file elife-101673-fig6-data6.zip › flot1 raw unedited.tiff]

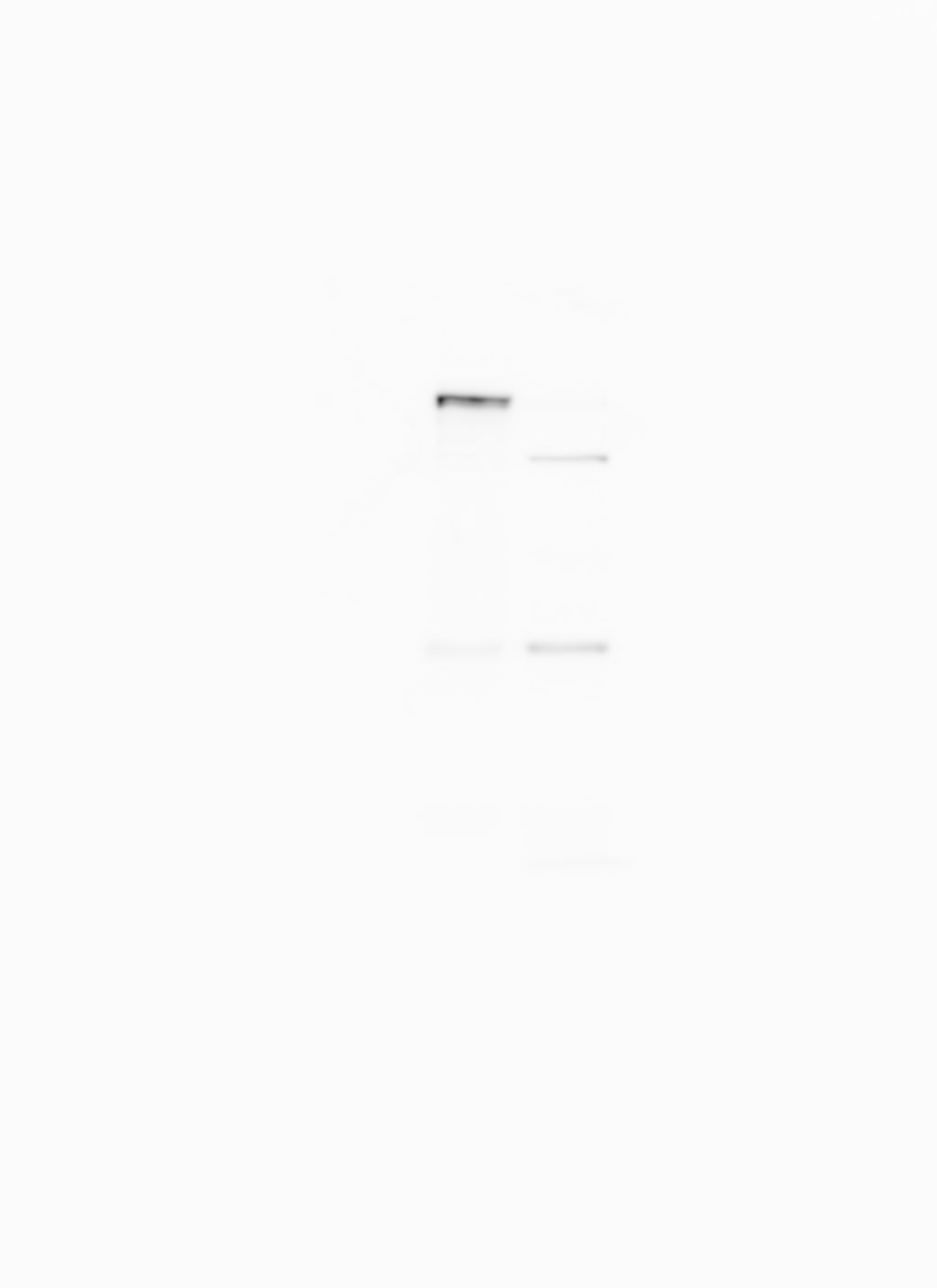

Supplement: Figure 6—source data 6. [file elife-101673-fig6-data6.zip › gm130 raw unedited.tiff]

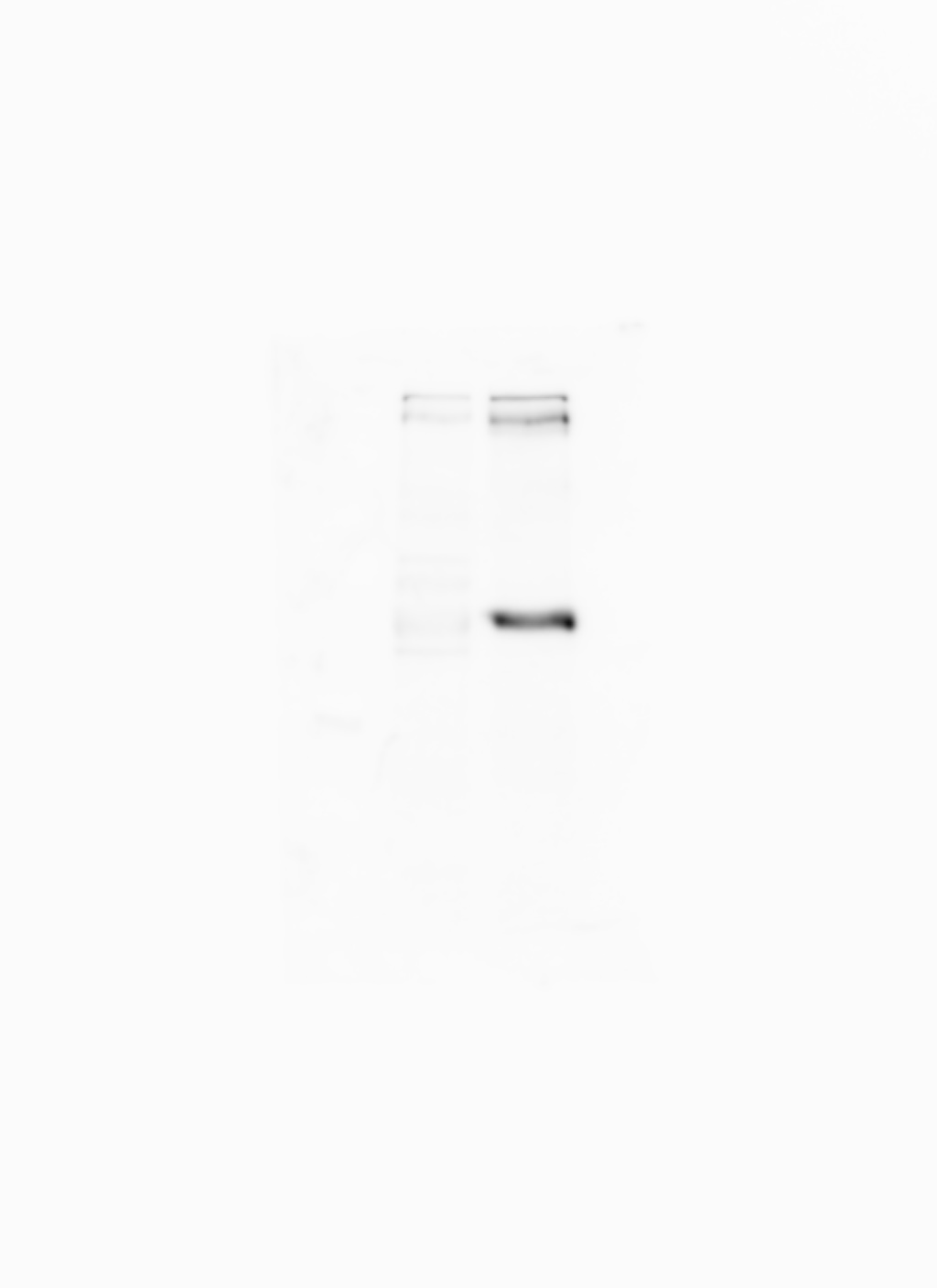

Supplement: Figure 6—source data 6. [file elife-101673-fig6-data6.zip › thsd7a raw unedited.tiff]

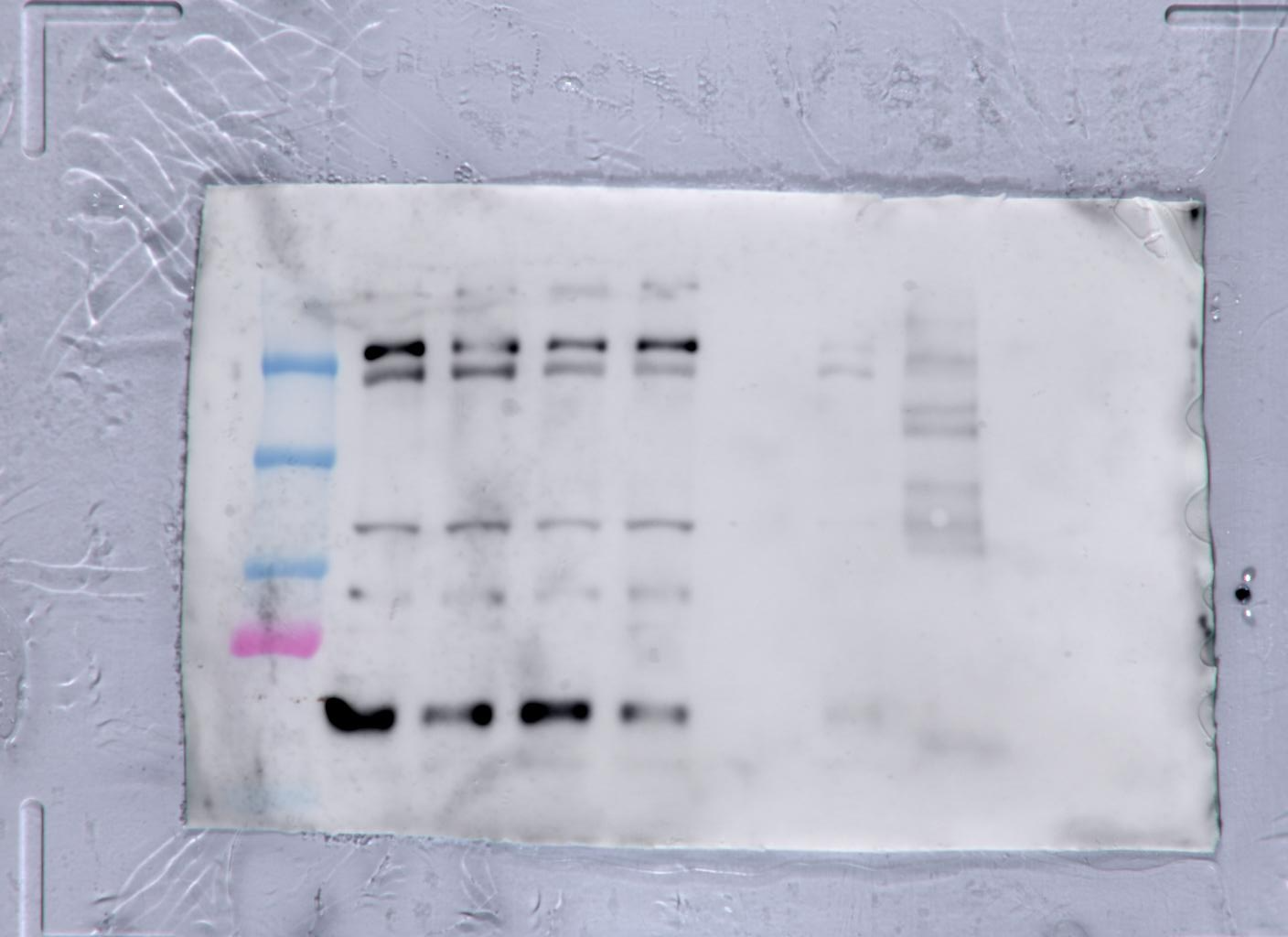

Supplement: Figure 6—source data 8. [file elife-101673-fig6-data8.zip › thsd7a+mwm raw unedited.tiff]

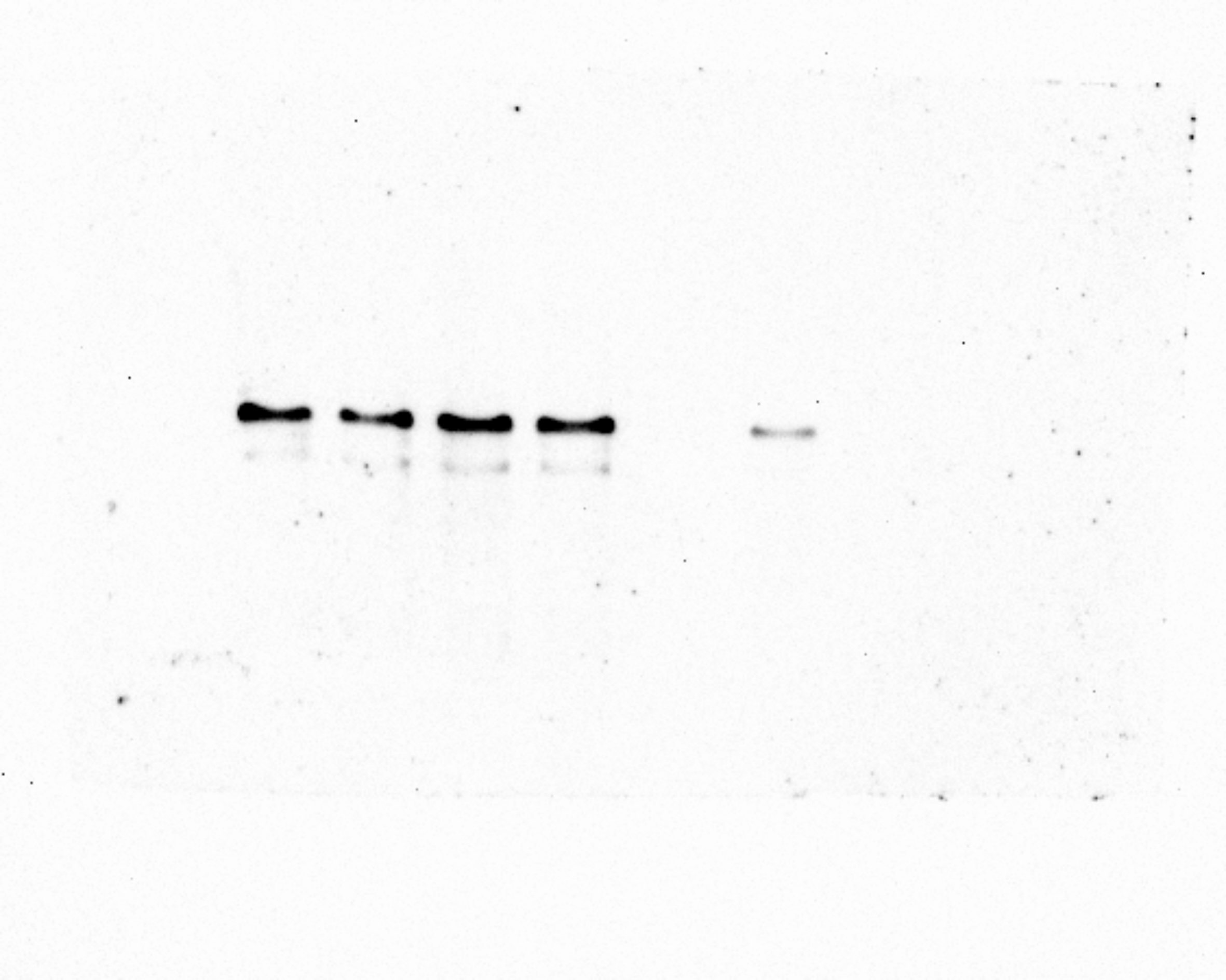

Supplement: Figure 6—source data 8. [file elife-101673-fig6-data8.zip › vinculin raw unedited.tiff]

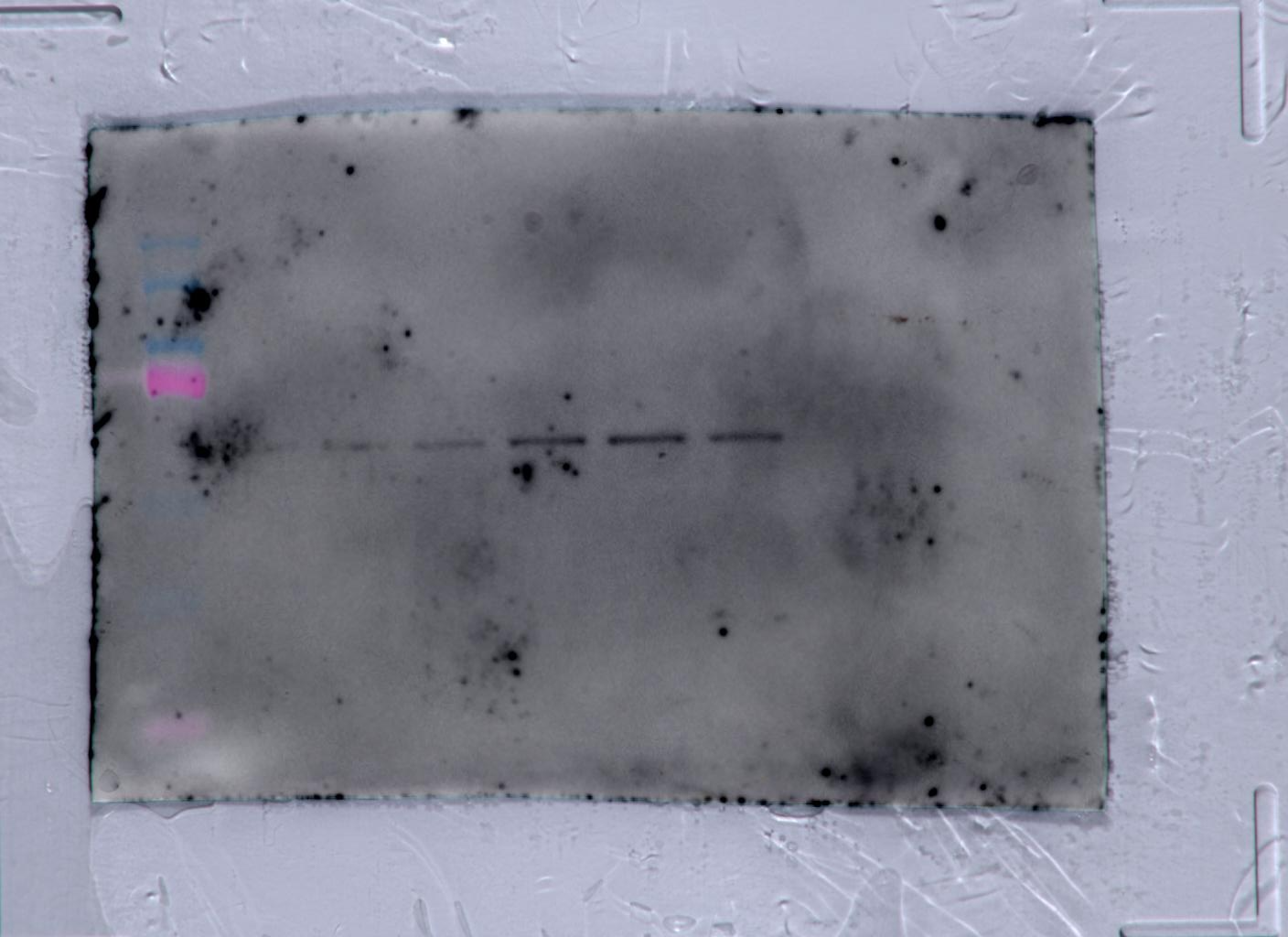

Supplement: Figure 6—figure supplement 1—source data 2. [file elife-101673-fig6-figsupp1-data2.zip › alk1 raw unedited.tiff]

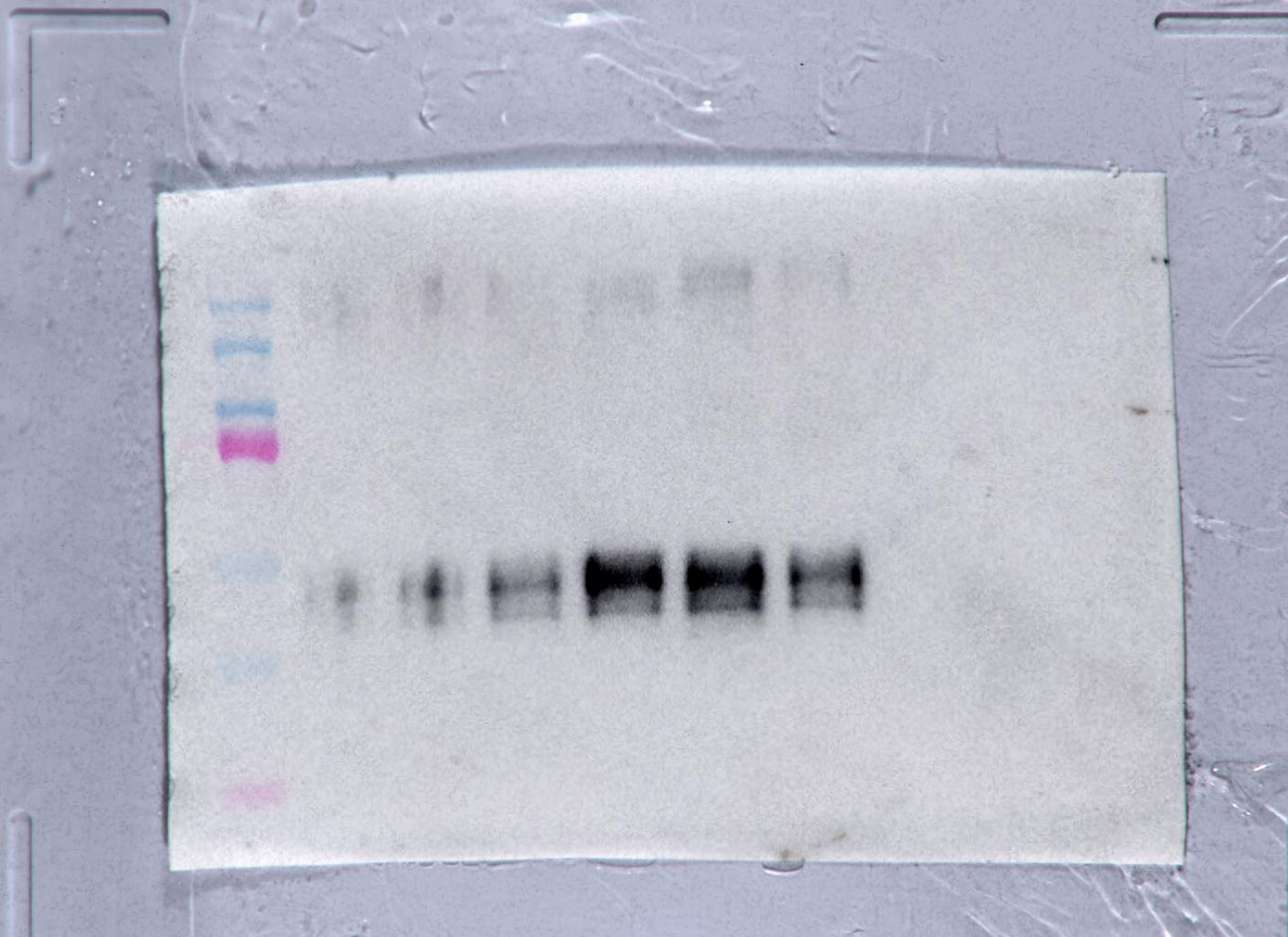

Supplement: Figure 6—figure supplement 1—source data 2. [file elife-101673-fig6-figsupp1-data2.zip › cd63 raw unedited.tiff]

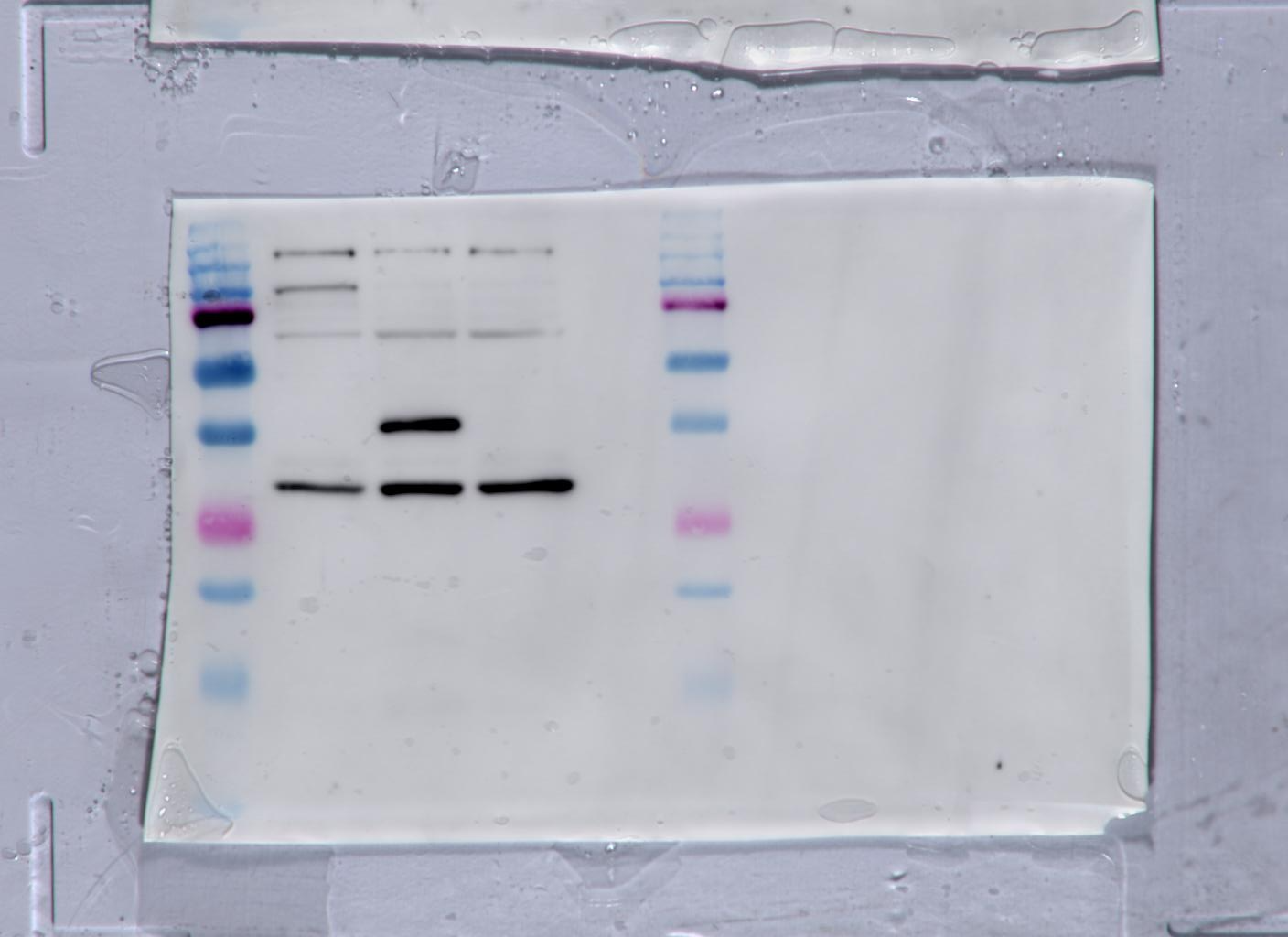

Supplement: Figure 6—figure supplement 1—source data 2. [file elife-101673-fig6-figsupp1-data2.zip › endoglin and tgfb1 raw unedited.tiff]

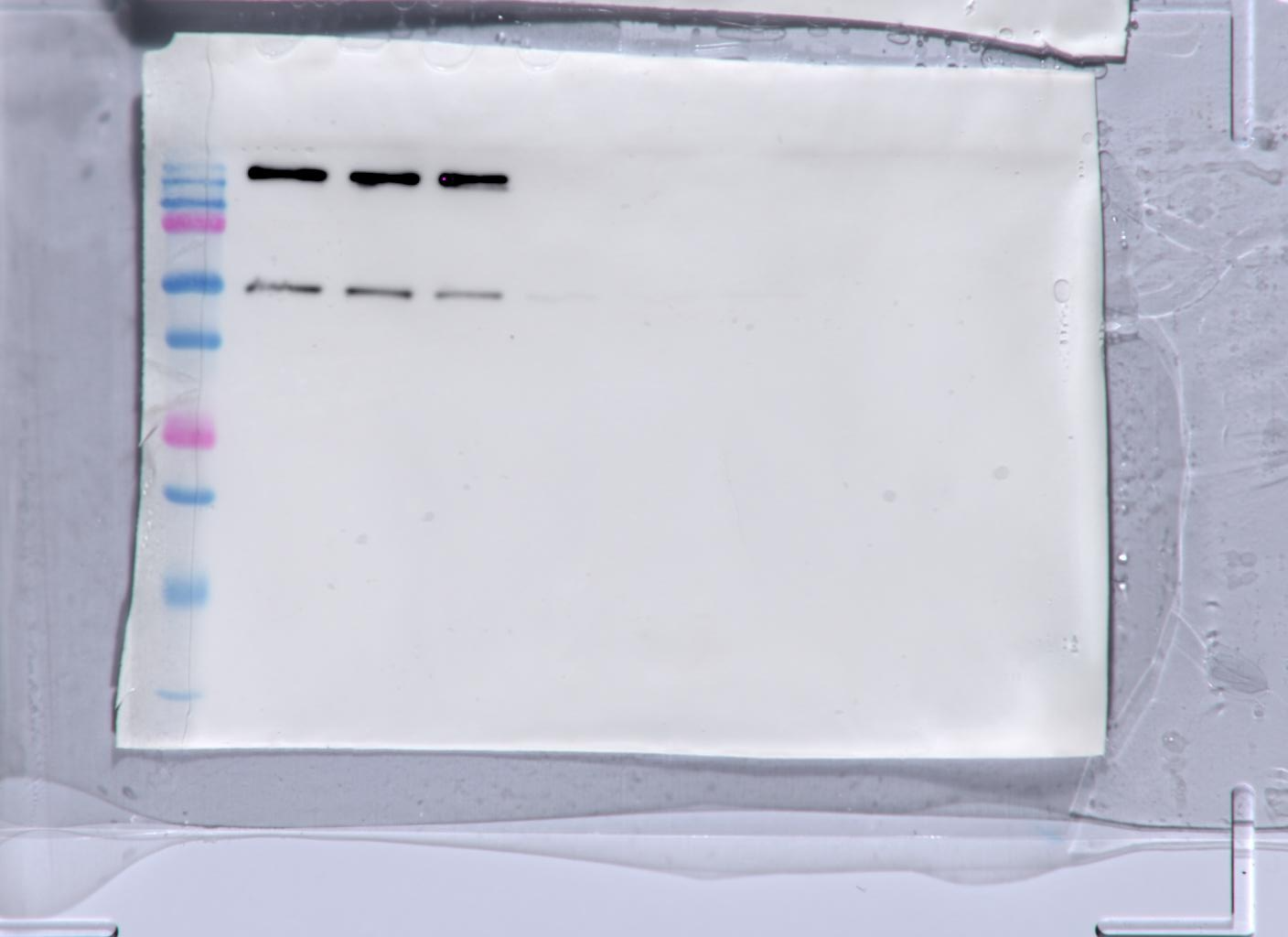

Supplement: Figure 6—figure supplement 1—source data 2. [file elife-101673-fig6-figsupp1-data2.zip › intb1 and flot1 raw unedited.tiff]

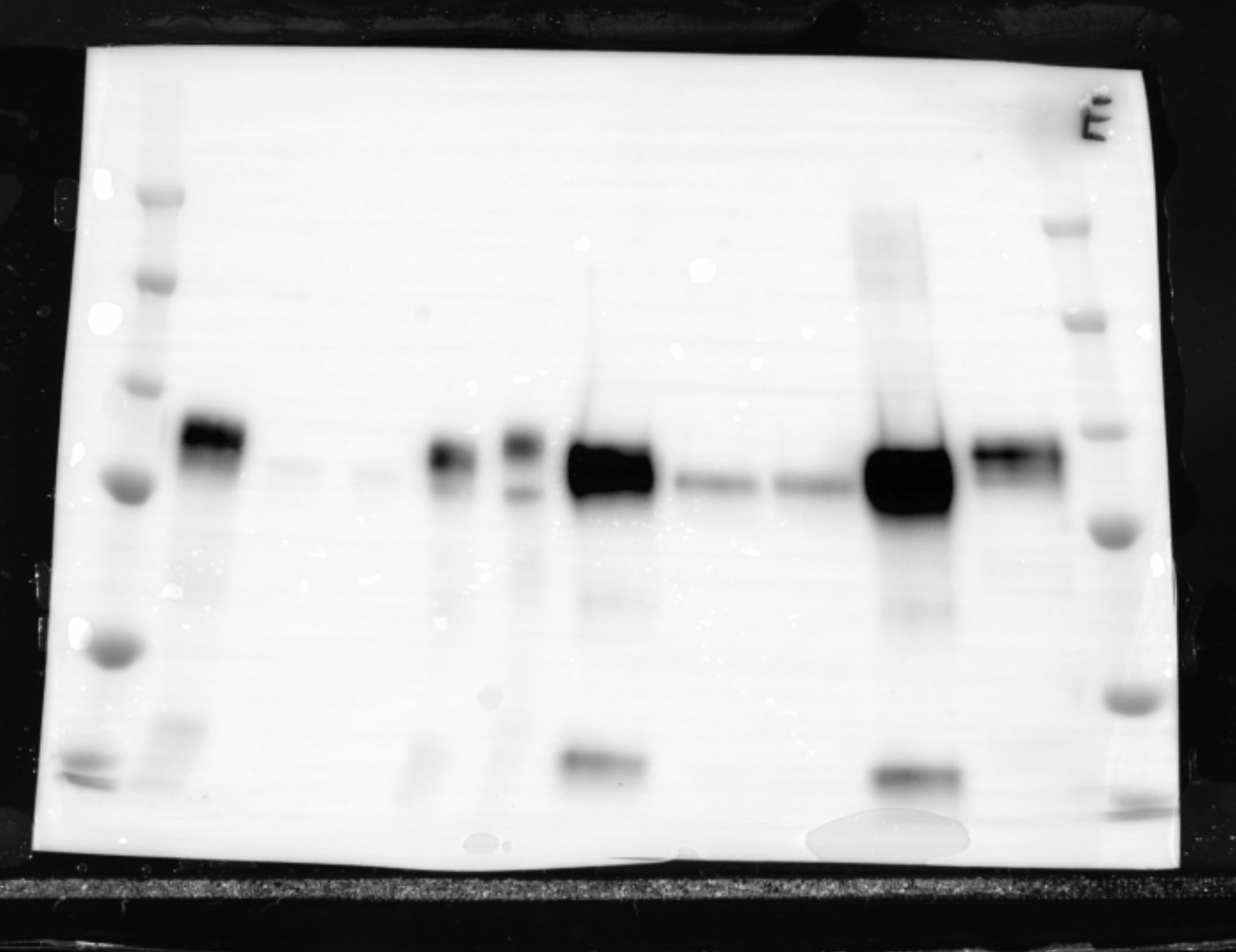

Supplement: Figure 7—source data 2. [file elife-101673-fig7-data2.zip › endoglin+MWM raw unedited.tiff]

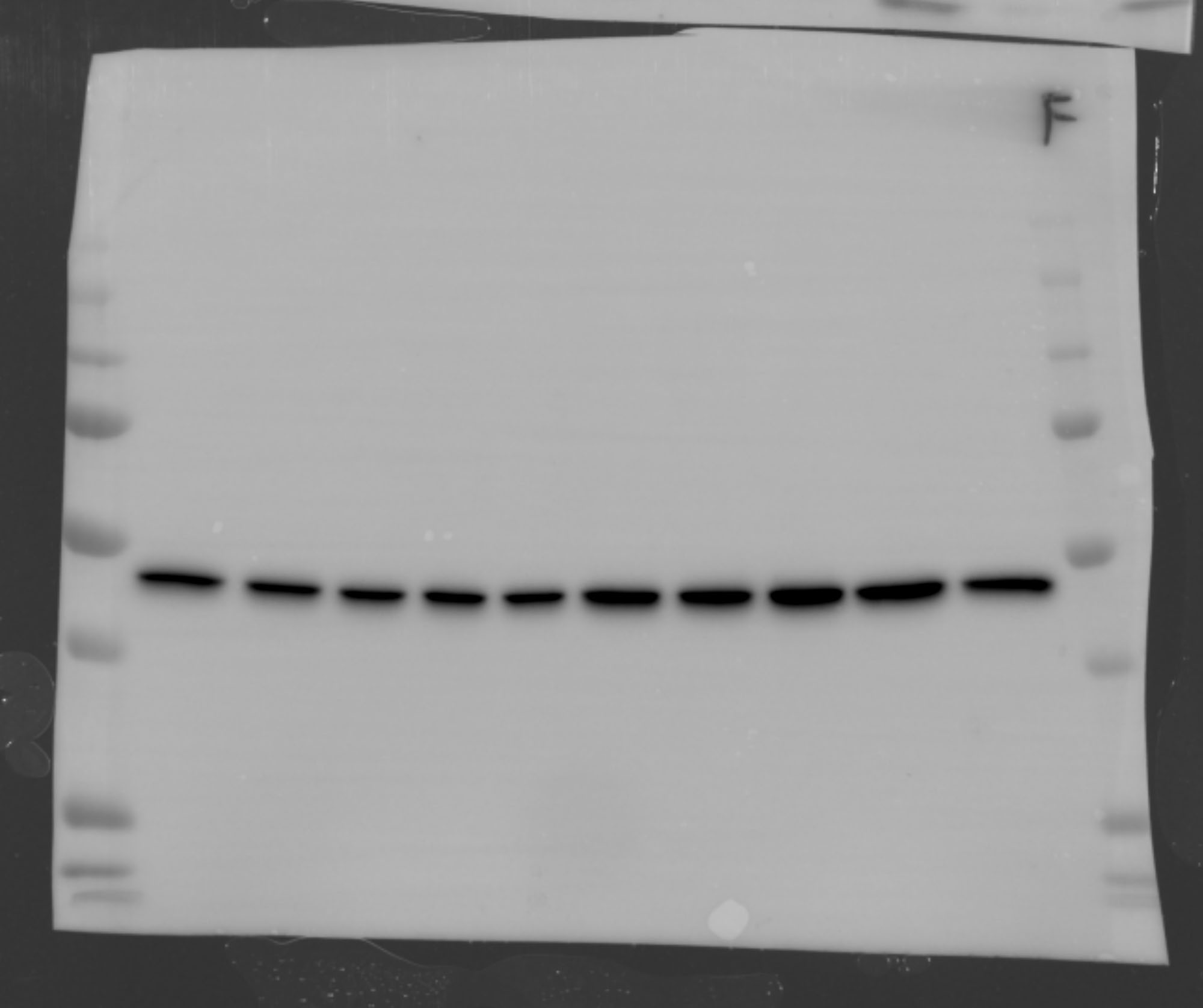

Supplement: Figure 7—source data 2. [file elife-101673-fig7-data2.zip › flot1+MWM raw unedited.tiff]

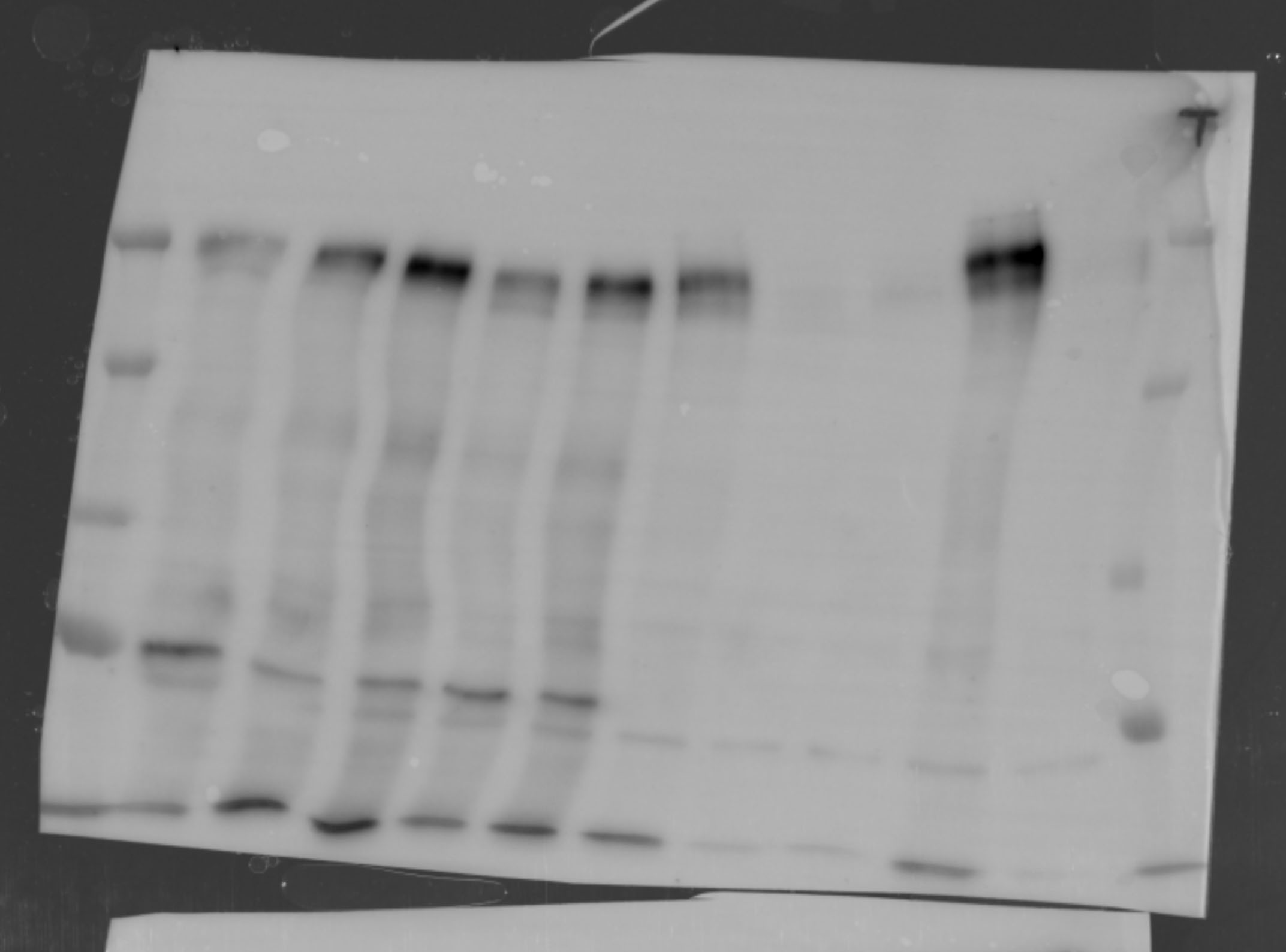

Supplement: Figure 7—source data 2. [file elife-101673-fig7-data2.zip › thsd7a+MWM raw unedited.tiff]

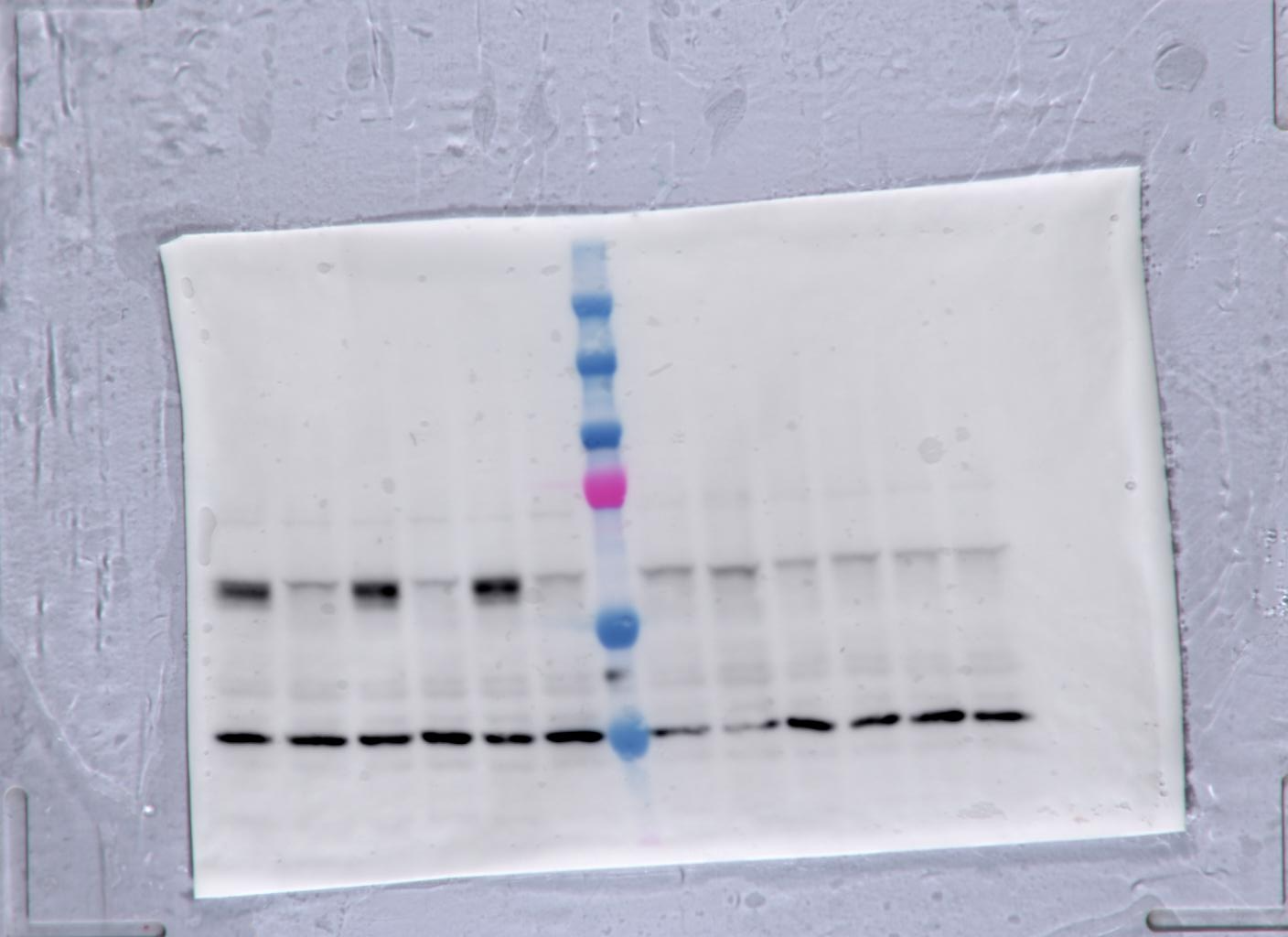

Supplement: Figure 8—figure supplement 1—source data 2. [file elife-101673-fig8-figsupp1-data2.zip › pSmad 159 raw unedited.tiff]

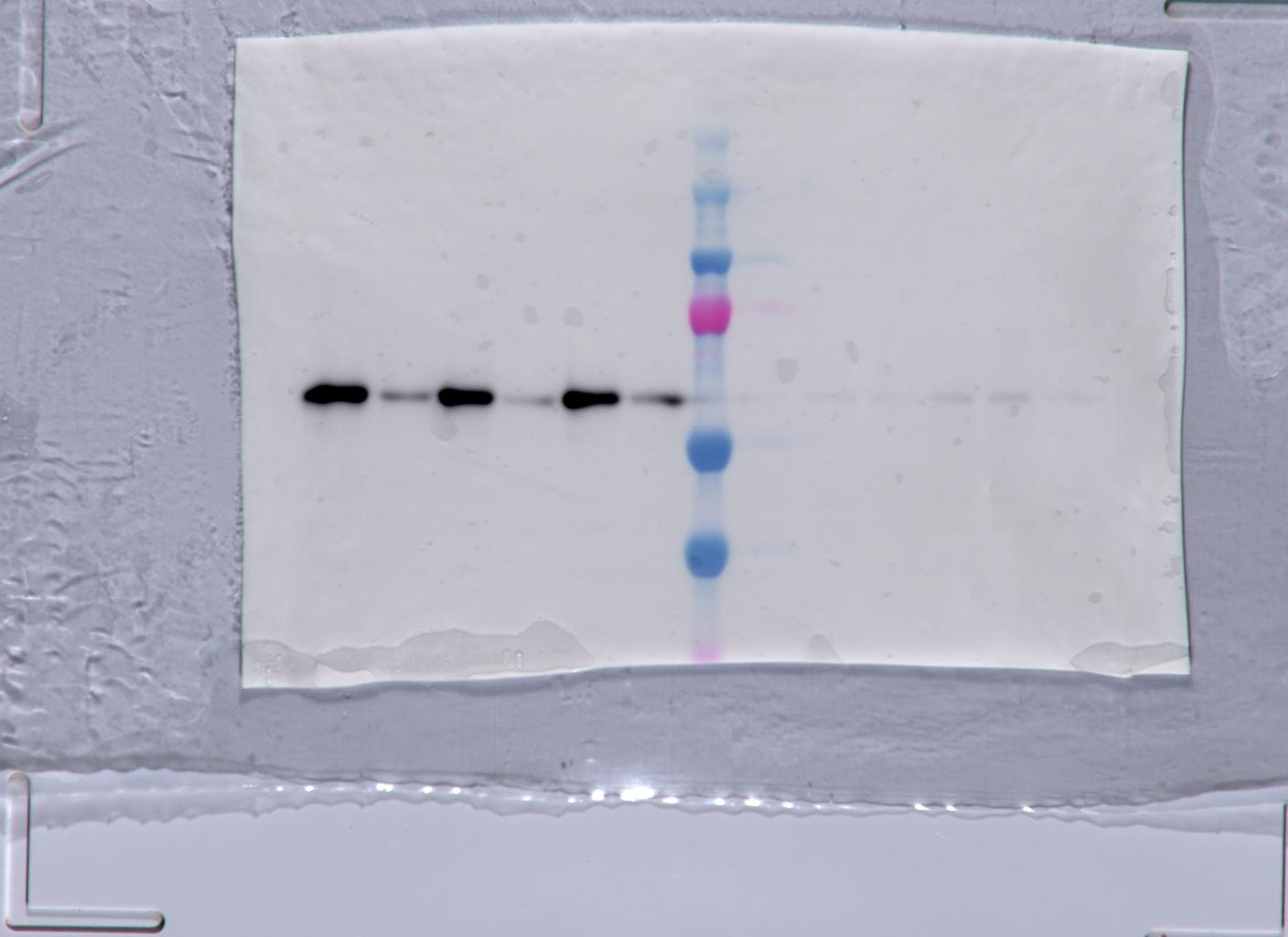

Supplement: Figure 8—figure supplement 1—source data 2. [file elife-101673-fig8-figsupp1-data2.zip › pSmad2 raw unedited.tiff]

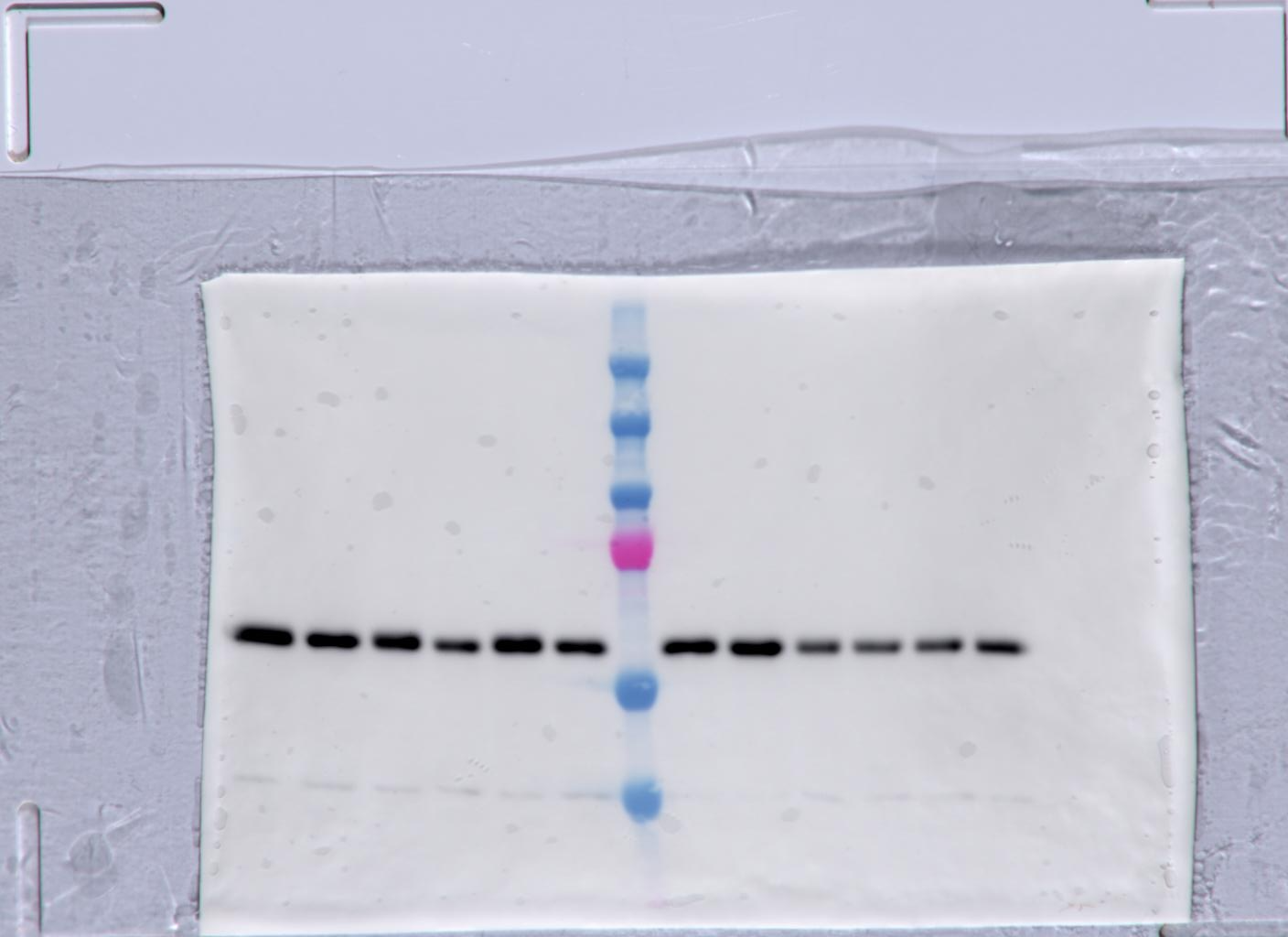

Supplement: Figure 8—figure supplement 1—source data 2. [file elife-101673-fig8-figsupp1-data2.zip › smad1 raw unedited.tiff]

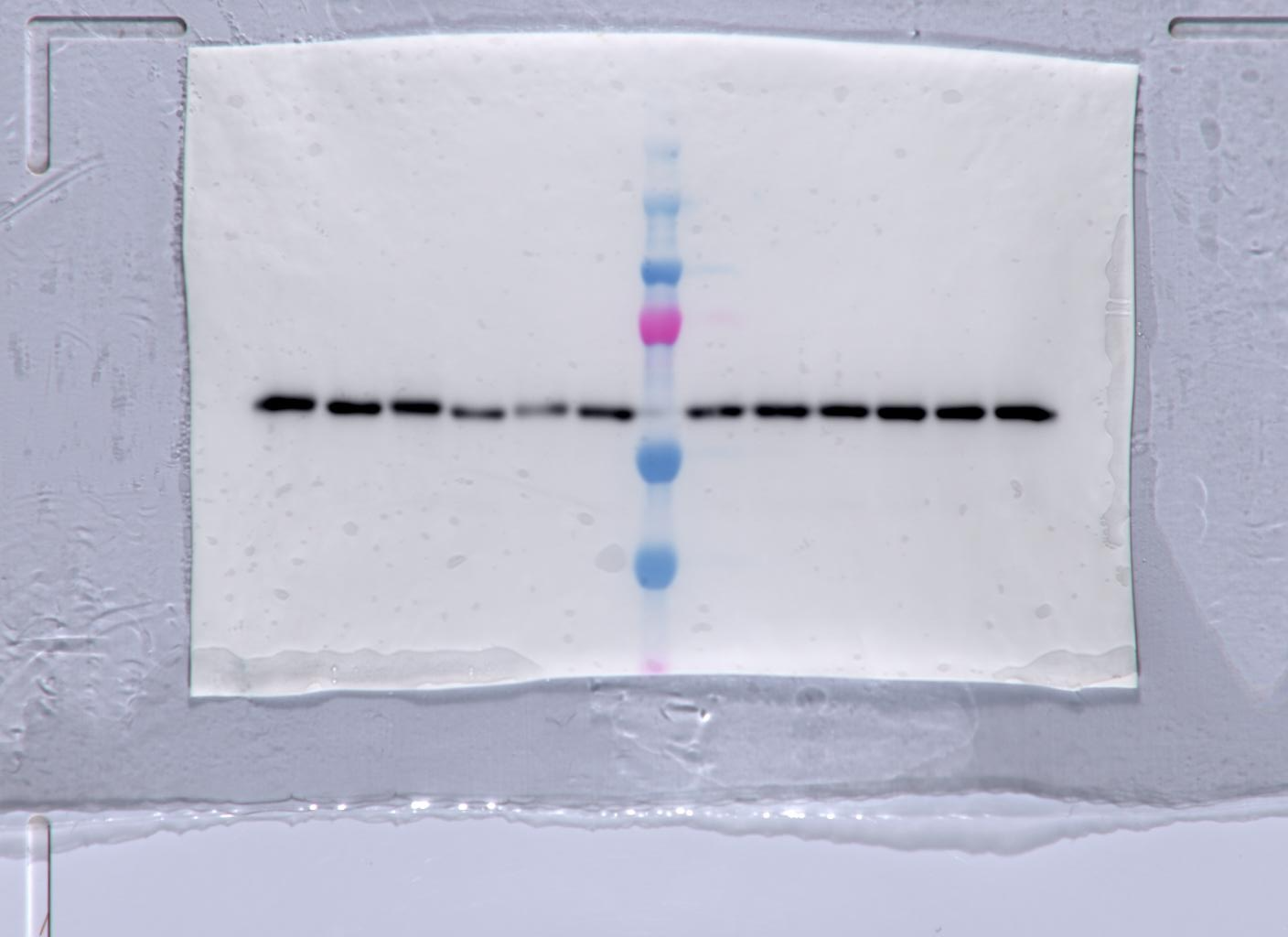

Supplement: Figure 8—figure supplement 1—source data 2. [file elife-101673-fig8-figsupp1-data2.zip › smad2 raw unedited.tiff]
