# Supplementary material for: Secreted exosomes induce filopodia formation
Source: eLife. 2026 Jan 14;13:RP101673. doi: 10.7554/eLife.101673 (PMC12803517; doi:10.7554/eLife.101673)
Supplement: Figure 2—figure supplement 1—source data 3. [file elife-101673-fig2-figsupp1-data3.zip › Figure 2_Figure Supplement 1_Source Data 3.pdf]

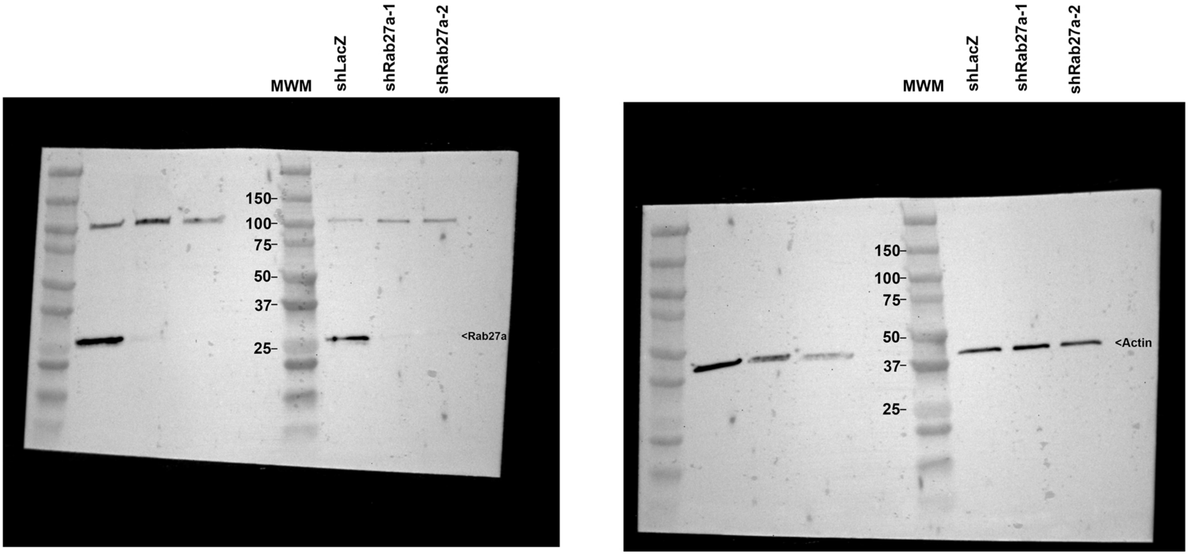

Figure 2, Figure Supplement 1, Source Data 3. Original membranes corresponding to Figure 2 Figure Supplement 1, panel B. Rainbow molecular weight markers were employed. Right side of the blots have the relevant total cell lysate samples.
