# Supplementary material for: Secreted exosomes induce filopodia formation
Source: eLife. 2026 Jan 14;13:RP101673. doi: 10.7554/eLife.101673 (PMC12803517; doi:10.7554/eLife.101673)
Supplement: Figure 2—figure supplement 1—source data 5. [file elife-101673-fig2-figsupp1-data5.zip › Figure 2_Figure Supplement 1_Source Data 5.pdf]

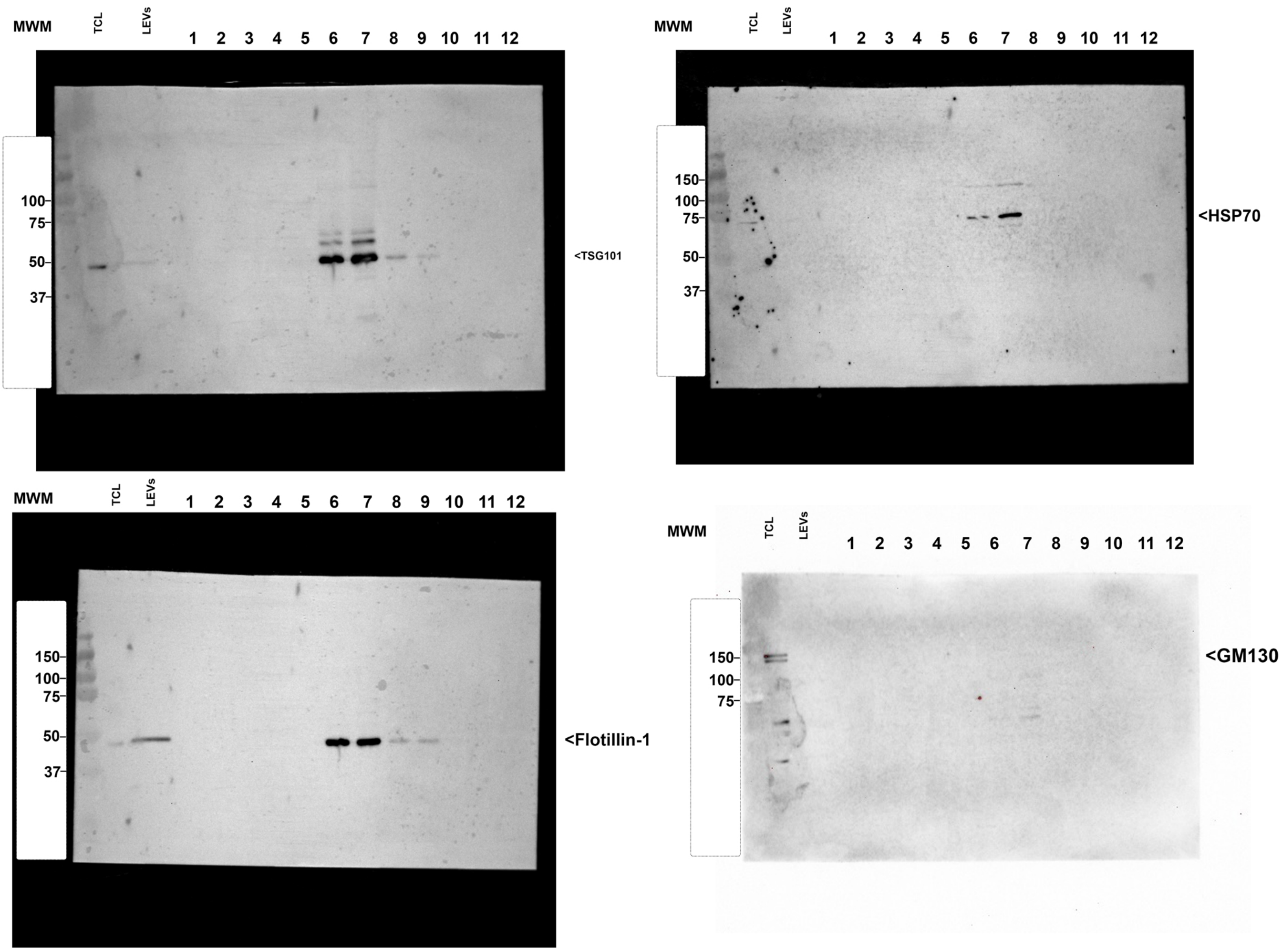

Figure 2, Figure Supplement 1, Source Data 5. Original membranes corresponding to Figure 2 Figure Supplement 1D. Rainbow molecular weight markers were employed. Left lanes show total cell lysate and large extracellular vesicles, then following 12 lanes show 1 mL fractions from the density gradient purification, from least to most dense.
