# Supplementary material for: Secreted exosomes induce filopodia formation
Source: eLife. 2026 Jan 14;13:RP101673. doi: 10.7554/eLife.101673 (PMC12803517; doi:10.7554/eLife.101673)
Supplement: Figure 2—figure supplement 2—source data 1. [file elife-101673-fig2-figsupp2-data1.zip › Figure 2_Figure Supplement 2_Source Data 1.pdf]

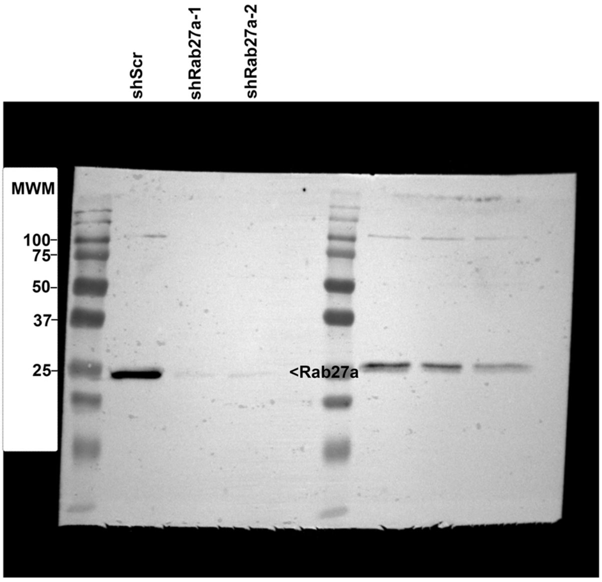

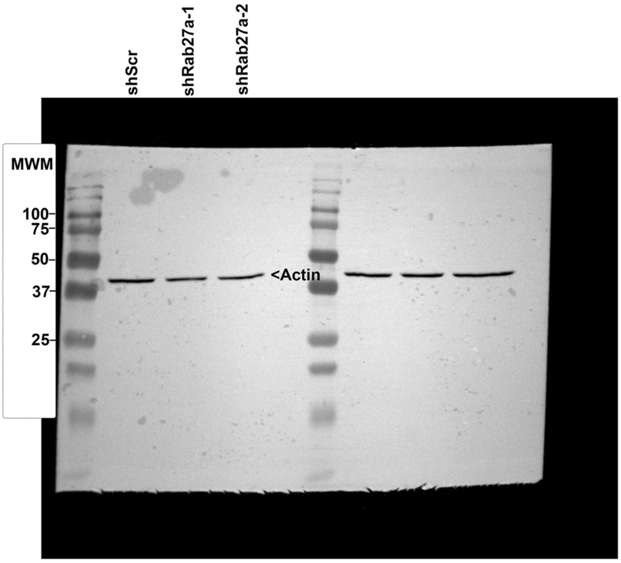

Figure 2, Figure Supplement 2, Source data 2. Original membranes corresponding to Figure 2 Figure Supplement 2, panel A. Rainbow molecular weight markers were employed. Left side of the membranes show relevant total cell lysate samples.
