# Supplementary material for: Secreted exosomes induce filopodia formation
Source: eLife. 2026 Jan 14;13:RP101673. doi: 10.7554/eLife.101673 (PMC12803517; doi:10.7554/eLife.101673)
Supplement: Figure 4—source data 1. [file elife-101673-fig4-data1.zip › Figure 4_Source Data 1.pdf]

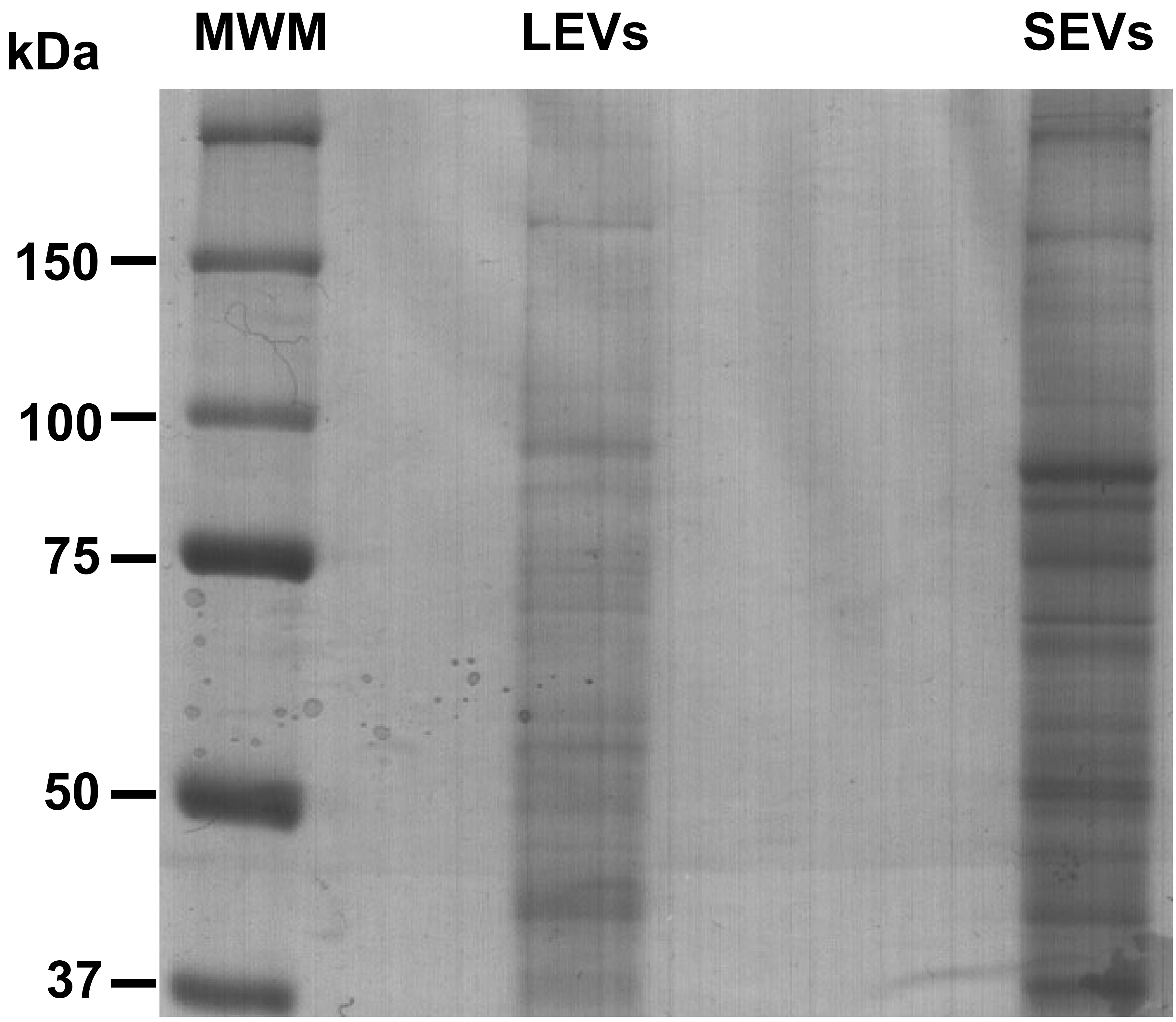

Figure 4, Source Data 1. Original colloidal blue-stained gel corresponding to Figure 4, panel A. Left lane is the molecular weight marker, lane labeled “LEVs” are the large extracellular vesicles, and lane labeled “SEVs” are the small extracellular vesicles.
