# Supplementary material for: Secreted exosomes induce filopodia formation
Source: eLife. 2026 Jan 14;13:RP101673. doi: 10.7554/eLife.101673 (PMC12803517; doi:10.7554/eLife.101673)
Supplement: Figure 4—source data 5. [file elife-101673-fig4-data5.zip › Figure 4_Source Data 5.pdf]

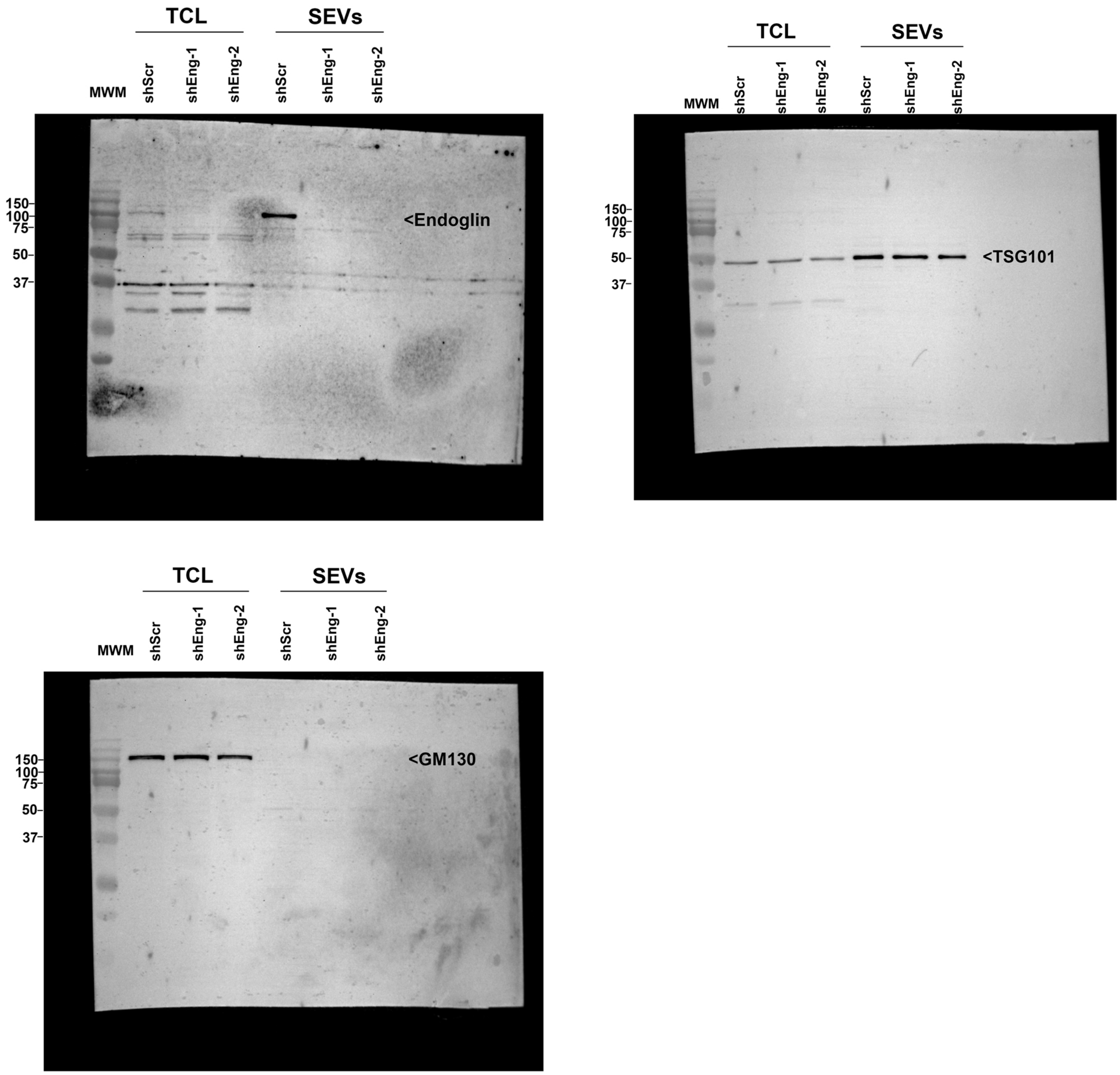

Figure 4, Source Data 5. Original membranes corresponding to Figure 4, panel C. Rainbow molecular weight markers were employed. Samples are total cell lysate and small extracellular vesicles from control and endoglin knockdown cells.
