# Supplementary material for: Secreted exosomes induce filopodia formation
Source: eLife. 2026 Jan 14;13:RP101673. doi: 10.7554/eLife.101673 (PMC12803517; doi:10.7554/eLife.101673)
Supplement: Figure 4—figure supplement 1—source data 1. [file elife-101673-fig4-figsupp1-data1.zip › Figure 4_Figure Supplement 1_Source Data 1.pdf]

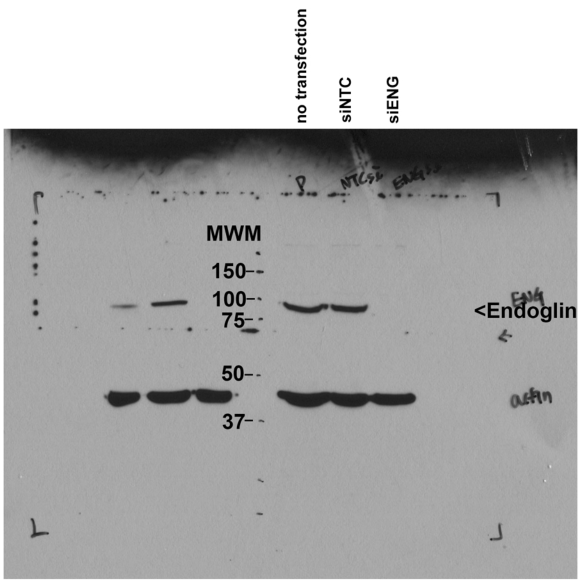

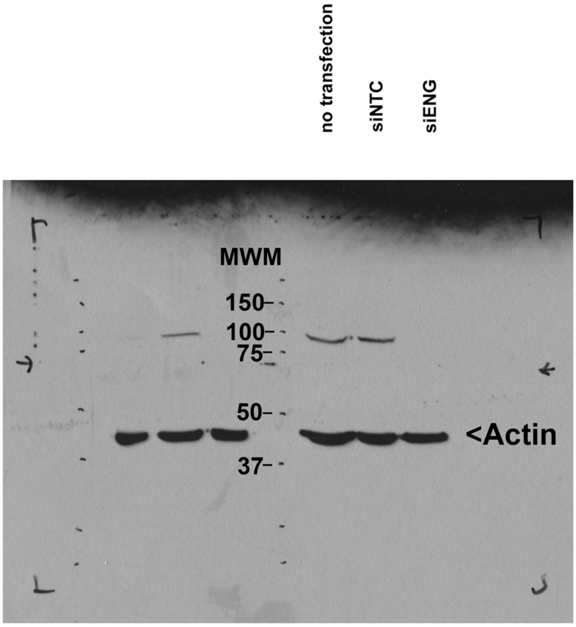

Figure 4, Figure Supplement 1, Source Data 1. Original exposures on film of membranes corresponding to Figure 4 Figure Supplement 1, panel C. Rainbow molecular weight markers were employed. Right side of each film shows relevant total cell lysates that are shown in the final figure panels.
