# Supplementary material for: Secreted exosomes induce filopodia formation
Source: eLife. 2026 Jan 14;13:RP101673. doi: 10.7554/eLife.101673 (PMC12803517; doi:10.7554/eLife.101673)
Supplement: Figure 4—figure supplement 2—source data 1. [file elife-101673-fig4-figsupp2-data1.zip › Figure 4_Figure Supplement 2_Source Data 1.pdf]

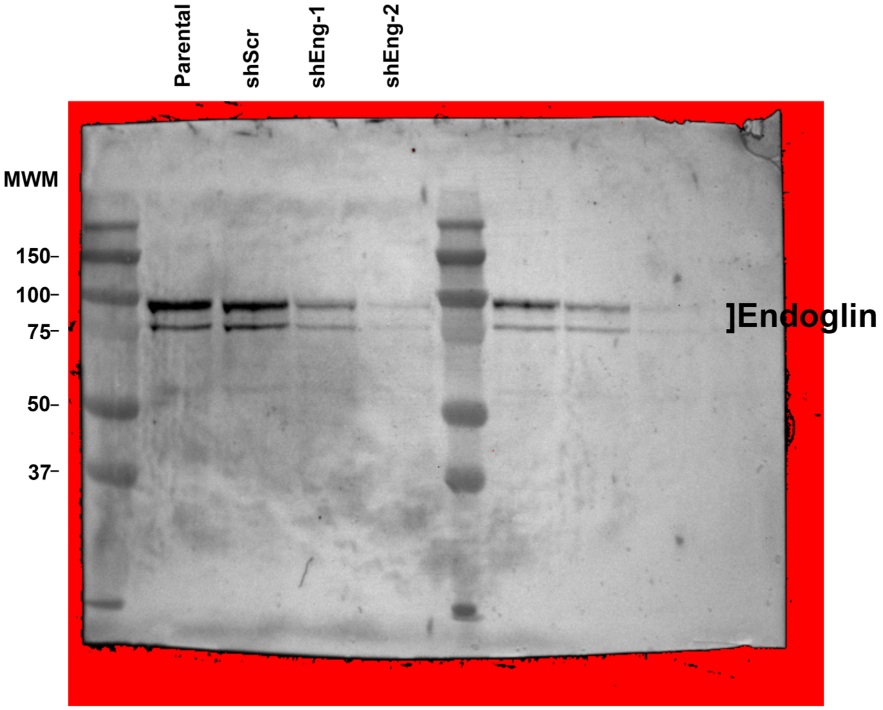

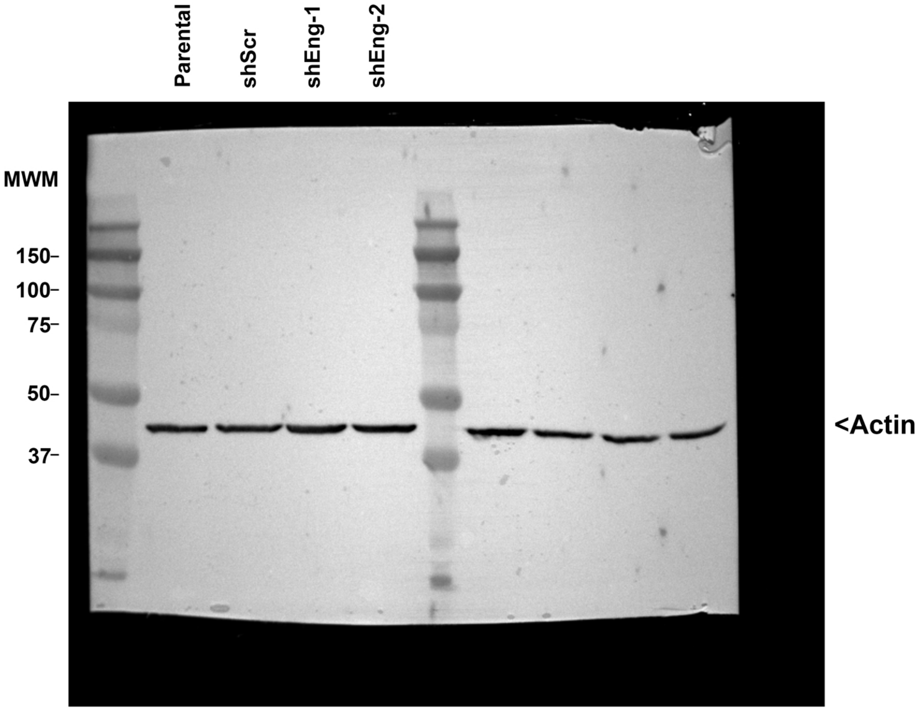

Figure 4, Figure supplement 2, Source data 1. Original membranes corresponding to Figure 4 Figure Supplement 2, panel A. Rainbow molecular weight markers were employed. Left side of the membranes shows the relevant total cell lysate samples that were shown in the final figure panels.
