# Supplementary material for: Secreted exosomes induce filopodia formation
Source: eLife. 2026 Jan 14;13:RP101673. doi: 10.7554/eLife.101673 (PMC12803517; doi:10.7554/eLife.101673)
Supplement: Figure 6—source data 1. [file elife-101673-fig6-data1.zip › Figure 6_Source Data 1.pdf]

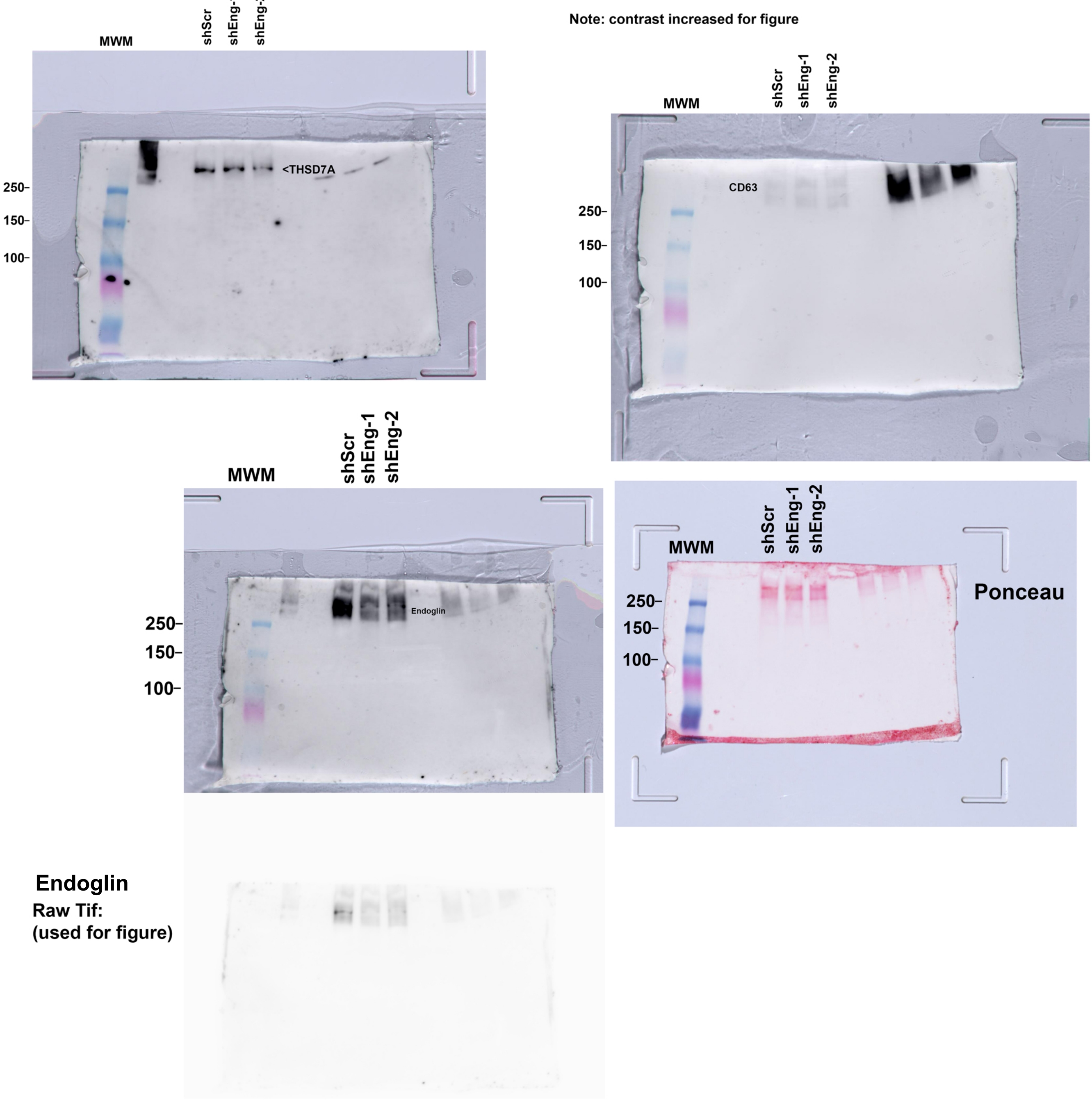


**Figure 6 Source Data 1.** Original membranes corresponding to Figure 6, panel A. Rainbow molecular weight markers were employed. Relevant total cell lysate samples are labeled above membranes. Last membrane shows the total protein using Ponceau stain.
