# Supplementary material for: Secreted exosomes induce filopodia formation
Source: eLife. 2026 Jan 14;13:RP101673. doi: 10.7554/eLife.101673 (PMC12803517; doi:10.7554/eLife.101673)
Supplement: Figure 6—source data 3. [file elife-101673-fig6-data3.zip › Figure 6_Source Data 3.pdf]

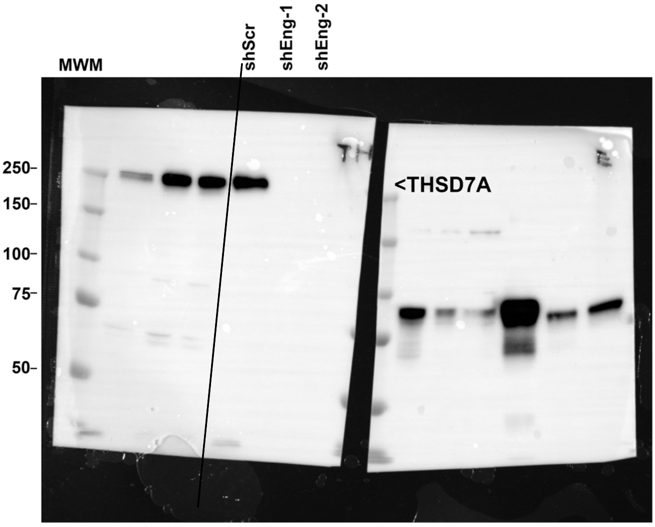

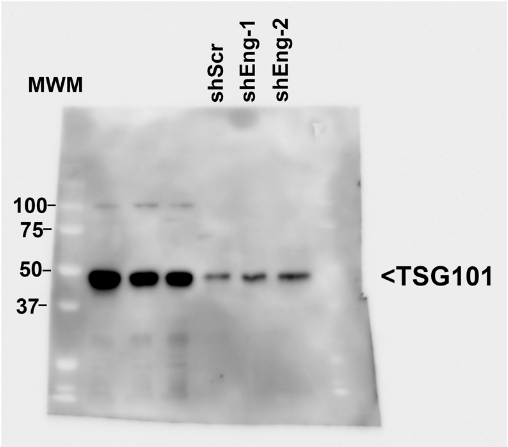

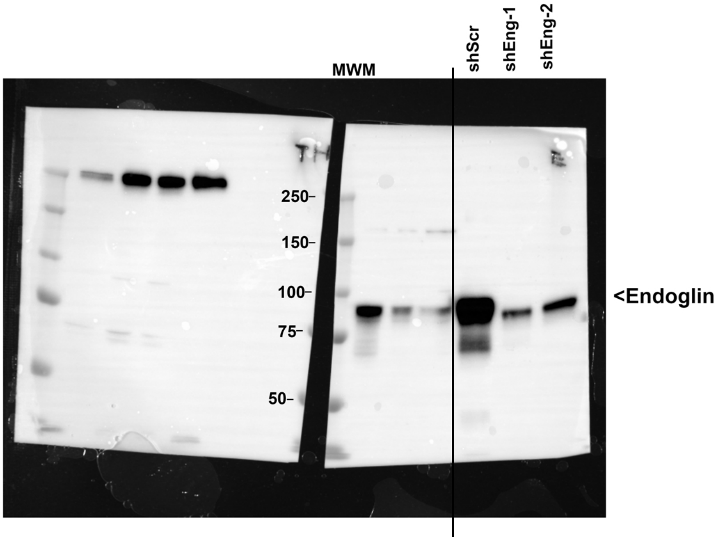

Figure 6, Source data 3. Original membranes corresponding to Figure 6, panel B. Rainbow molecular weight markers were employed. Vertical lines are added for ease of aligning sample name to band. THSD7A and Endoglin were scanned in the same file, and are shown here duplicated and labeled separately.
