# Supplementary material for: Secreted exosomes induce filopodia formation
Source: eLife. 2026 Jan 14;13:RP101673. doi: 10.7554/eLife.101673 (PMC12803517; doi:10.7554/eLife.101673)
Supplement: Figure 6—source data 5. [file elife-101673-fig6-data5.zip › Figure 6_Source Data 5.pdf]

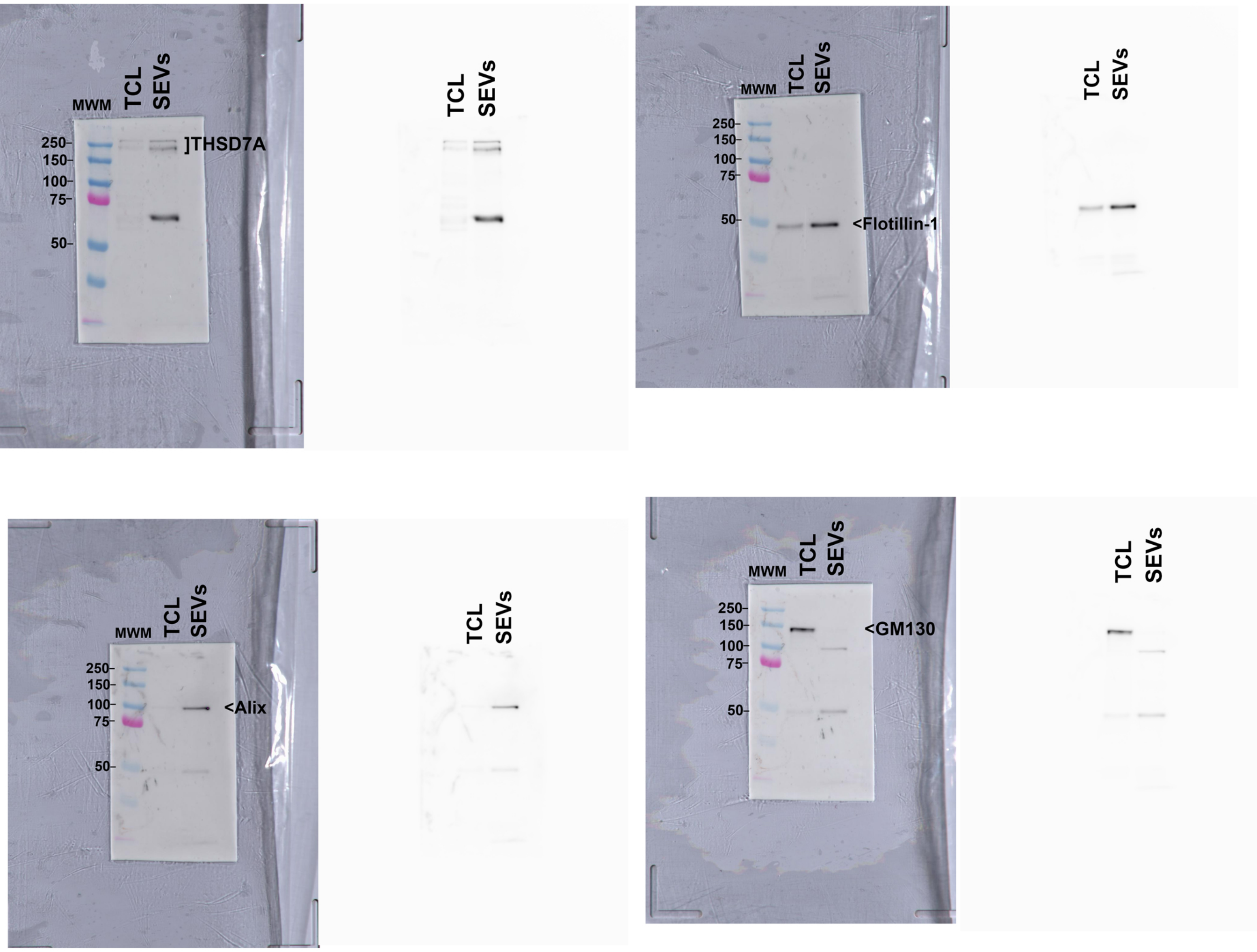

Figure 6, Source Data 5. Original membranes corresponding to Figure 6, panel C. Rainbow molecular weight markers were employed. Membrane scans shown as TIF only (right images) and TIF with the molecular weight marker scan overlayed (left images).
