# Supplementary material for: Secreted exosomes induce filopodia formation
Source: eLife. 2026 Jan 14;13:RP101673. doi: 10.7554/eLife.101673 (PMC12803517; doi:10.7554/eLife.101673)
Supplement: Figure 6—source data 7. [file elife-101673-fig6-data7.zip › Figure 6_Source Data 7.pdf]

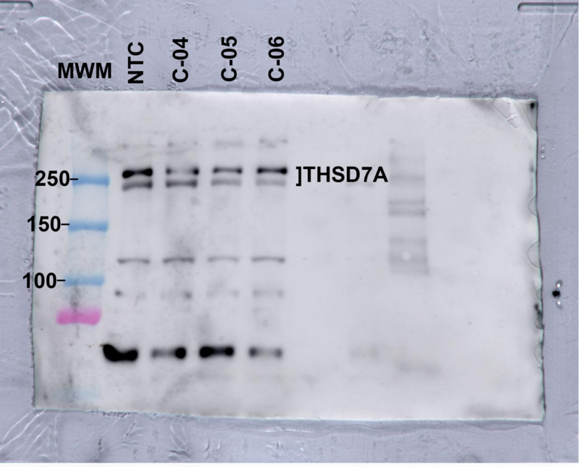

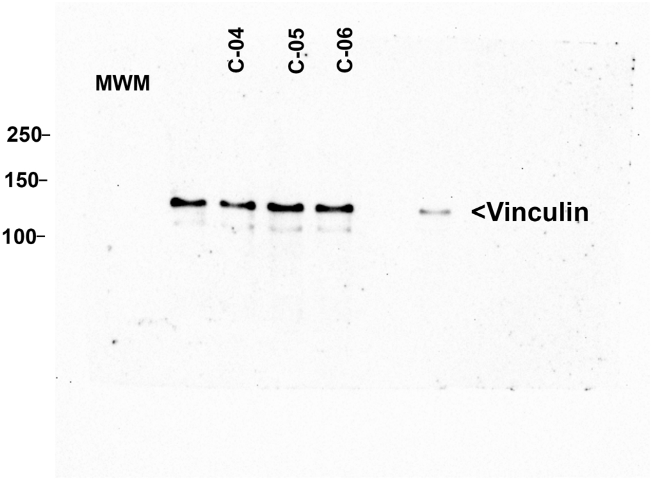

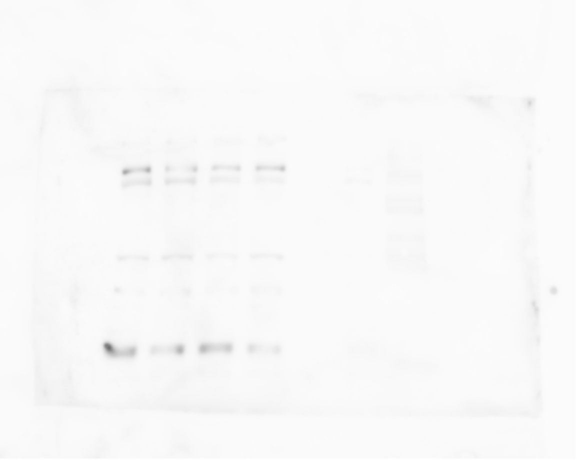

Figure 6, Source Data 7. Original membranes corresponding to Figure 6, panel E. Rainbow molecular weight markers were employed. For THSD7A, bottom image shows TIF only and top image shows TIF with molecular weight marker scan overlayed on top.
