# Supplementary material for: Secreted exosomes induce filopodia formation
Source: eLife. 2026 Jan 14;13:RP101673. doi: 10.7554/eLife.101673 (PMC12803517; doi:10.7554/eLife.101673)
Supplement: Figure 6—figure supplement 1—source data 1. [file elife-101673-fig6-figsupp1-data1.zip › Figure 6_Figure Supplement 1_Source Data 1.pdf]

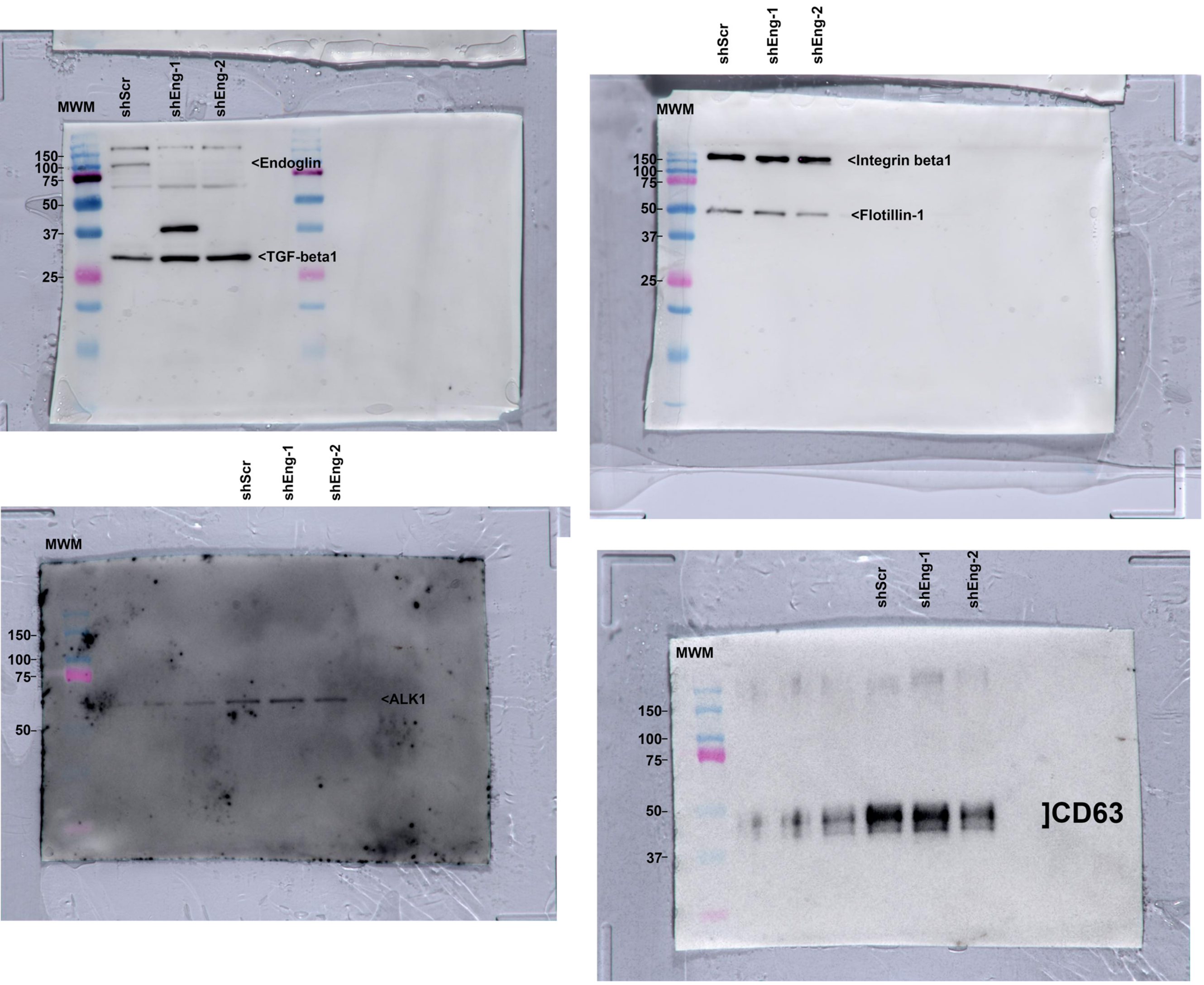


**Figure 6, Figure Supplement 1, Source Data 1.** Original membranes corresponding to Figure 6 Figure Supplement 1, panel A. Rainbow molecular weight markers were employed. Relevant lanes on the bottom two membranes are lanes 5-7 (labeled with cell type). Relevant samples are total cell lysates of the control and endoglin knockdown cell lines. Note that the top two blots simultaneously show two relevant bands, probed with two different primary and secondary antibodies.
