# Supplementary material for: Secreted exosomes induce filopodia formation
Source: eLife. 2026 Jan 14;13:RP101673. doi: 10.7554/eLife.101673 (PMC12803517; doi:10.7554/eLife.101673)
Supplement: Figure 7—source data 1. [file elife-101673-fig7-data1.zip › Figure 7_Source Data 1.pdf]

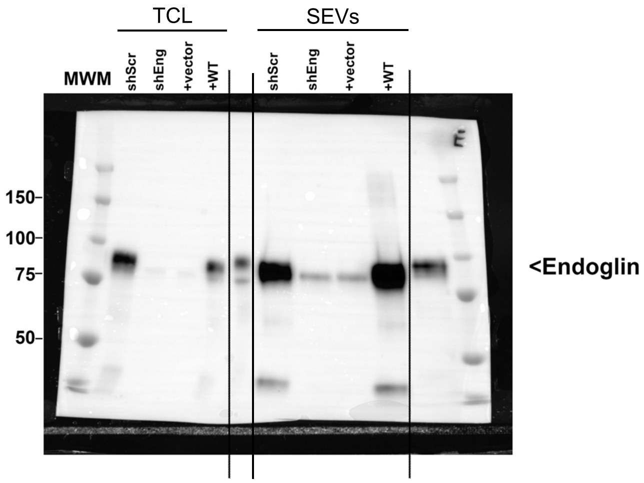

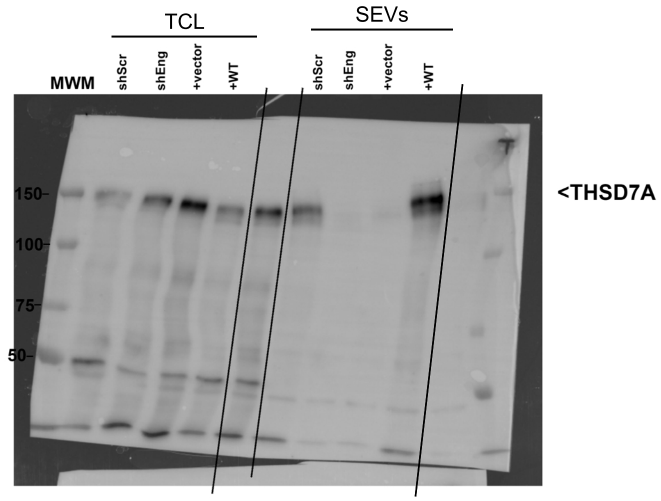

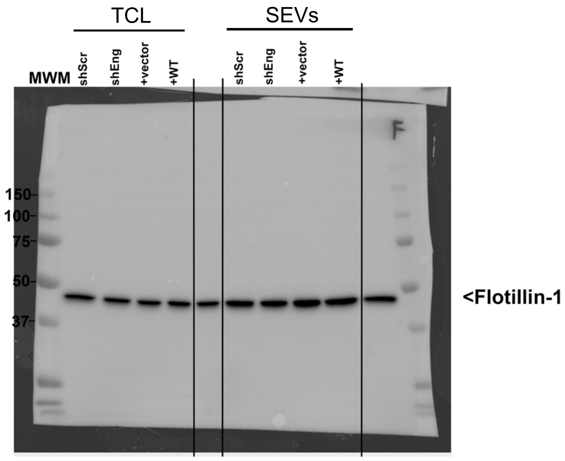

Figure 7, source data 1. Original membranes corresponding to Figure 7, panel A. Vertical lines on blots show where cropping was done for final figure images. Rainbow molecular weight markers were employed. Samples on left side of each blot are the total cell lysates and samples on right side of each blot are the small extracellular vesicles.
