# Supplementary material for: Secreted exosomes induce filopodia formation
Source: eLife. 2026 Jan 14;13:RP101673. doi: 10.7554/eLife.101673 (PMC12803517; doi:10.7554/eLife.101673)
Supplement: Figure 8—figure supplement 1—source data 1. [file elife-101673-fig8-figsupp1-data1.zip › Figure 8_Figure Supplement 1_Source Data 1.pdf]

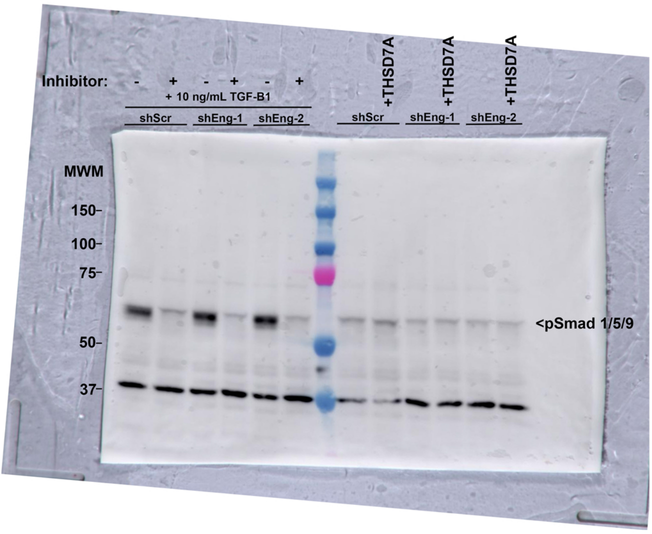

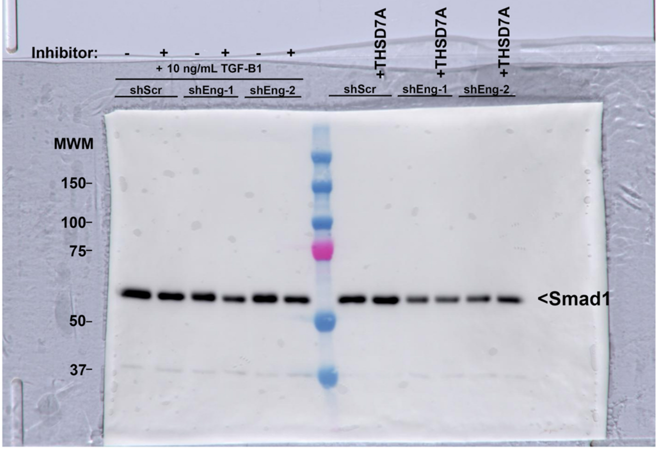

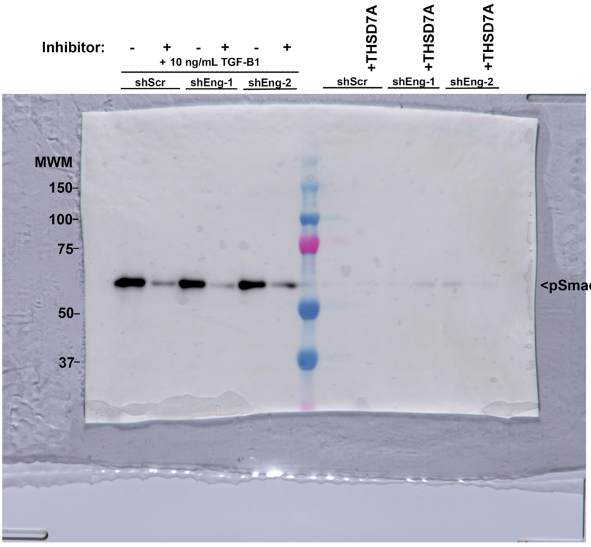

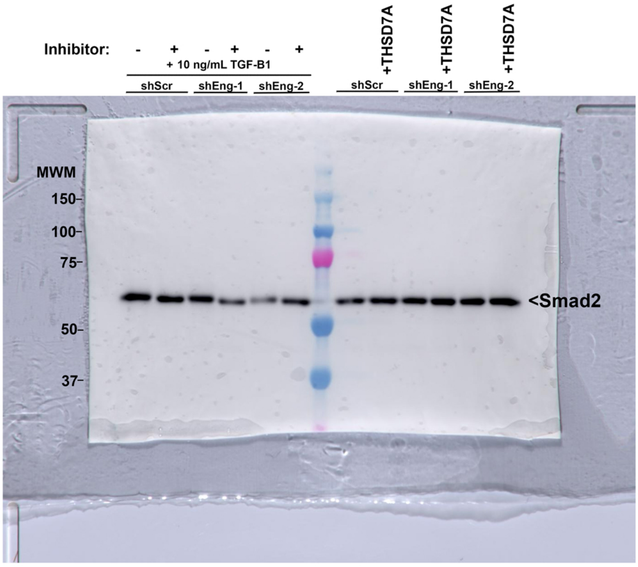

Figure 8, Figure Supplement 1, Source Data 1. Original membranes corresponding to Figure 8 Figure Supplement 1. Rainbow molecular weight markers were employed. Labels above blots denote the relevant total cell lysate samples and treatment conditions.
